# Slip on a mapped normal fault for the 28^th^ December 1908 Messina earthquake (Mw 7.1) in Italy

**DOI:** 10.1038/s41598-019-42915-2

**Published:** 2019-04-24

**Authors:** M. Meschis, G. P. Roberts, Z. K. Mildon, J. Robertson, A. M. Michetti, J. P. Faure Walker

**Affiliations:** 1 Department of Earth and Planetary Sciences, Birkbeck, University of London, London, UK; 2 School of Geography, Earth and Environmental Sciences, University of Plymouth, Plymouth, UK; 3 Università degli Studi dell’Insubria, Como, Italy; 4 Institute for Risk and Disaster Reduction, UCL, London, UK

**Keywords:** Tectonics, Geomorphology, Natural hazards

## Abstract

The 28th December 1908 Messina earthquake (Mw 7.1), Italy, caused >80,000 deaths and transformed earthquake science by triggering the study of earthquake environmental effects worldwide, yet its source is still a matter of debate. To constrain the geometry and kinematics of the earthquake we use elastic half-space modelling on non-planar faults, constrained by the geology and geomorphology of the Messina Strait, to replicate levelling data from 1907–1909. The novelty of our approach is that we (a) recognise the similarity between the pattern of vertical motions and that of other normal faulting earthquakes, and (b) for the first time model the levelling data using the location and geometry of a well-known offshore capable fault. Our results indicate slip on the capable fault with a dip to the east of 70° and 5 m dip-slip at depth, with slip propagating to the surface on the sea bed. Our work emphasises that geological and geomorphological observations supporting maps of capable non-planar faults should not be ignored when attempting to identify the sources of major earthquakes.

## Introduction

**Figure 1:**
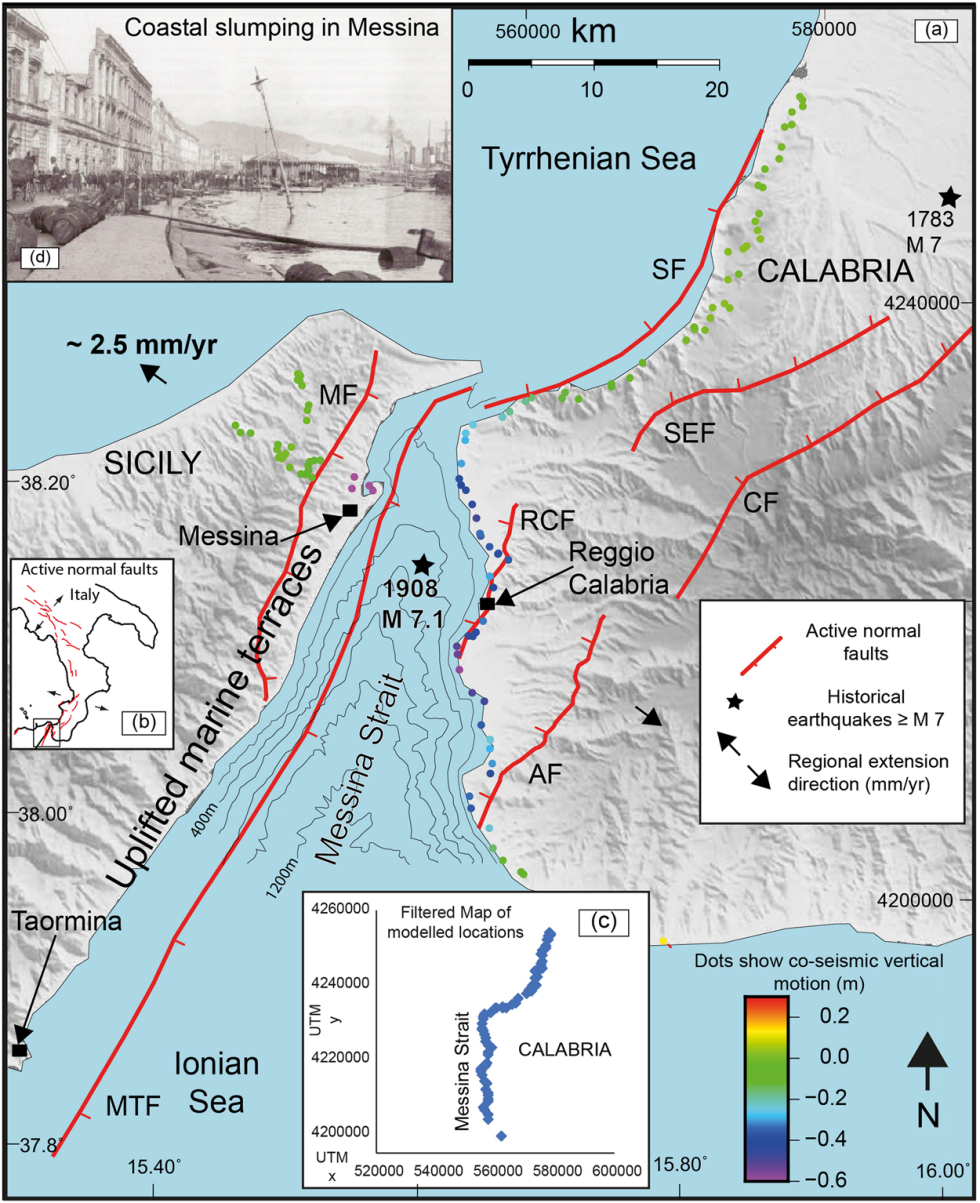
Map of the Messina Strait with well-known Quaternary normal faults^7,9^. Coloured dots represent the co-seismic vertical movement mapped by Loperfido (1909). Messina Fault (MF); Messina-Taormina Fault (MTF); Armo Fault (AF); Reggio Calabria Fault (RCF); Sant’Eufemia Fault (SEF); Cittanova Fault (CF); Scilla Fault (SF). Panel (a) is located in (**b**). (**c**) Filtered levelling data used in the modelling. (**d**) Port of Messina town affected by coastal slumping after the earthquake; photo was published by ref.^12^ and it is available to the following website: http://historyofgeology.fieldofscience.com/2010/12/28-december-1908-earthquake-of-messina.html.

**Figure 2:**
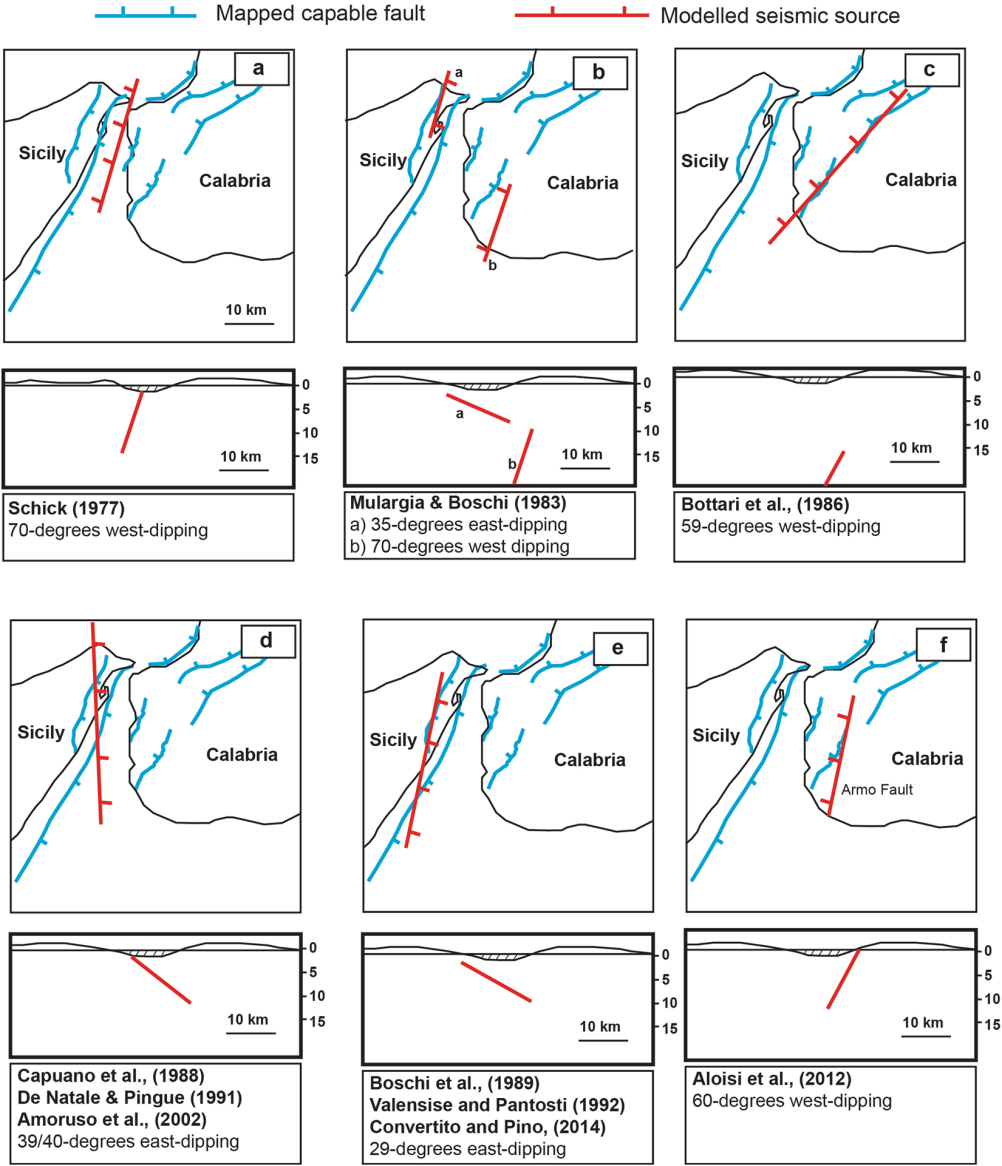
Summary of fault geometries used in previous published attempts to model the geodetic levelling dataset, with a comparison to the mapped faults in the region^56–58^.

**Figure 3:**
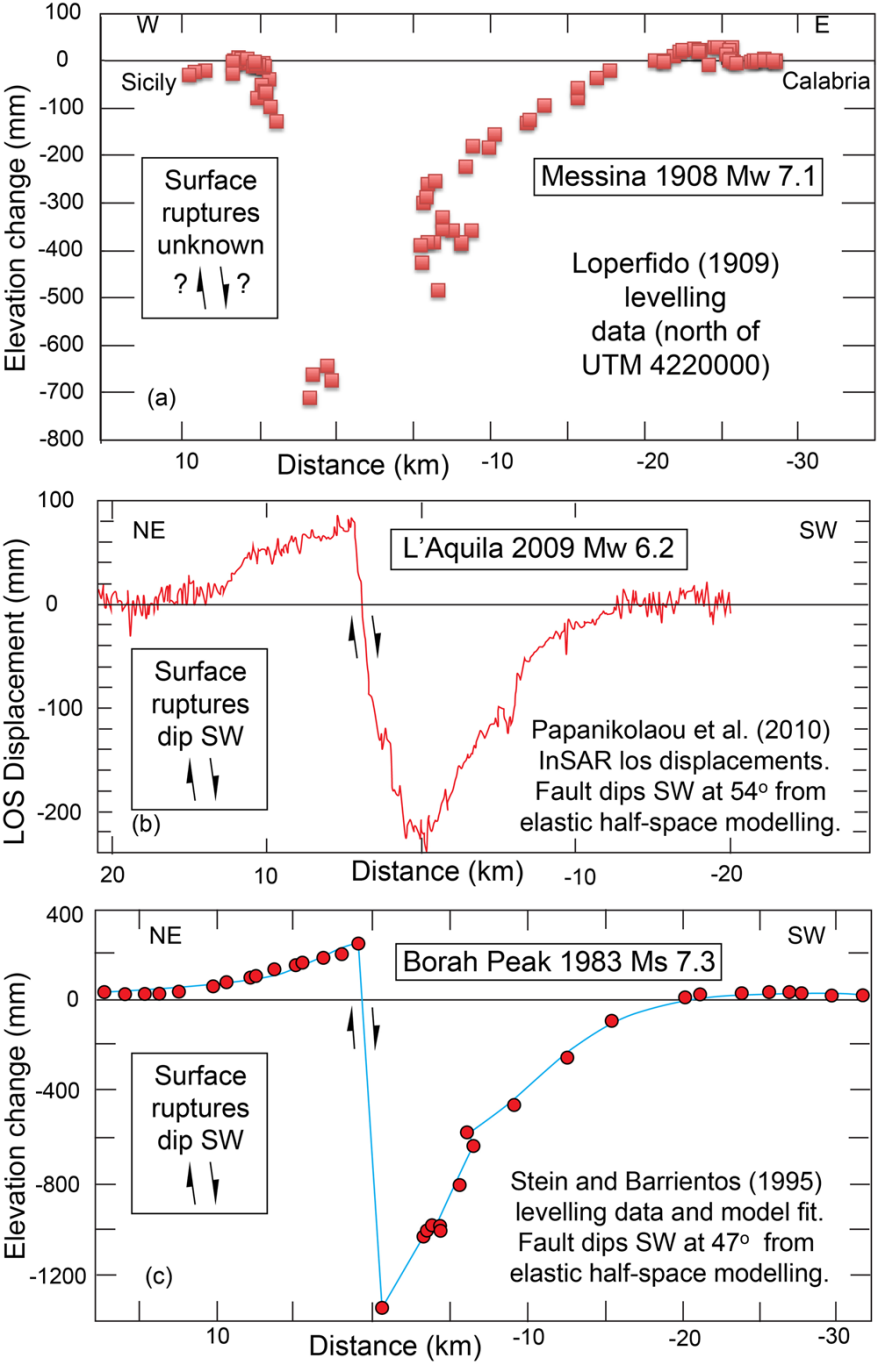
Comparison of observed co-seismic elevation changes for three normal faulting earthquakes, where the dip direction of the surface rupture is known for only two of the examples. (**a**) the 1908 Messina Earthquake; (**b**) the 2009 L’Aquila Earthquake; (**c**) the 1983 Borah Peak Earthquake.

The 28^th^ December 1908 Messina earthquake (Mw 7.1) is the most destructive 20^th^ and 21^st^ century earthquake in Europe, with a death toll of >80,000^1,2^, yet the geometry and kinematics of the fault that ruptured are still a source of debate. It was one of the first earthquakes in Europe in the instrumental period, transforming the study of seismicity by triggering interest in earthquake environmental effects (EEE) that we now know are crucial for understanding the geometry and kinematics of a seismic source^3^. The epicentre was located in the Messina Strait graben, consistent with mapped environmental effects, and deformed Quaternary and Holocene geology^1,4–10^ (Fig. 1). In the absence of a robust focal mechanism^11,12^, or clear evidence of surface rupture^3^, the literature contains several suggestions for the source. However, the modelled sources in the literature^11,13–19^ do not closely coincide with faults identified through detailed geological and geomorphological mapping (Fig. 2). Instead they include both high and low angle, emergent and blind normal faults, in places crossing the coastlines where no faults are mapped in the geology, or having different strikes to mapped faults. In contrast, well-mapped high-angle capable faults around the Messina Strait, located both onshore and offshore, are known to coincide with offsets of basement stratigraphy and control the location of sedimentary basins^9,20^ (Figs 1 and 2). These offsets will have developed due to repeated faulting which offsets the surface through time, so the fact that they have not been modelled in detail is a clear omission in the study of this major earthquake. Plotting of the levelling data as a function of distance E-W reveals the potential importance of east-dipping capable faults (Fig. 3a). This plot reveals, even before modelling, that the pattern of uplift and subsidence strongly resembles that of other large normal faulting earthquakes whose relatively steep source fault dips and dip directions are well known from mapped surface ruptures and epicentre-to-rupture distances^21,22^. This compelling observation suggests that steep, east-dipping seismic sources should be investigated for the 1908 example, and we note that some candidate mapped capable faults have this geometry (Fig. 1). Additionally, previous modelling attempts have used simple planar fault geometries that do not replicate the complexity of the mapped traces of capable faults. We have included non-planar faults whose curvilinear fault traces continue to depth with a cylindrical geometry, by using published methods^23^.

With the above in mind, here we attempt to constrain the location, dip and slip of the fault that ruptured using levelling measurements from 1907–1909^4,24,25^.

## Geological and Seismological Background

The Messina Strait separates Sicily from Calabria in southern Italy, and is a down-faulted area between inward-dipping Quaternary normal faults^7,9,10,26^ (Fig. 1). In general, the normal faults offset thrust sheets of Palaeozoic metamorphic and igneous rocks that were intercalated during Alpine thrusting, during and after the Oligocene-Miocene, revealed by over-thrusts of gneisses and schists onto Oligocene-Miocene flysch sediments^26,27^. Capable normal faults onshore are well-known and mapped^7,9,28^ (Fig. 2), and those offshore are constrained with seismic reflection and bathymetric data^9^. Some studies suggest the existence of an offshore structure, the so-called “Taormina Fault”, that offsets the pre-Pleistocene basement, and propagated upwards to produce a fault-related syncline along its trace from Messina town to Taormina town (herein named “The Messina-Taormina Fault, MTF)^10,28–31^ (Fig. 1), actively deforming sequences of Late Quaternary marine terraces and Holocene coastal notches^28,30,31^. In contrast, other studies advocate less confidence concerning offshore fault locations^32^. However, it is known that offshore active Quaternary faults with footwall uplift are required to produce the spatially-variable uplift of mapped palaeoshorelines onshore outcropping between Messina town and Taormina town^28,29,31,33^ (Fig. 1). Extension across the Messina Strait implied by GPS of ~2.5 mm/yr^34^, and the fault has been imaged offshore with geophysical data^9^.

The 28^th^ December 1908 Messina earthquake (Mw 7.1; 05:20.27 CET) affected the area, including the cities of Messina and Reggio Calabria, with devastating MCS intensities up to XI^3,35,36^. Eleven historic seismograms are available, but the spatial distribution and azimuthal coverage limit the seismological information that can be derived^3,11,37,38^. Investigations on earthquake environmental effects (EEE) have been carried out^3^; these included searches for ground ruptures, ground shaking, liquefaction, coastal retreat, gas emissions, slope movements both on land and submarine, acoustic and light effects and hydrological anomalies; although many environmental effects were noted, no clear surface ruptures were identified.

With the lack of a clear surface rupture, the main indications of primary co-seismic effects are ground elevation changes in Sicily and Calabria (Fig. 3a). A tsunami inundated both sides of the Messina Strait with run-up reaching 13 m in place^3^. We note that it has been suggested that many of the levelling benchmarks were reached by the tsunami^25^, raising concerns that some may have been disturbed. For example, contemporary coastal slumping is reported in the port of Messina (Fig. 1, inset a; see ref. ^12^ for a further details). Also, the mountains along the Sicilian coast were affected by landsliding^39,40^ (Fig. 1), raising concerns about disturbance of levelling sites on these steep mountainous slopes. However, it has been pointed out that, in general, there is “good coherence amongst the data”^3^, an inference that is supported by the systematic changes in uplift/subsidence with distance (Fig. 3a). We interpret this coherence, as do others^11,13–18^, to mean that a primary signal of the co-seismic motions produced by the earthquake survives in the data. Moreover, the severe tsunami effects have been related to co-seismic displacement of the sea floor in the Messina offshore^3^; this evidence might explain why the extensive surface fault ruptures expected on land for such a large magnitude event have not been described in the literature.

## Modelling Results

**Figure 4:**
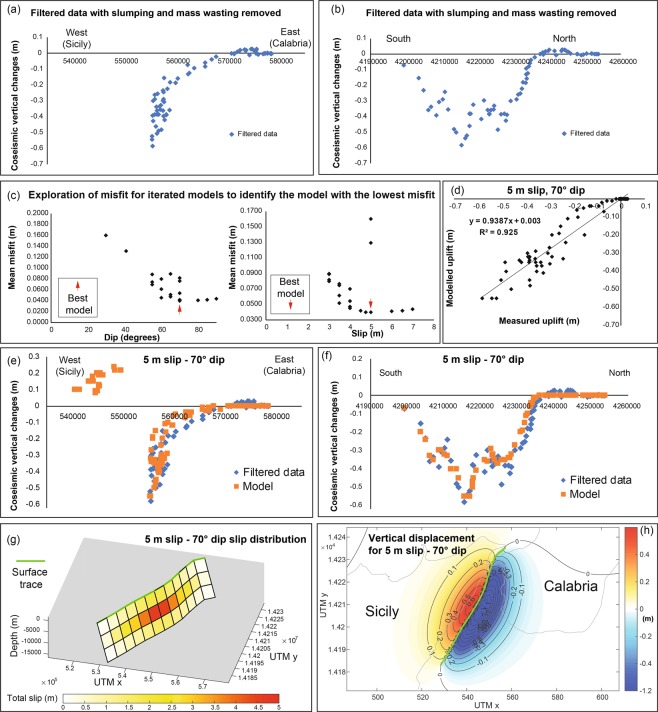
Results showing our preferred fault model which gives the lowest misfit to the filtered levelling data. In (**a**) an E-W plot of the co-seismic elevation changes from the filtered locations is shown. In (**b**) a N-S plot of the co-seismic elevation changes from the filtered locations is shown. In (**c**) well-plots show the best model which minimizes the absolute misfit modelling the dip angle and the max slip for the Messina Strait Fault. In (**d**) a linear regression analysis with R^2^ value > 0.9 is shown between the measured but filtered co-seismic elevation changes and the modelled elevations derived from our preferred model. In (**e**) an E-W plot is shown with our preferred model with modelled vertical changes (orange colour) and the measured but filtered vertical changes (blue colour) by Loperfido, (1909). In (**f**) a N-S plot is shown with our preferred model with modelled vertical changes (orange colour) and the measured but filtered vertical changes (blue colour) by Loperfido, (1909). In (**g**) a 3D view of the modelled seismogenic source (The Messina Strait Fault) with the associated slip distribution in depth is shown. In (**h**) co-seismic uplift/subsidence contours produced by our preferred modelled fault in the half-elastic space are shown.

The profile of the co-seismic vertical deformation motions compared to other well-mapped normal faulting surface ruptures in Figs 3a and 4a suggests, even before modelling, that an east-dipping fault is likely to be responsible for the earthquake. There are some candidate faults of this kind (Fig. 1), such as (a) the onshore mapped fault that dips east (the Messina Fault, MF), located ~4 km west of Messina, that separates Palaeozoic basement metamorphic rocks from Miocene-recent sediments^7,26^, and (b) the offshore Messina-Taormina Fault (MTF) that is suggested to be an active normal fault as it is thought to deform Late Quaternary marine terraces and Holocene coastal notches^29–31,33^. There is no field evidence of recent co-seismic displacement on the onshore fault scarp of the east-dipping “Messina Fault” (“MF” in Fig. 1), west of Messina town^3^; however, for completeness, we first attempted models that ruptured this ~25 km long onshore mapped fault. We were unable to reproduce the location and magnitude of the deformation (see Electronic Supplementary Materials ESM1 and ESM2 which detail the iterations performed). We then modelled the 58 km long offshore Messina-Taormina Fault described in the literature^27–29,31^. In particular, we iterated both the dip and amount of slip on this fault starting from values of 55° and 3 meters (see Fig. 4c). We found that although co-seismic tectonic subsidence was essentially in the correct location, 3 m of slip was unable to produce enough subsidence for fault dip values incrementally increased between 55° and 70°. We then increased the slip incrementally up to 7 m (with intervals of 0.5 m), trying fault dip values between 55° and 70°, for each slip magnitude. We also tried to model “low-dipping angle” faults with dip angle <45° (Fig. 4c) but we were not able to replicate the co-seismic deformation, obtaining higher misfit values (Fig. 4c). We also tried steeper dips up to 90°. Figure 4c shows the model that minimizes the misfit between measured and modelled uplift values is for a 70° dip and 5 m dip-slip, with surface slip of 50 cm, suggesting that the Messina-Taormina Fault is the capable fault that ruptured in the earthquake, with rupture on the sea-bed (models with higher misfits are shown in ESM1 and ESM2). Note that the absolute misfit represents the mean difference between the measured elevations from levelling data^4^, and the modelled elevations for each considered model; linear regression between these two datasets also describes the robustness of this correlation with the R^2^ value > 0.9 (Fig. 4d for our best model and Electronic Supplementary ESM1 and ESM2). Our best-fit model implies a magnitude of Mw 7.04, close to the well-accepted magnitude of Mw 7.1 proposed for the 1908 Messina earthquake^18^. We also iterated the rake, for the 70° and 5 m slip model, from −95° to −135° to investigate whether dextral slip was involved, bearing in mind that the error on the original vertical measurements is stated to be ±0.005 m^18^. Although the mean misfit for a rake of −105° was lower than that for −90° by 0.002 m, this is smaller than the uncertainty of the measurements, so the results are indistinguishable. Thus, the effect of changing the rake appears not to be resolvable and, although we have not excluded a minor dextral slip component, we report the −90° rake results as our preferred model (see ESM1). Therefore, we found that including dextral slip does not significantly improve our solution. It may be possible to improve our dip-slip model by using a more sophisticated slip-distribution (e.g. compare with the relatively simple slip distribution for our best fit model in Fig. 4g). However, as slumping of the coast and mass wasting on steep slopes^38,39^ may degrade the dataset, it is perhaps doubtful whether a more sophisticated model is warranted. Note that our proposed slip at depth model of 5 m provides an explanation for the highest co-seismic subsidence recorded in Reggio Calabria town (Figs 3 and 4), previously used^15,17^ as a constraint to propose more sophisticated slip models.

In summary, we show for the first time that the herein named Messina-Taormina Fault (formerly the “Taormina Fault”), already considered to be an active normal fault^29–31,33^, and partially accommodating the NW-SE-oriented GPS-based ~2.5 mm/yr crustal extension^34^ affecting the Messina Strait, is likely to be the source for the most destructive earthquake recorded in Europe in the 20^th^ and 21^st^ centuries.

## Discussion

We show that the 1907–1909 data resemble levelling data from other normal faulting earthquakes if projected onto an east-west transect (Fig. 3) instead of being plotted by levelling location number^11,18^. We have identified a known east-dipping fault^29–31,33^ in an offshore location as the likely seismic source, with a 70° dip, and 5 m slip with slip reaching the surface on the sea bed. (Fig. 4); this is consistent with the lack of contemporary reports of surface ruptures onshore.

Several previous models have attempted to resolve the long-lasting debate about which seismogenic source could have produced the 1908 Messina Earthquake^11,13–18^ (Fig. 2). It is understandable why early models did not utilise mapped faults as the fault map has evolved through time, particularly in the last few decades^41^; our modelling did utilise mapped faults. We emphasize that valuable information is contained within historical reports and we hope that our findings give fresh impetus to studies of historical accounts of past earthquakes. However, we also emphasize that insights into such historical data may be facilitated by recognizing the importance of clear geological and geomorphic faulted offsets in a region rather than proposing fault models that do not have a clear geological or geomorphic expressions^11,13–17^ (Fig. 2). It is important to note that we have not excluded that more complex slip distributions, or models with multiple closely-spaced ruptures^42,43^, may produce lower values of misfit between modelled and measured co-seismic movements, but this is beyond the purpose of this paper. We also stress that several studies show that it is an accepted approach to use a simple “single-fault model” to depict fault sources for normal faulting-related damaging earthquakes such as the 1983 Borah Peak Earthquake (Mw 7)^21^, the 2006 Mozambique Earthquake (Mw 7)^44^, the 2008 Dangxiong Earthquake in the southern Tibetan Plateau (Mw 6.3)^45^, the 2008 Nima Earthquake in Tibet (Mw 6.4)^46^ and the 2009 L’Aquila Earthquake in Italy (Mw 6.3)^47^. Indeed, it is arguably a simpler scientific scenario to propose known mapped capable faults as potential earthquake sources than proposing previously-unknown and unmapped seismogenic sources. In this case, it was simply a matter of re-plotting the data to show variation in vertical motions with distance across the strike of the mapped Quaternary active and capable faults that produced the new insight (Fig. 3). Lastly, we stress that our preferred fault source model for the 1908 Messina Earthquake shown in this paper could provide new input parameters for tsunami modellers, trying to gain new insights about another long and highly-debated issue regarding the tsunami that occurred after the 1908 earthquake. Indeed, there is no agreement about the cause of the tsunami; some authors propose a prominent submarine landslide as a cause of the tsunami^48,49^, while others rule this out^50^. An alternative hypothesis suggests a composite cause, with a co-seismic seafloor displacement alongside a prominent submarine landslide within the Messina Strait^51^.

## Method

We have re-plotted levelling data from the 1907–1909 survey (the data table is from ref.^18^, re-presenting the original data from ref.^4^) projected onto an E-W line, quasi perpendicular to the ~NNE-SSW strike of the Quaternary normal faults around the Messina Straits. We chose an E-W transect because the Quaternary faults have curvilinear traces and we have *no a priori* knowledge of which one or which part of them ruptured. Thus, an E-W transect was chosen to study the simplest question of whether the fault dips generally to the east or west. Figure 3a shows a plot of the levelling data on a E-W oriented transect. There is a clear signal of subsidence for the Calabrian sites. Benchmarks from Sicily show subsidence, with the exception of 4 locations with very minor uplift (<0.007 m), whose magnitude is, in any case, less than the error on the measurements, so we cannot rule out subsidence for these as well. The lack of clear uplift is unusual for the footwall of a normal faulting earthquake^21,44–46,52^. For this reason, we have questioned whether benchmarks located on the Sicilian side could have been affected by secondary processes. Indeed, previous studies^39^ including work by ISPRA (the Italian Institute for Environmental Protection and Research) show that the entire Sicilian side, where the highly-fractured and deformed Palaeozoic bedrock outcrops^39^, is affected by landslide processes of rock fall, and in places rotational/translational rock slides^39,40^. The footwall sites mentioned above are either affected by coastal slumps (Fig. 1d) or exist on slopes of 10–25° for which we suspect mass-wasting (see ESM3). We have therefore decided to filter out benchmarks on the Sicilian side to address concerns that they may have been disturbed by either mass wasting on steep slopes in the mountains west of Messina^39,40^, by slumping of the harbour within Messina^12^, or disturbance by the tsunami. This filtering removed data points in Sicily, and some from the extreme south of Calabria region where we suspect coastal disturbance (see Figs 1 and 4a, and ESM1 for data included and excluded from the modelling). We then input the curvilinear traces of the mapped normal faults into the Coulomb 3.4 software^53,54^, using a new Matlab code^23^. This code enables us to model vertical and horizontal displacements arising from earthquakes on faults with variable-strike geometry. It is well established that fault geometry influences Coulomb stress transfer^23,53,55^, and therefore will influence strain and displacements surrounding the causative fault. We iterated the location of the fault, the dip and dip direction of the fault, the amount of slip at depth and hence the amount of slip at the surface. We recorded the absolute misfit between measured and modelled uplift and subsidence for each of the 114 levelling locations from ref.^4^ for each model run. Our preferred model minimizes the misfit in the filtered sub-set of the data (Fig. 4). We also iterated the rake for the case of 5 m slip on the 70° dip of the MTF. In particular, our modelling concentrated on well-known and mapped active faults, in agreement with the geology and geomorphology characterizing the Messina Strait, in contrast to previous models. We show the results of all the model runs in Electronic Supplementary Materials ESM1 and ESM2, and our preferred model in Fig. 4.

## Conclusions

Re-examination of levelling data from 1907–1909 reveals that the Mw 7.1 1908 Messina earthquake ruptured a 70^o^ east-dipping normal fault with 5 m dip-slip at depth, and slip at the surface, 15 km down-dip width, with the surface rupture located offshore on a mapped Quaternary active fault, the Messina-Taormina Fault (former “Taormina Fault”). Our work should re-invigorate the drive to link mapped capable faults with historical earthquakes rather than ignoring the valuable insights that the geology and geomorphology can bring.

## Supplementary information

Supplementary Information

ESM1

ESM2 and 3

## Data Availability

All data generated during this study are included in the Supplementary Information files (ESM1, ESM2 and ESM3).
