# Supplementary material for: Slip on a mapped normal fault for the 28th December 1908 Messina earthquake (Mw 7.1) in Italy
Source: Sci Rep. 2019 Apr 24;9:6481. doi: 10.1038/s41598-019-42915-2 (PMC6482148; doi:10.1038/s41598-019-42915-2)
Supplement: Supplementary file 1 — Supplementary Information [file 41598_2019_42915_MOESM1_ESM.docx]

SUPPORTING INFORMATION FOR

**Slip on a mapped normal fault for the 28^th^ December 1908 Messina earthquake (Mw 7.1) in Italy**

Meschis, M.^1^*, Roberts, G. P.^1^, Mildon, Z. K.^2^, Robertson, J.^1^, Michetti, A. M.^3^, Faure Walker, J. P.^4^

1- Department of Earth and Planetary Sciences, Birkbeck, University of London, UK

2- School of Geography, Earth and Environmental Sciences, University of Plymouth, UK

3- Università degli Studi dell’Insubria, Como, Italy

4- Institute for Risk and Disaster Reduction, UCL, London, UK

*corresponding author marco.meschis.14@ucl.ac.uk

**Contents of submitted supplementary Information**

“ESM1.xcl”, “ESM2 and 3.pdf”

**Description**

In this section, we provide information about Electronic Supplementary Materials (ESM) alongside the first submission of this paper for a publication in *Scientific Reports.*

In particular, we provide two ESM file showing the data used to derive our results shown in Figure 4.

ESM1 shows the excel file where all our modelling for different dip angles and slip at depth is made. In particular, two sheets are presented: “sorted values” sheet shows the iteration between the filtered levelling data and the obtained elevations for different models for the “Messina-Taormina Fault” (MTF) and the “Messina Fault” (MT); “Rake vs Misfit” sheet shows the results of modelling the different rake angles to test the hypothesis of dextral coseismic movement, starting from our preferred model of 70^o^ and 5 m slip.

ESM2 shows a pdf file where the x-y axes are fixed and the modelled elevations (dark red color) for each model are shown against the measured levelling data (blue color). Furthermore, for each model the value of the related misfit is shown.

ESM3 shows a figure where our preferred model is shown, including the 4 filtered out benchmarks on the footwall of the proposed MTF presenting very minor uplift. For these locations (1) the values of uplift are smaller than the errors on the measurements, so we doubt whether the sites have indeed been uplifted, and (2) the locations are on steep slopes, so we suspect mass movement.

*To whom correspondence should be addressed. E-mail: marco.meschis.14@ucl.ac.uk
