# Supplementary material for: Slip on a mapped normal fault for the 28th December 1908 Messina earthquake (Mw 7.1) in Italy
Source: Sci Rep. 2019 Apr 24;9:6481. doi: 10.1038/s41598-019-42915-2 (PMC6482148; doi:10.1038/s41598-019-42915-2)
Supplement: Supplementary file 3 — ESM2 and 3 [file 41598_2019_42915_MOESM3_ESM.pdf]

Slip on a mapped normal fault for the 28th December 1908 Messina earthquake (Mw 7.1) in Italy.

Meschis, M.[1*], Roberts, G. P.[1], Mildon, Z. K.[2], Robertson, J.[1], Michetti, A. M.[3], Faure Walker, J. P.[4]

Electronic Supplementary Material 2 (ESM2)

1- Department of Earth and Planetary Sciences, Birkbeck, University of London, UK
2- School of Geography, Earth and Environmental Sciences, University of Plymouth, UK
3- Università degli Studi dell'Insubria, Como, Italy
4- Institute for Risk and Disaster Reduction, UCL, London, UK
*corresponding author marco.meschis.14@ucl.ac.uk

Well plots demonstrate that a 70º dip and 5 m slip is the best model

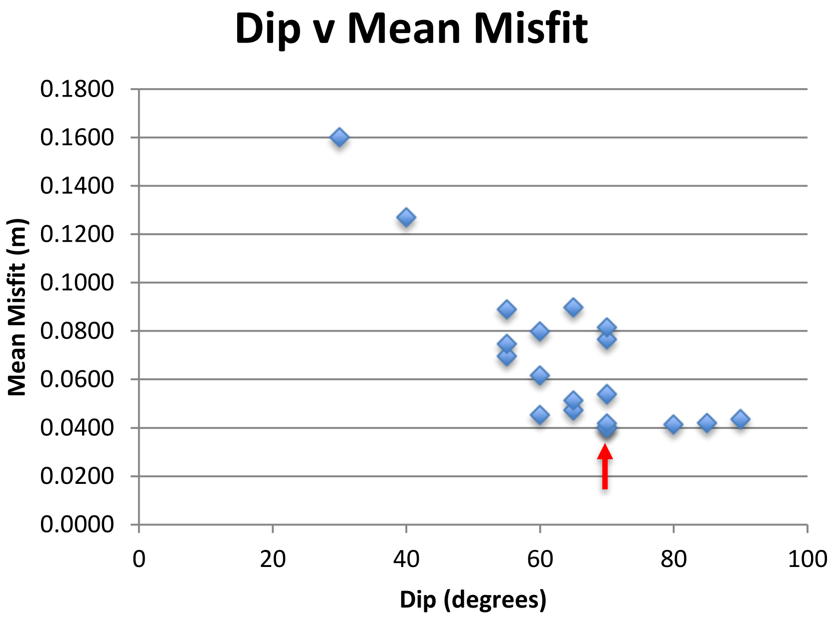

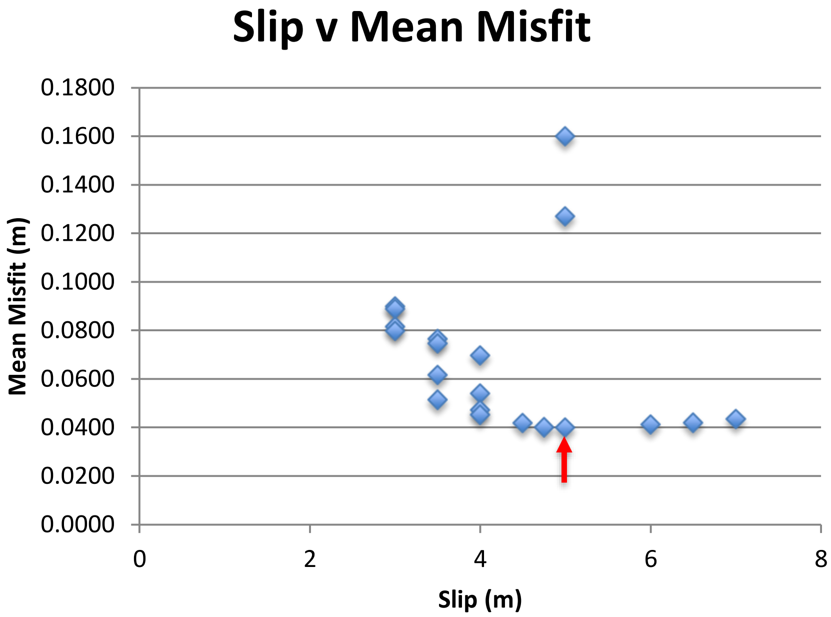

↑ Our preferred model

East – West plots

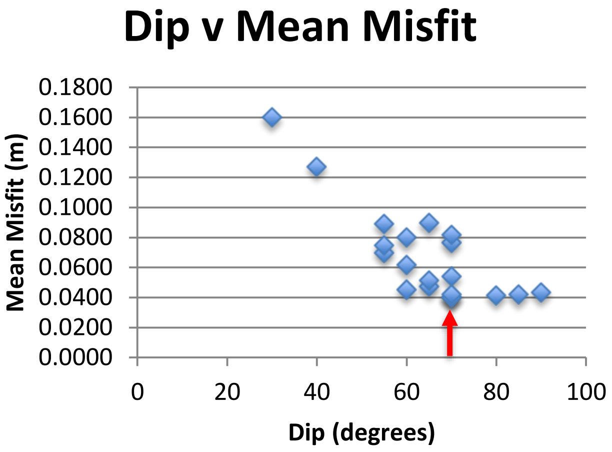

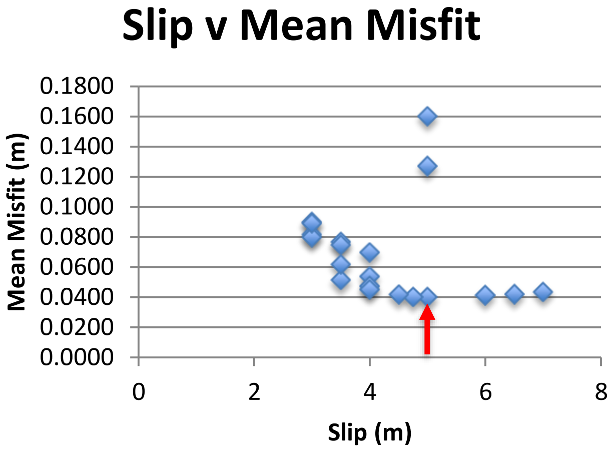

West
(Sicily)

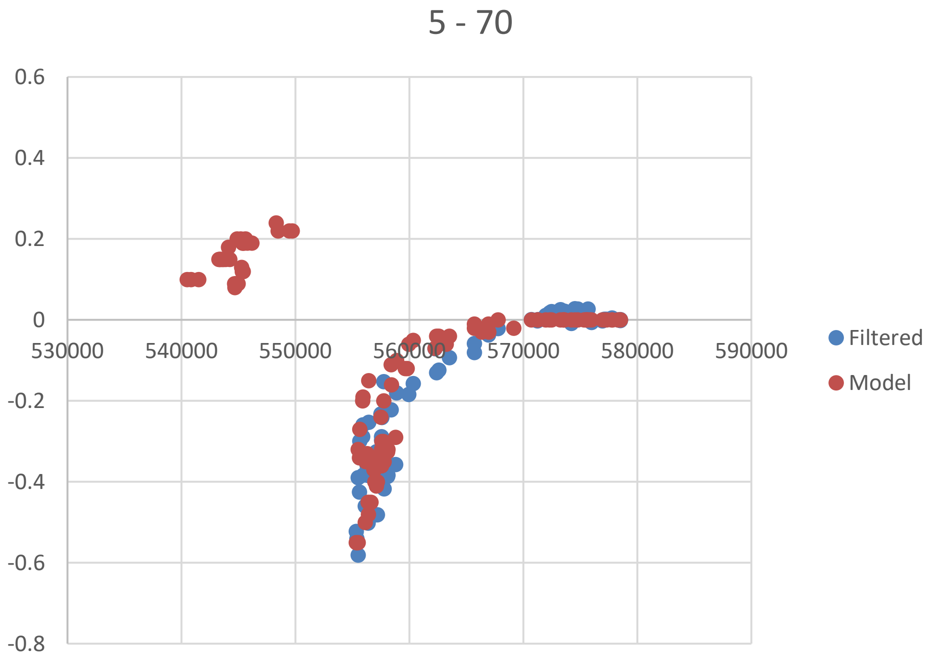

East
(Calabria)

Slip = 5 m; Dip = 70 degrees

Best fit model!!!

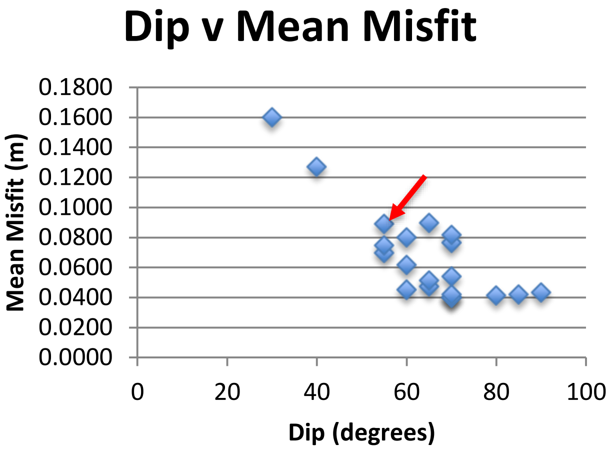

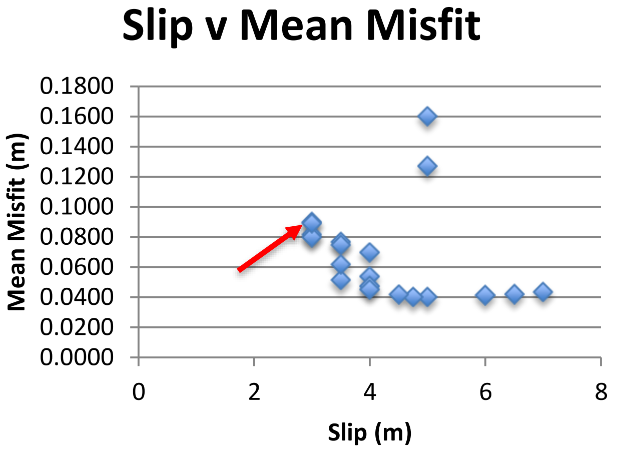

West
(Sicily)

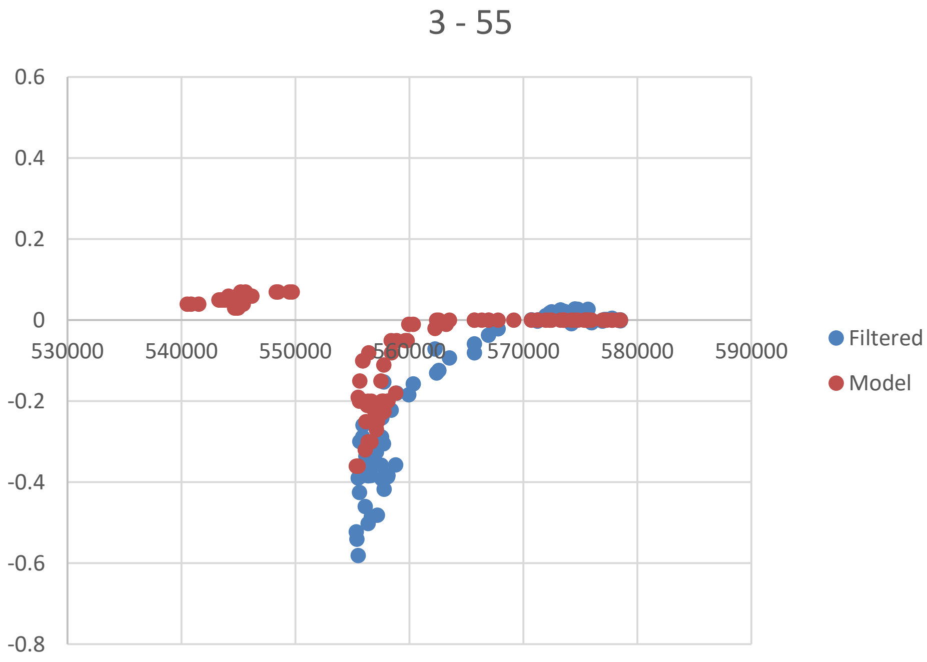

East
(Calabria)

Slip = 3 m; Dip = 55 degrees

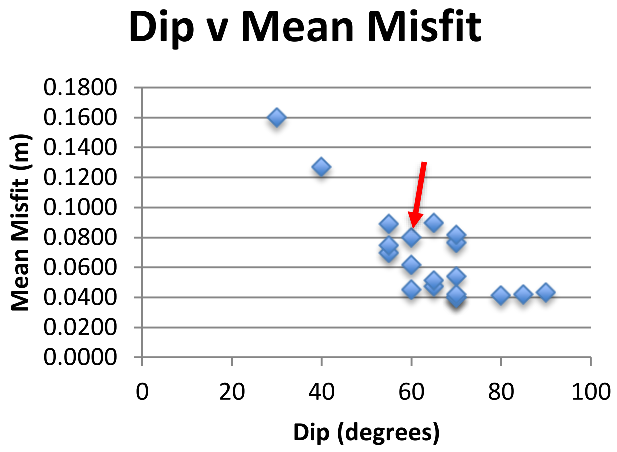

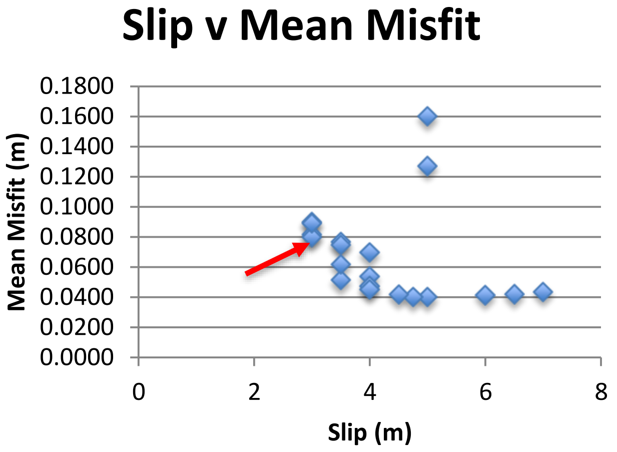

West
(Sicily)

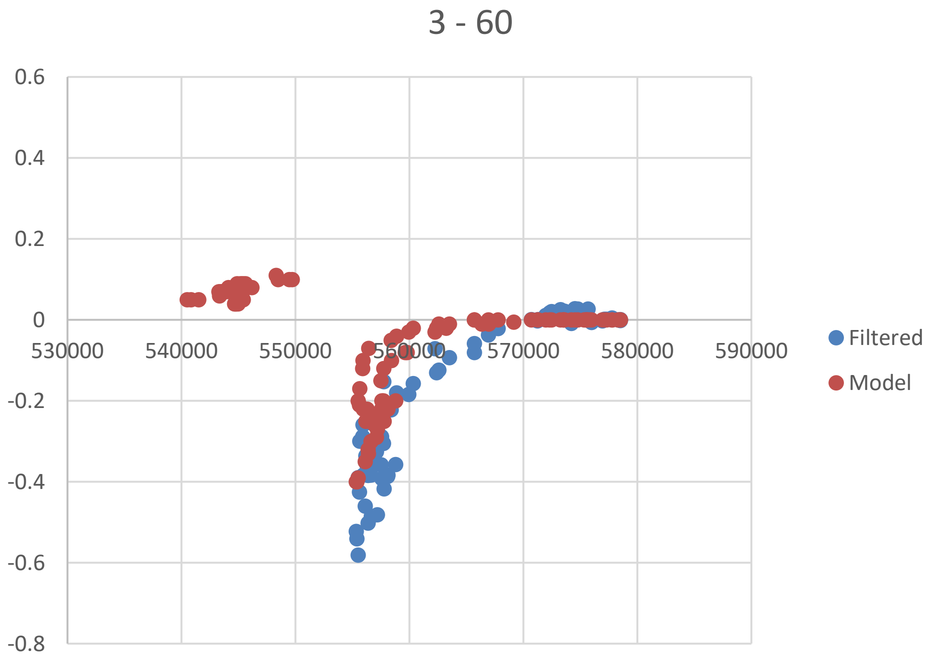

East
(Calabria)

Slip = 3 m; Dip = 60 degrees

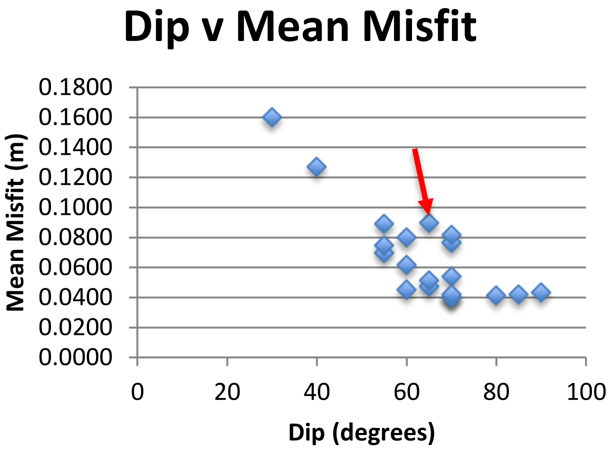

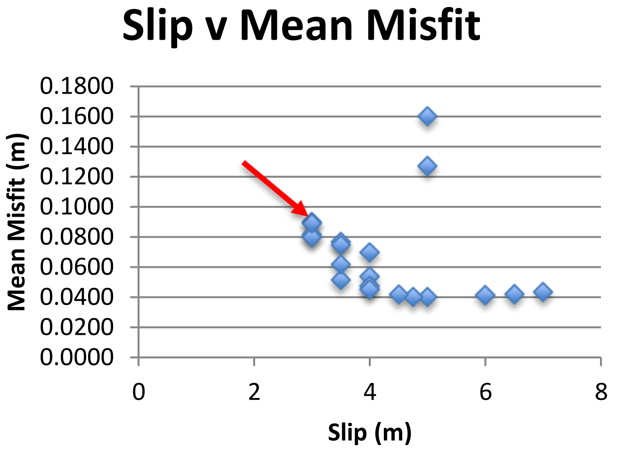

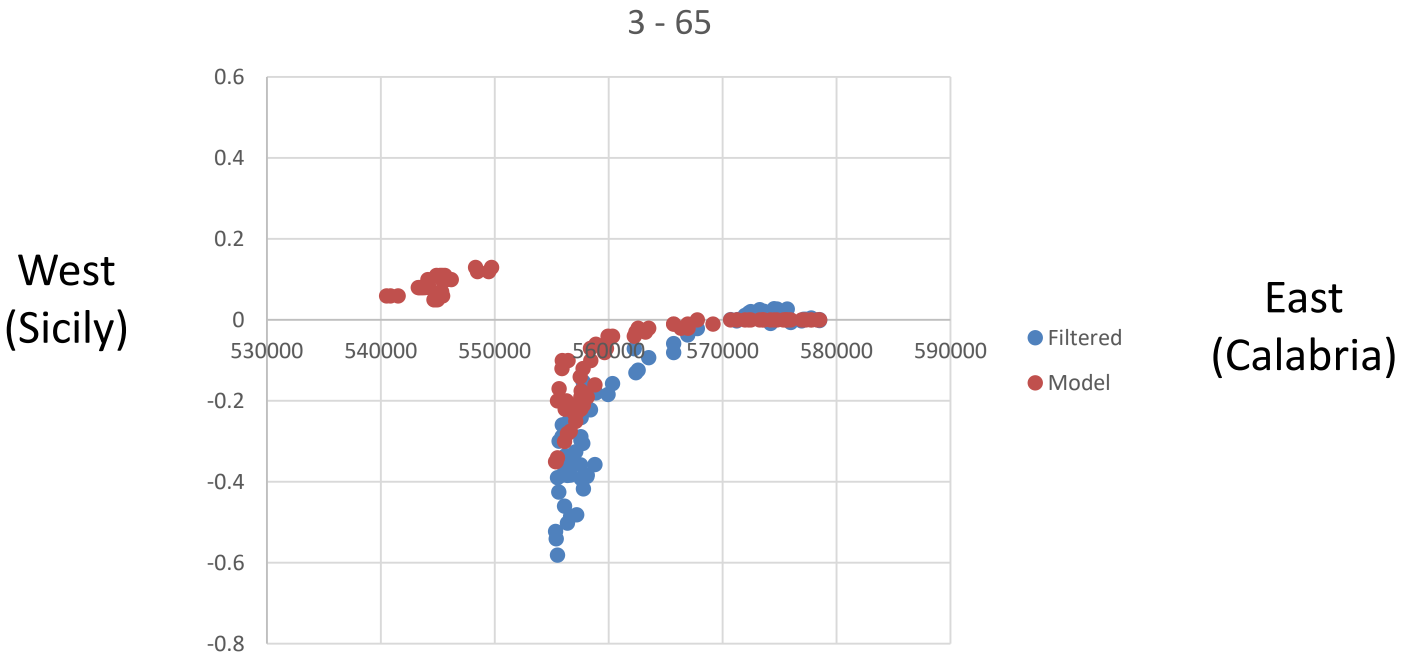

Slip = 3 m; Dip = 65 degrees

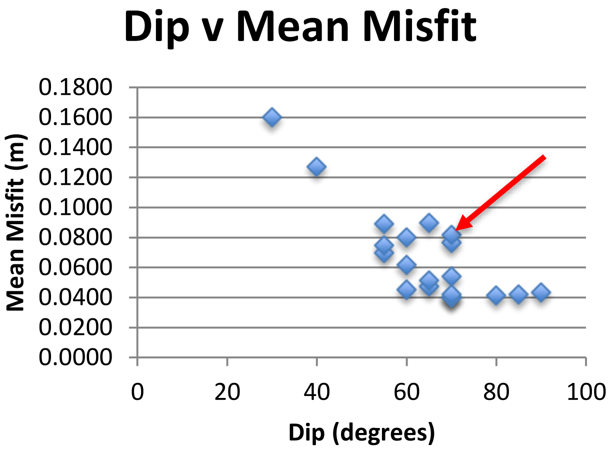

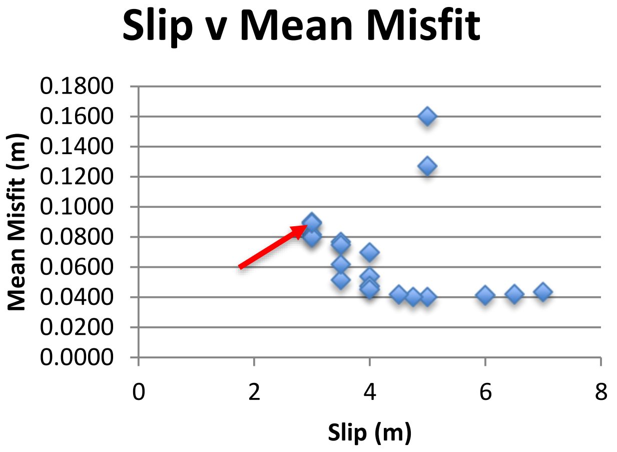

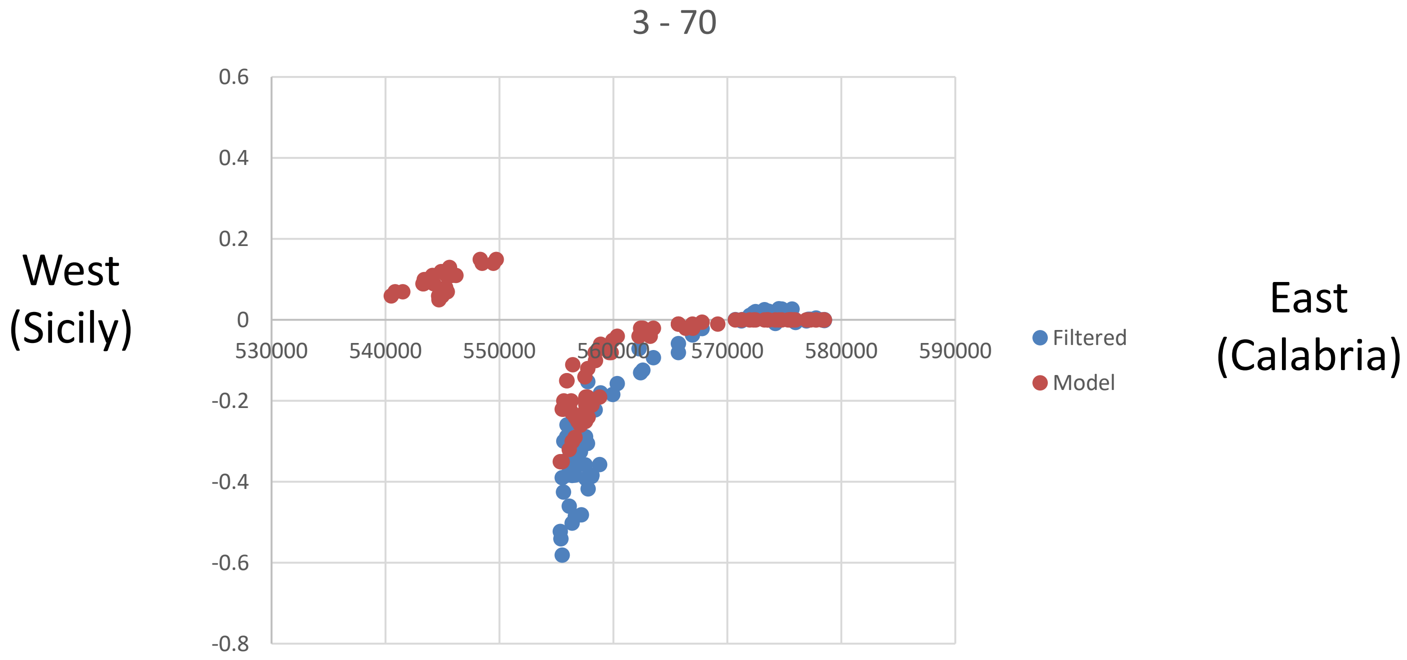

Slip = 3 m; Dip = 70 degrees

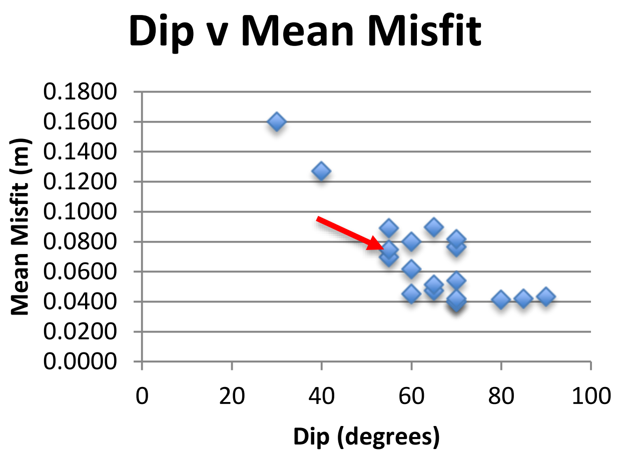

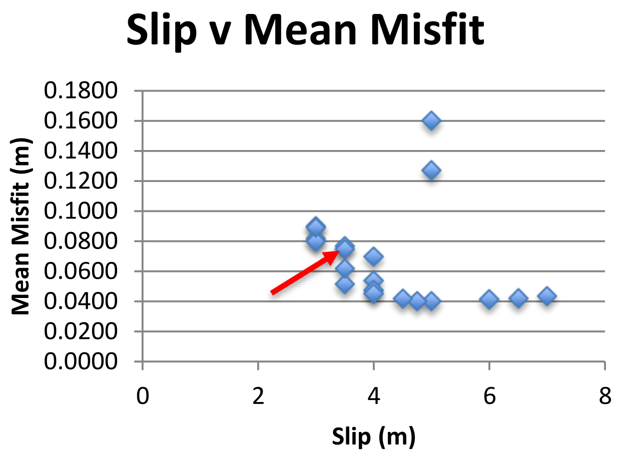

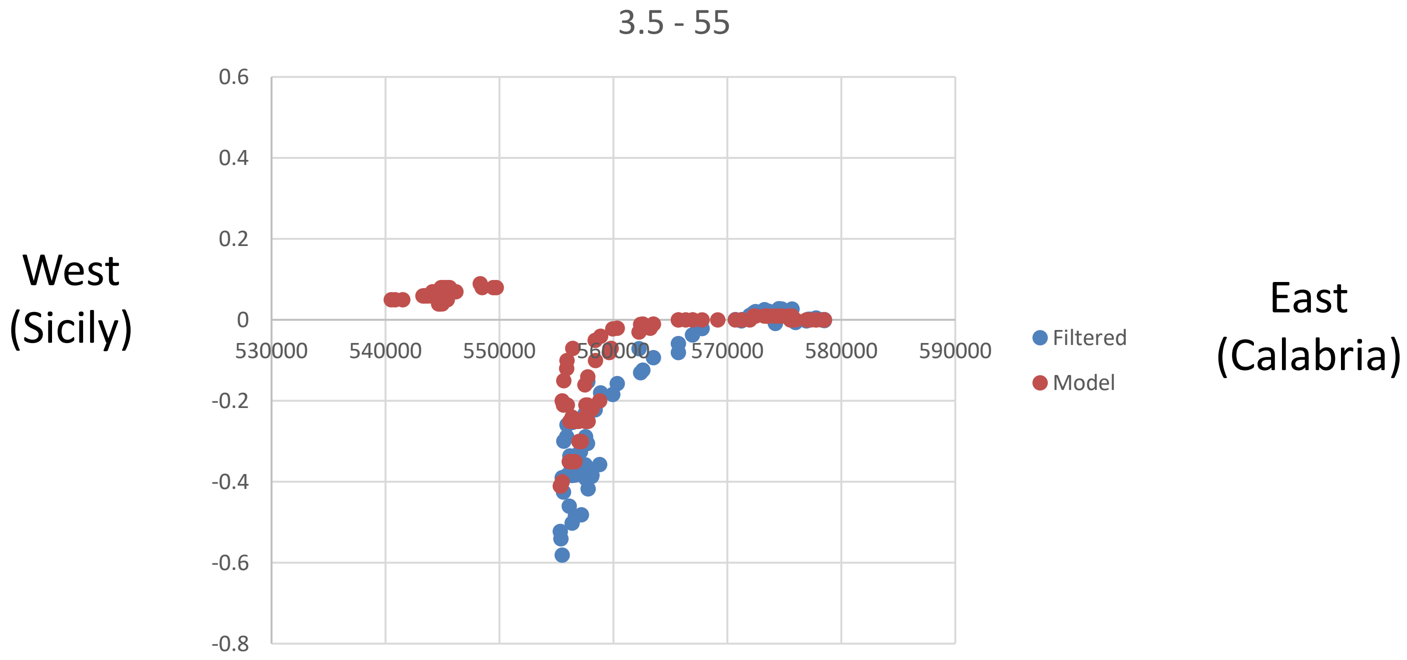

Slip = 3.5 m; Dip = 55 degrees

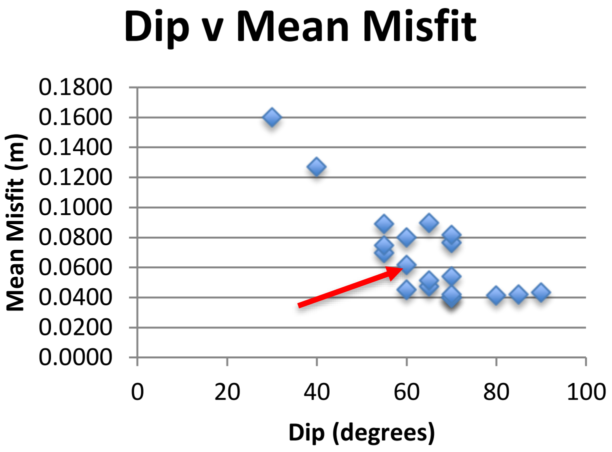

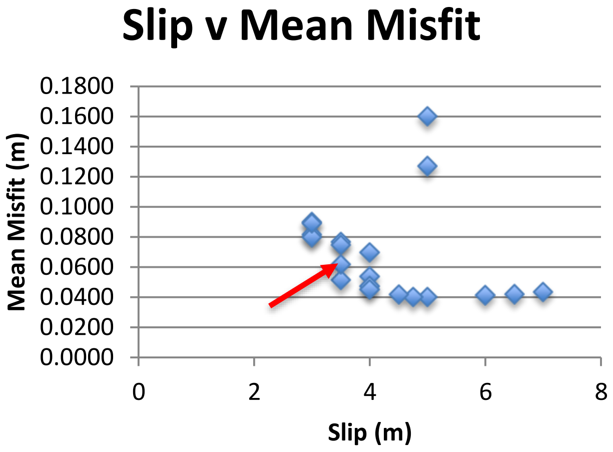

West
(Sicily)

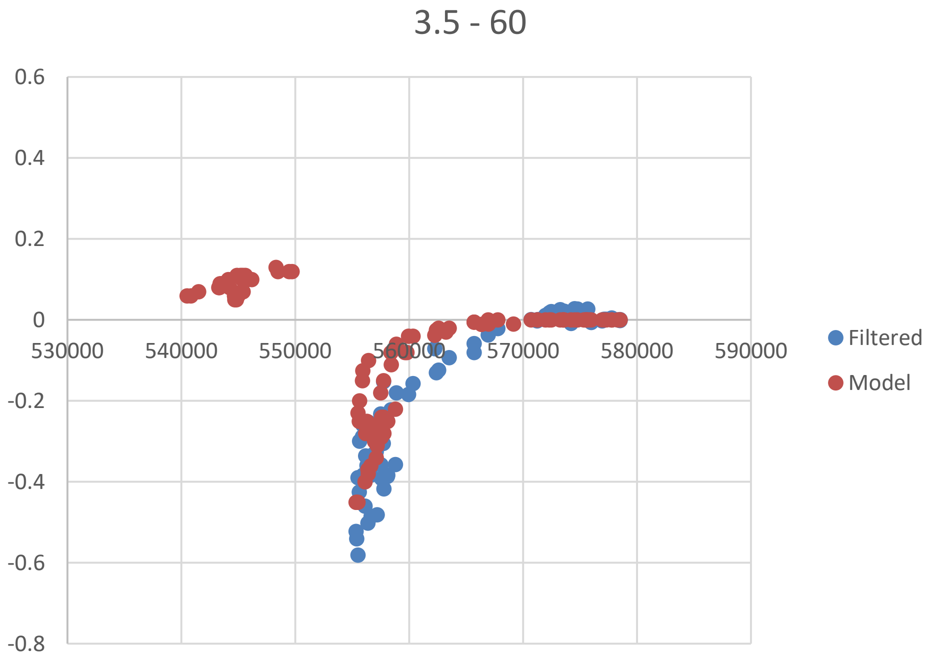

East
(Calabria)

Slip = 3.5 m; Dip = 60 degrees

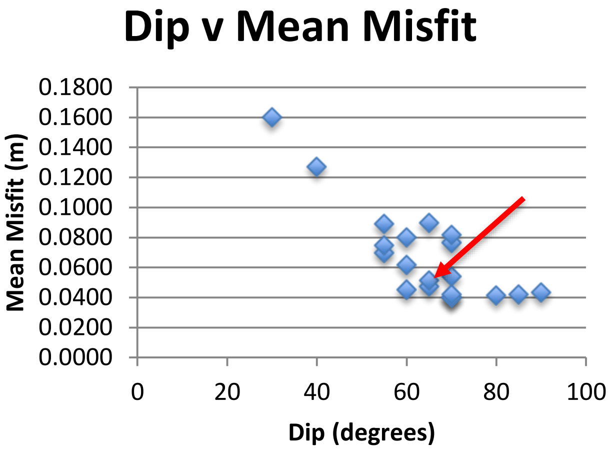

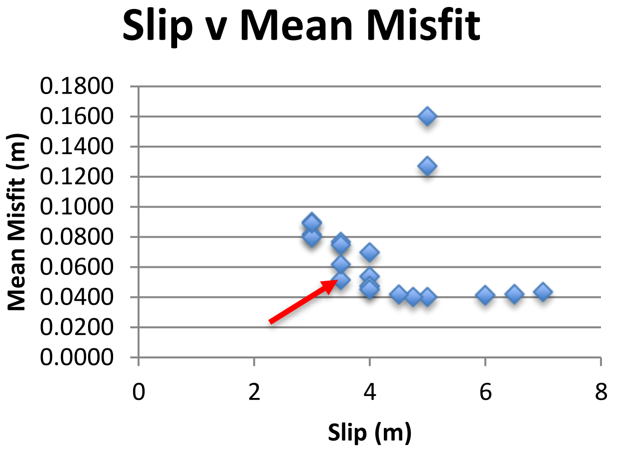

West (Sicily)

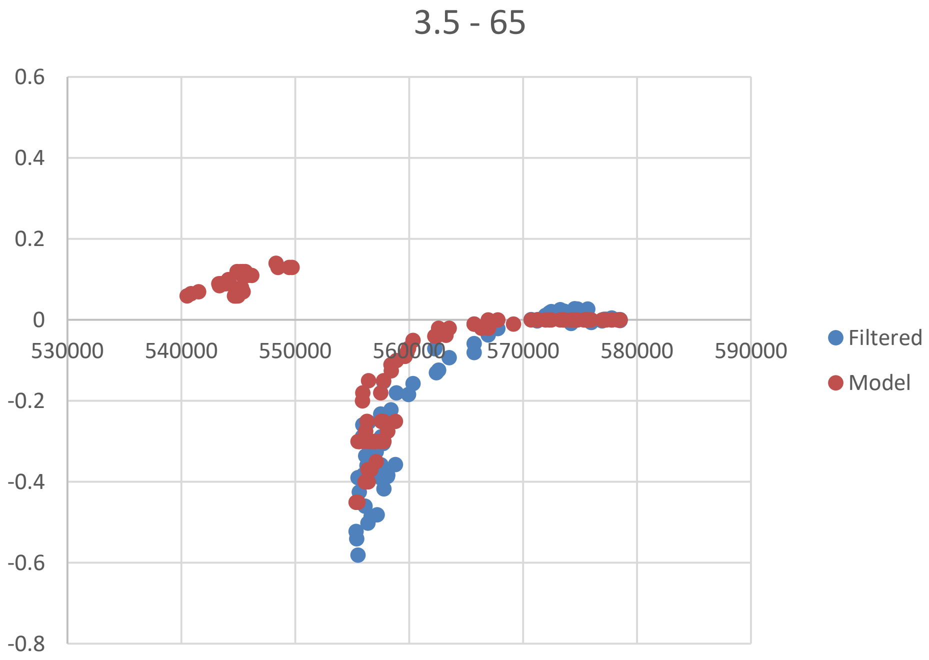

East (Calabria)

Slip = 3.5 m; Dip = 65 degrees

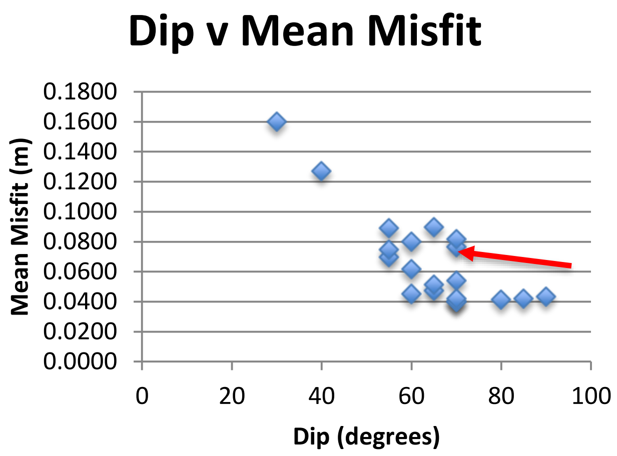

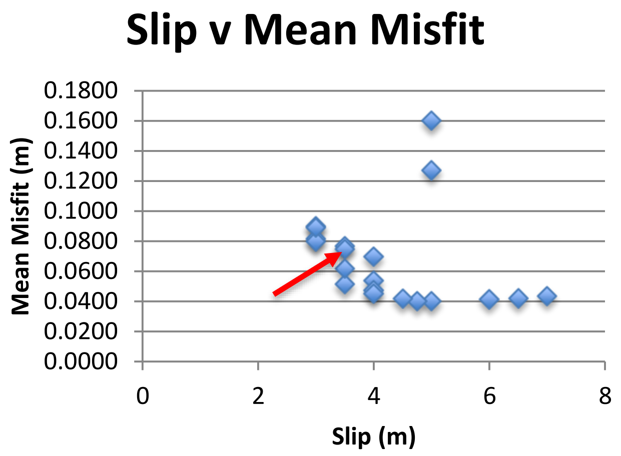

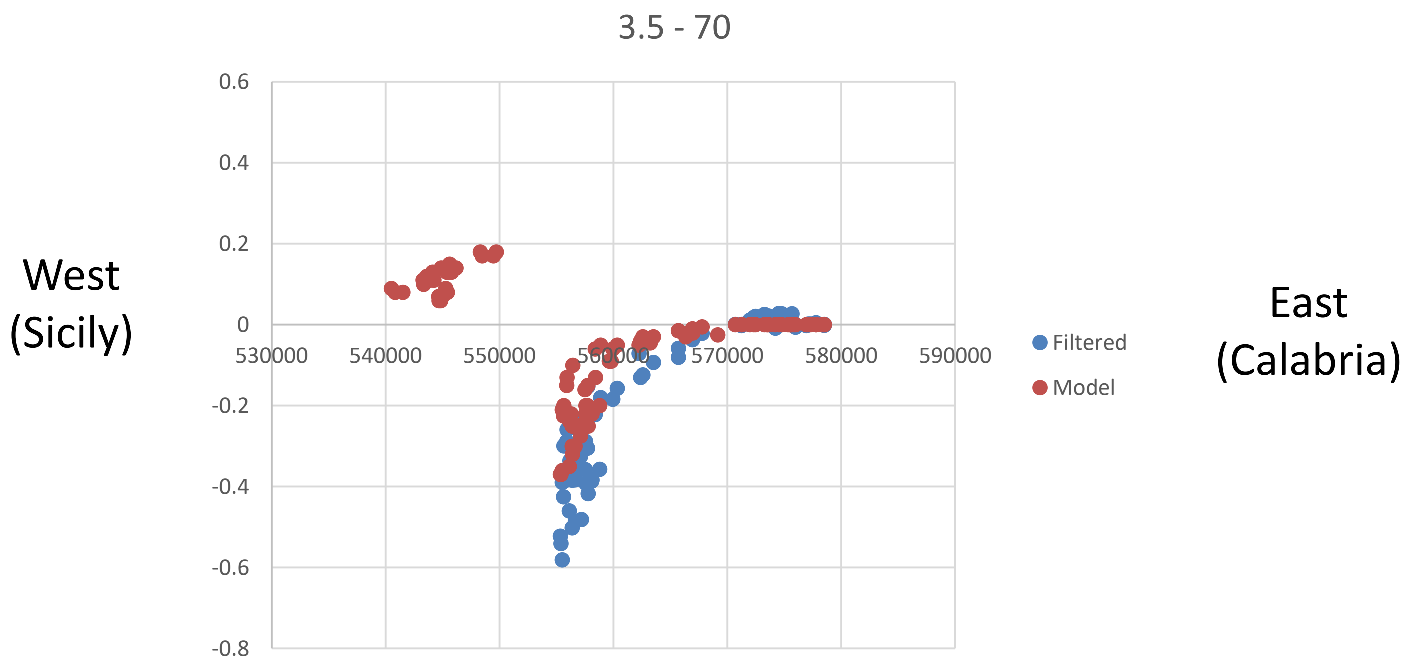

Slip = 3.5 m; Dip = 70 degrees

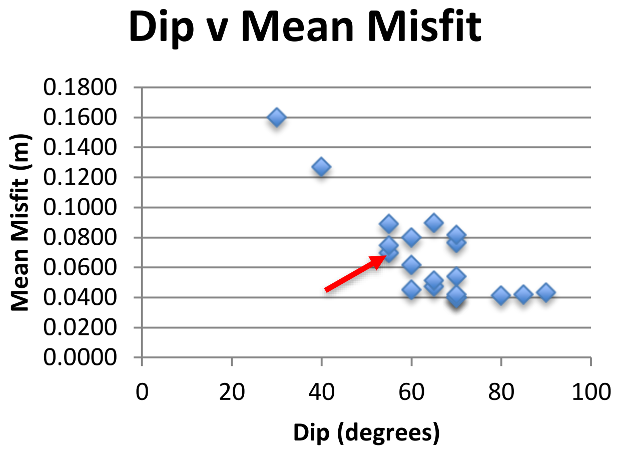

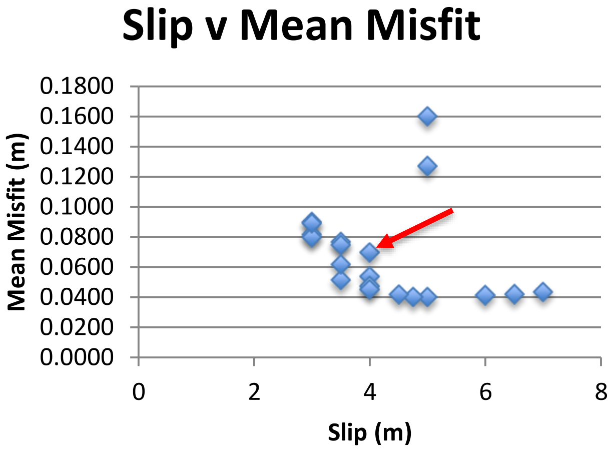

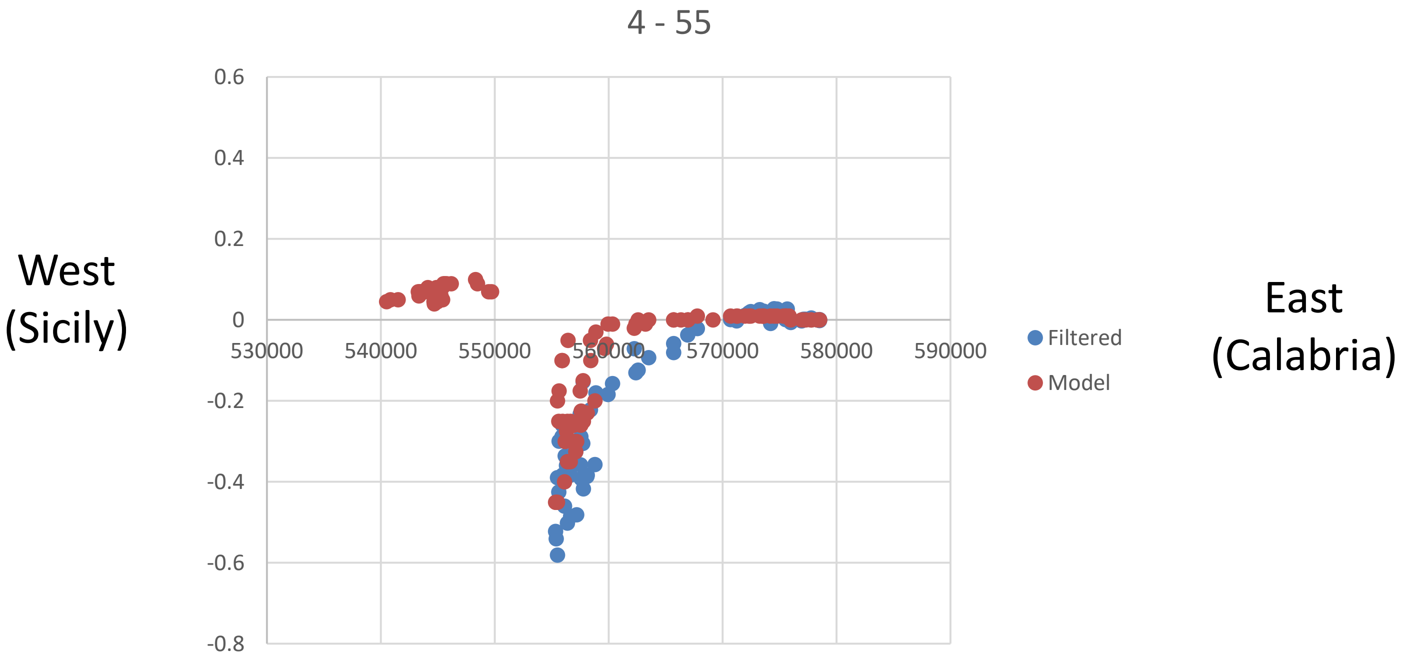

Slip = 4 m; Dip = 55 degrees

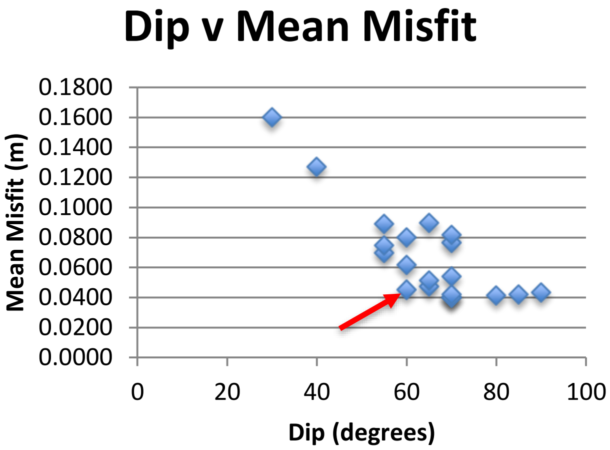

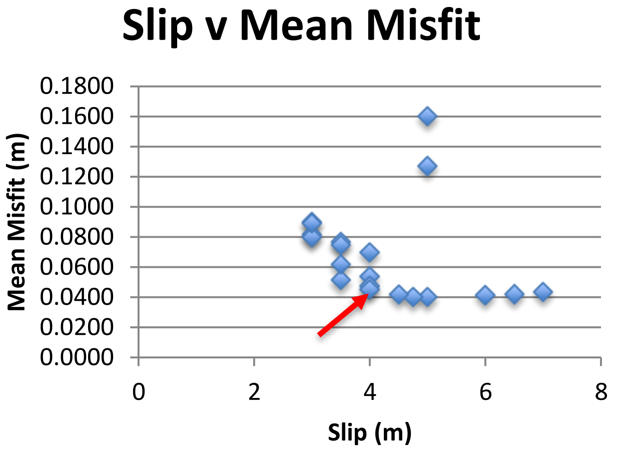

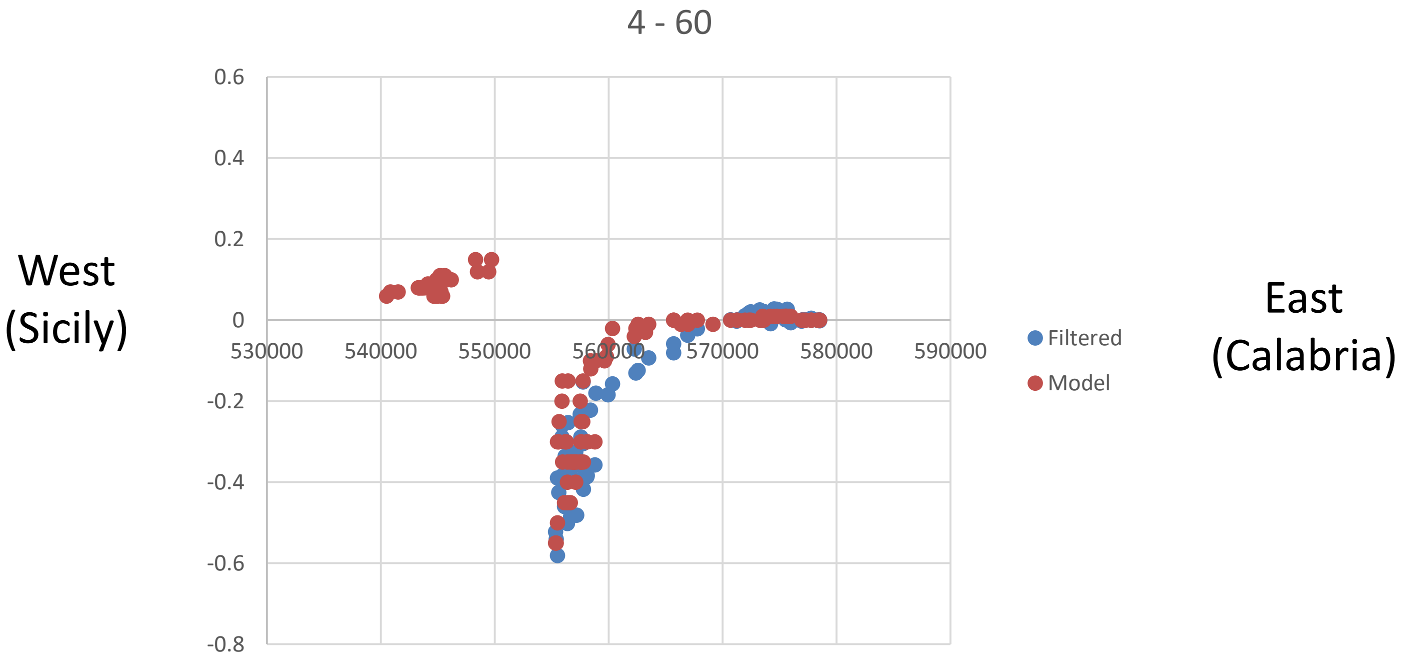

Slip = 4 m; Dip = 60 degrees

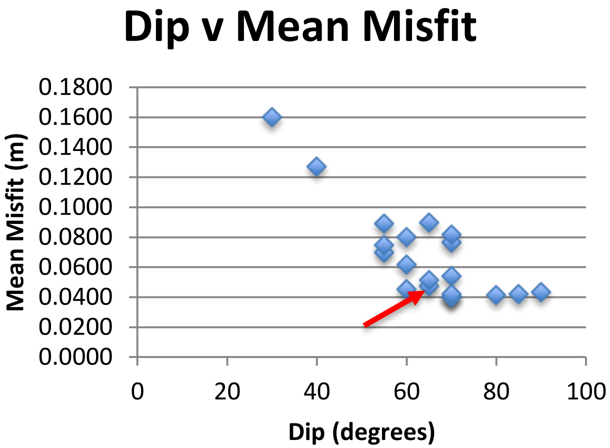

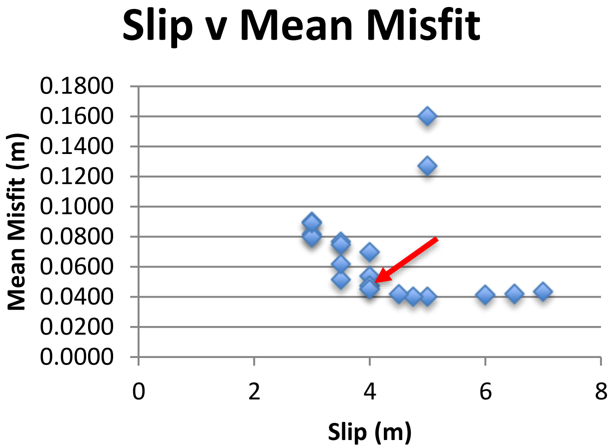

West
(Sicily)

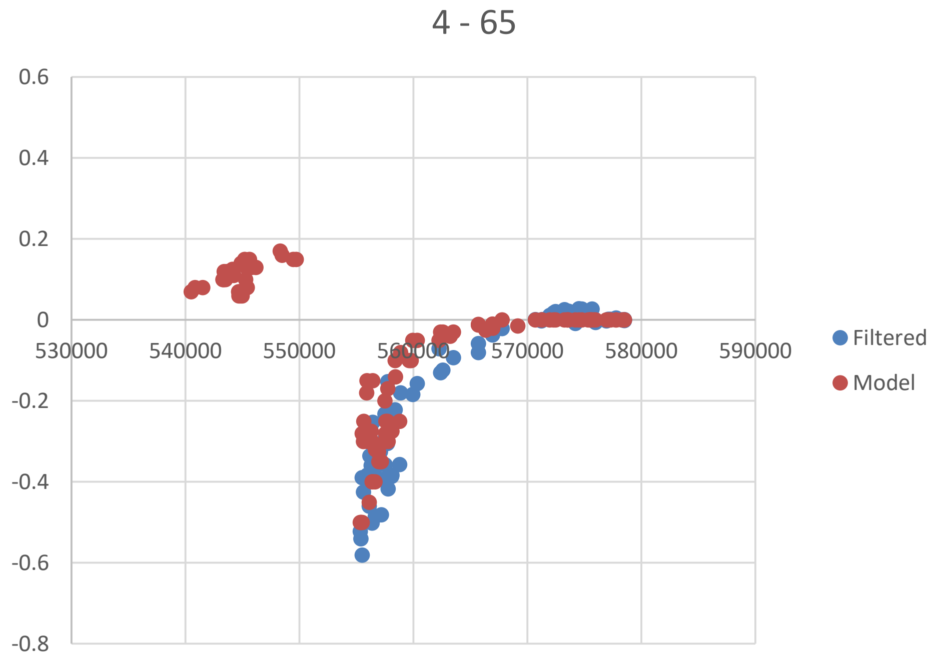

East
(Calabria)

Slip = 4 m; Dip = 65 degrees

Dip v Mean Misfit

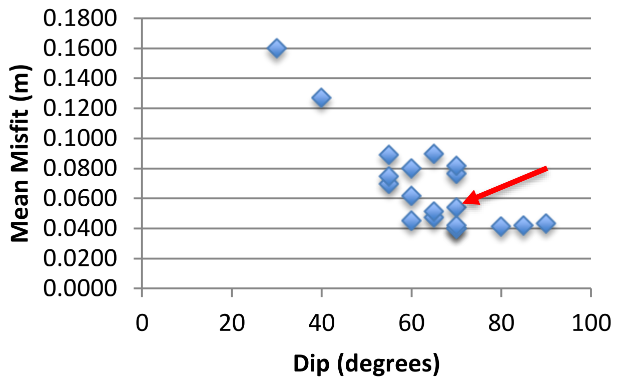

Slip v Mean Misfit

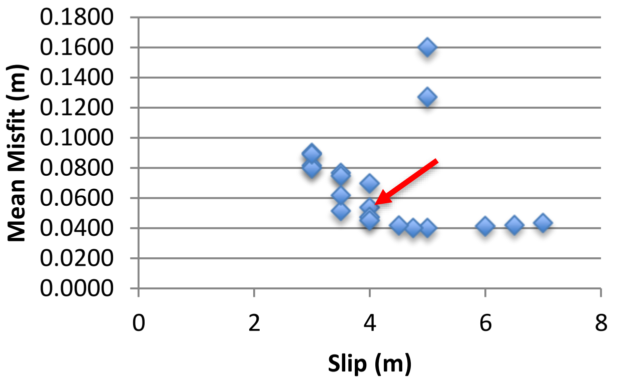

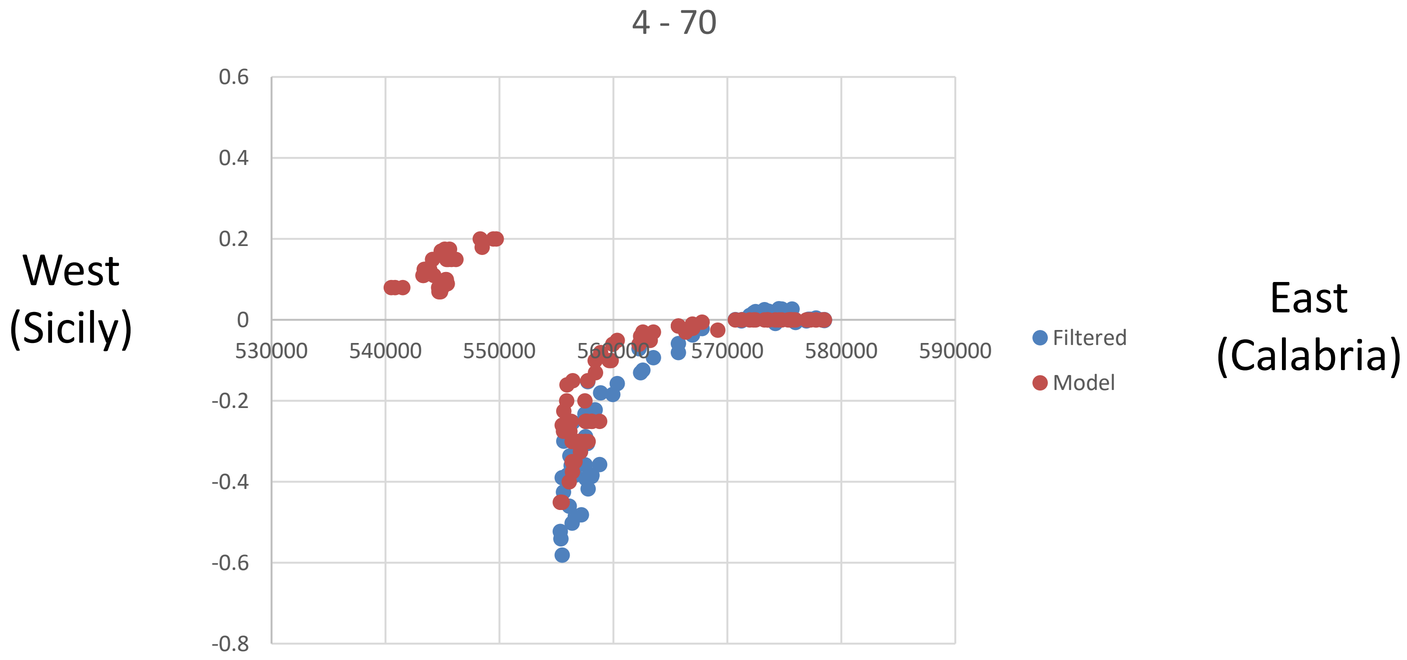

Slip = 4 m; Dip = 70 degrees

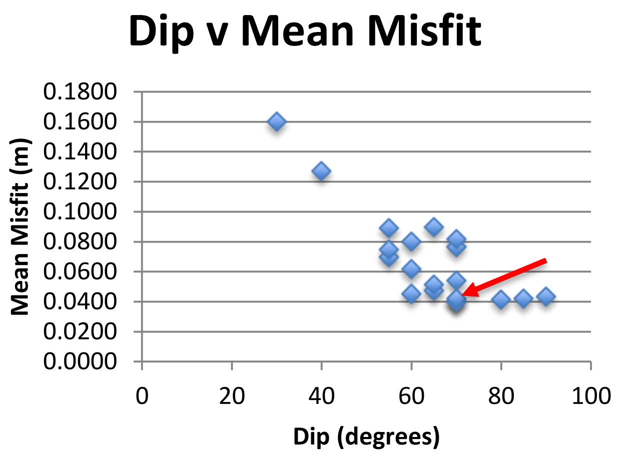

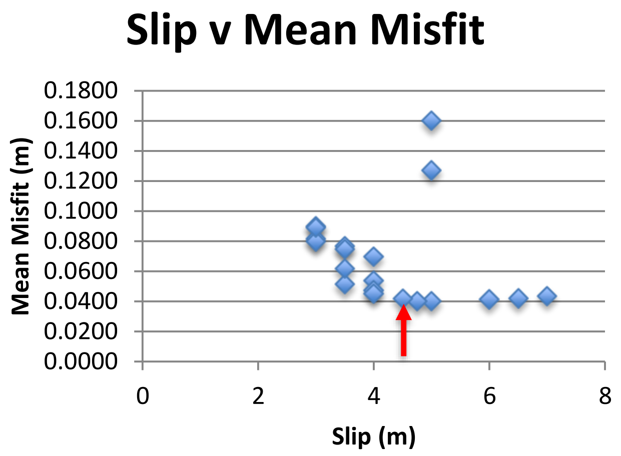

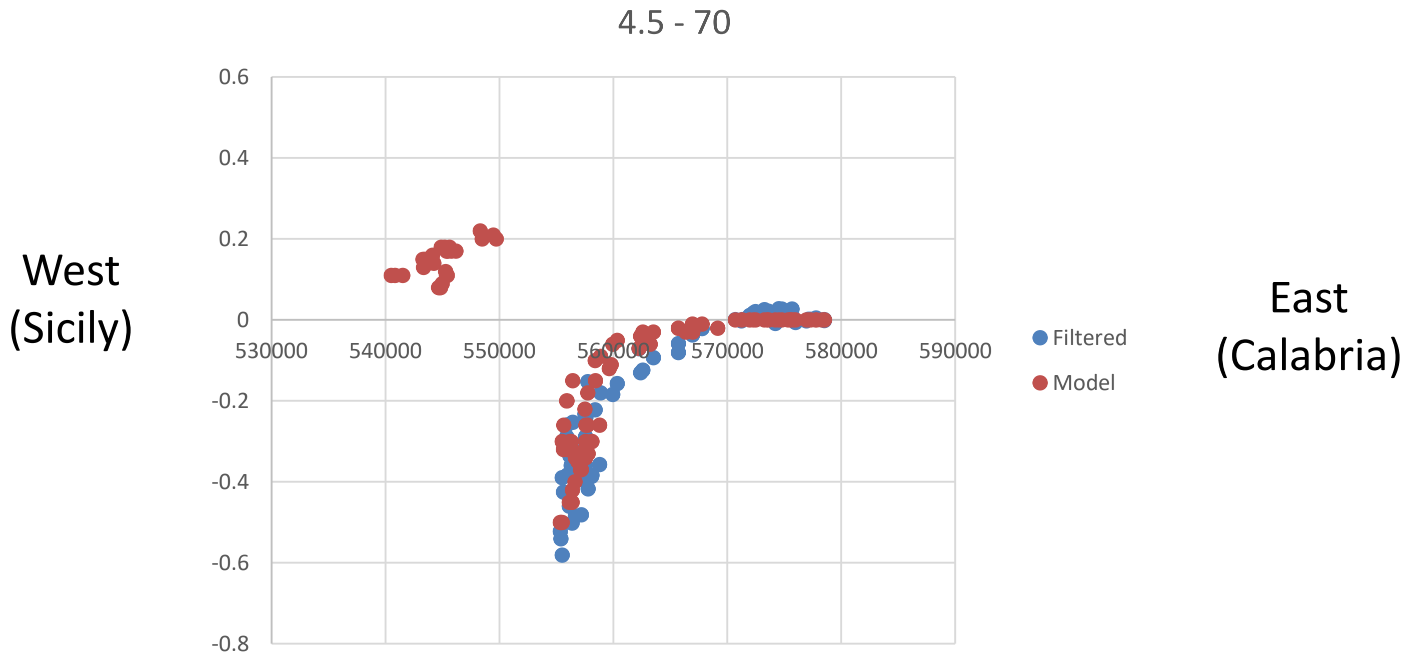

Slip = 4.5 m; Dip = 70 degrees

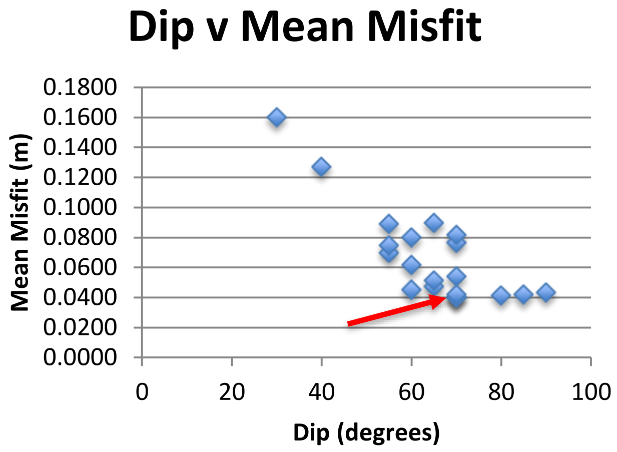

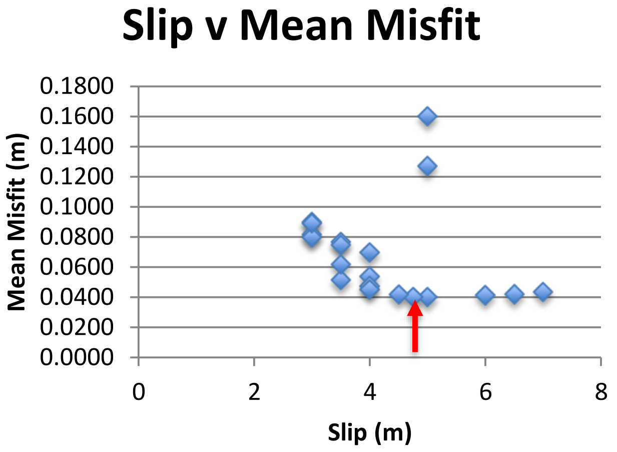

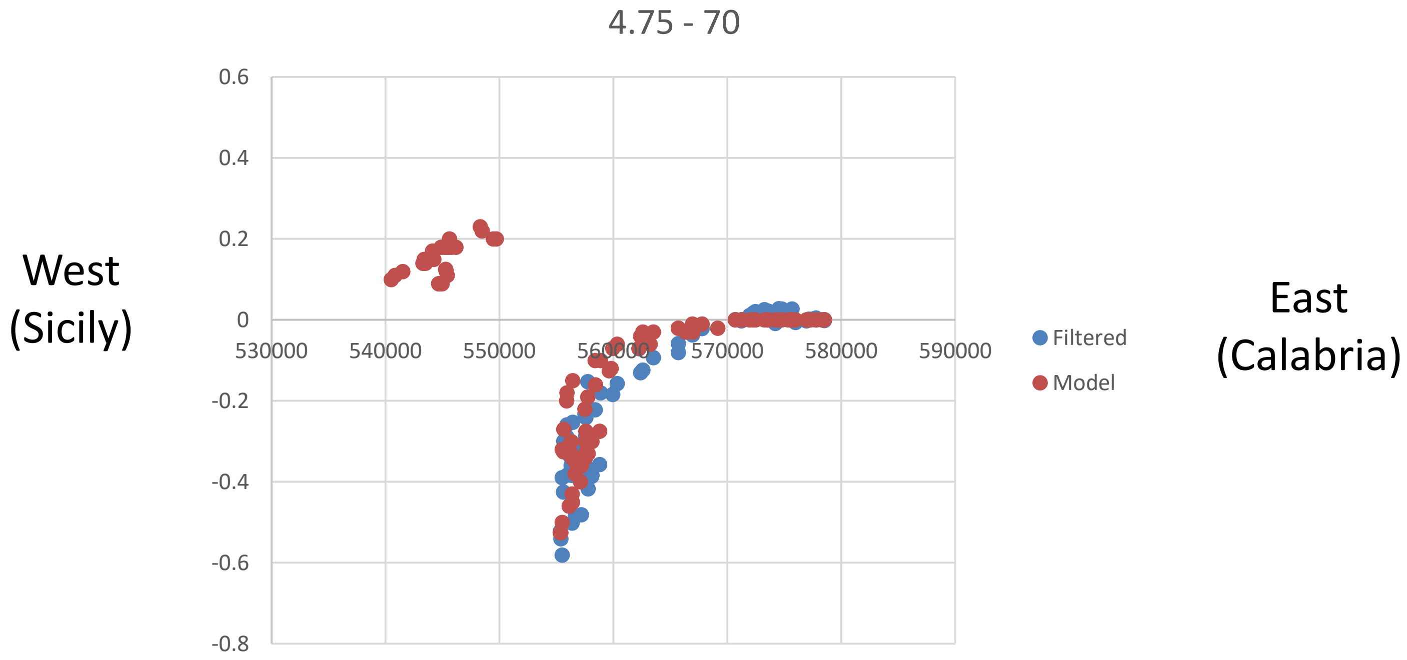

Slip = 4.75 m; Dip = 70 degrees

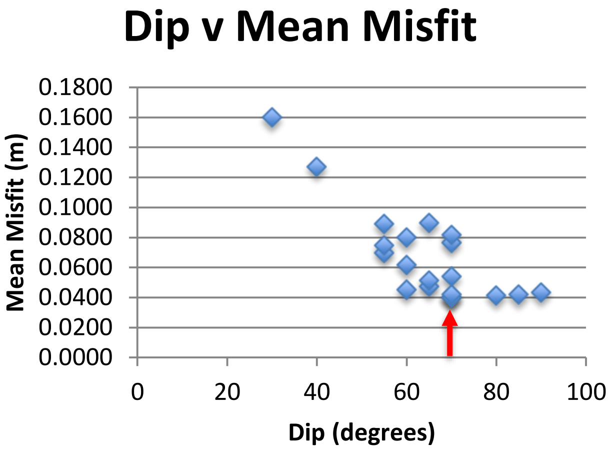

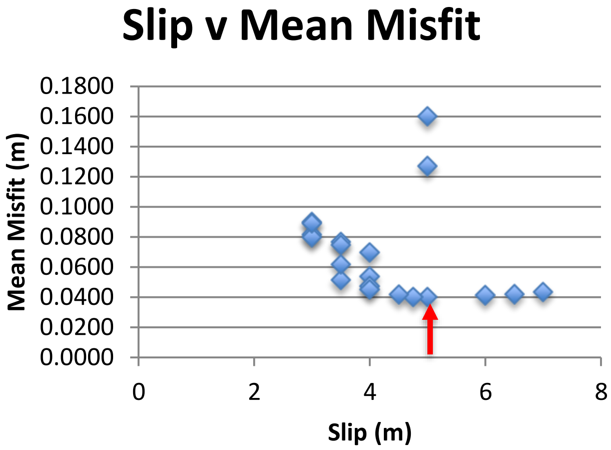

West
(Sicily)

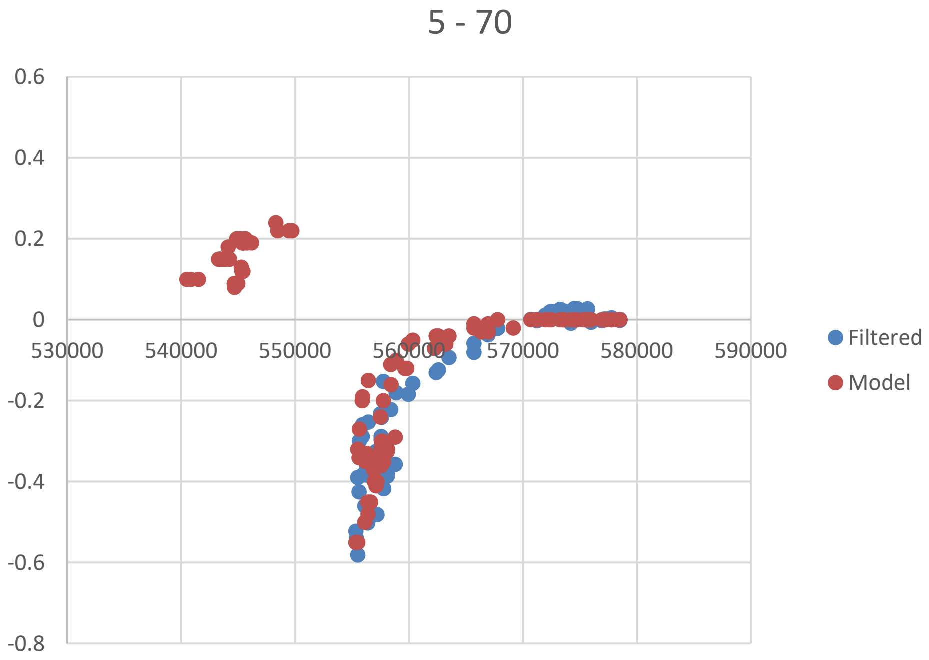

East
(Calabria)

Slip = 5 m; Dip = 70 degrees

Best fit model!!!

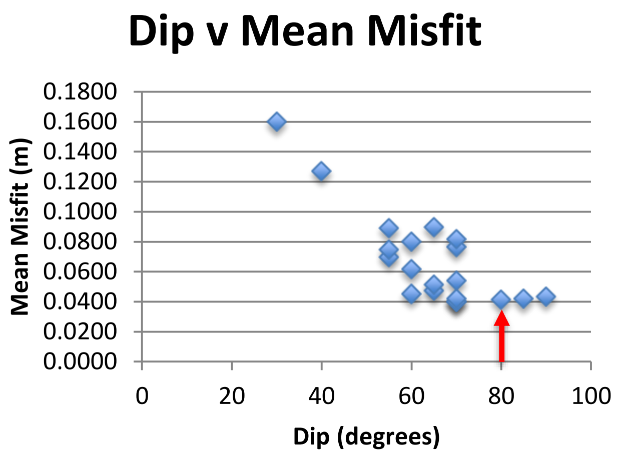

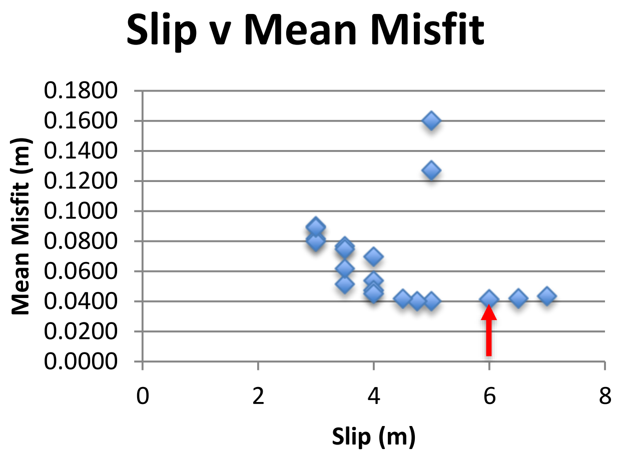

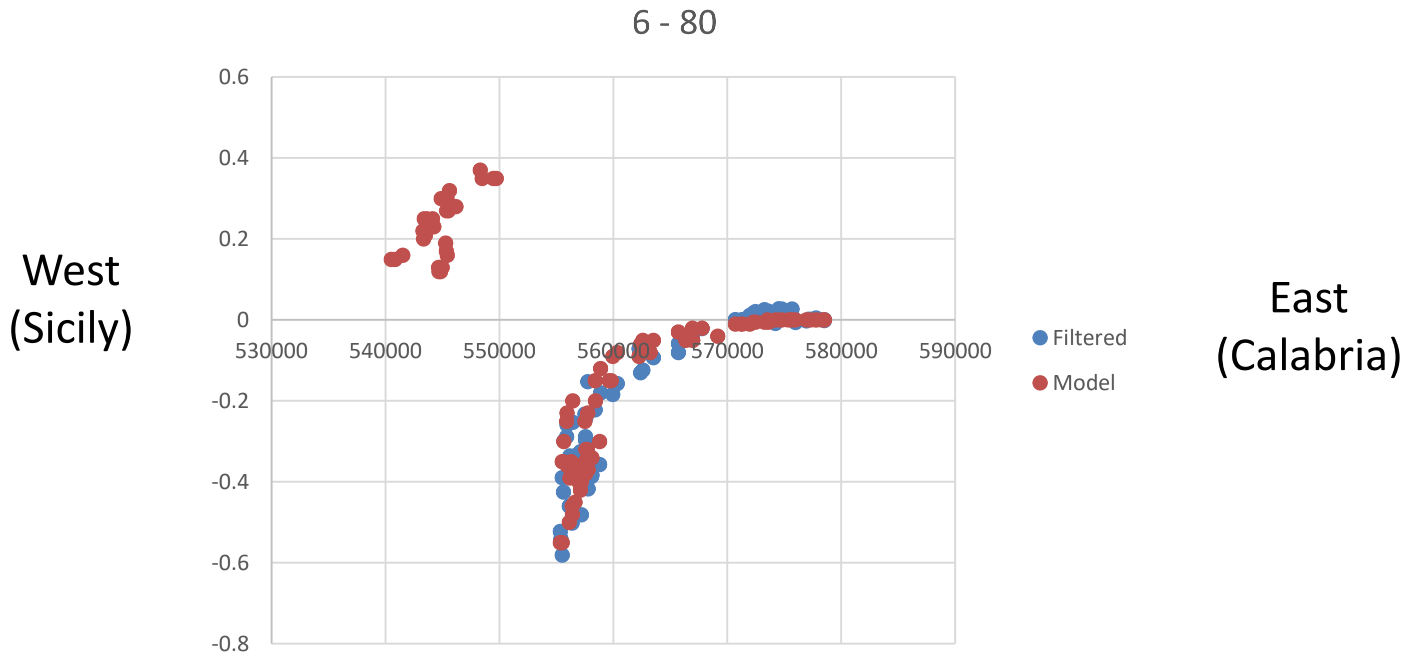

Slip = 6 m; Dip = 80 degrees

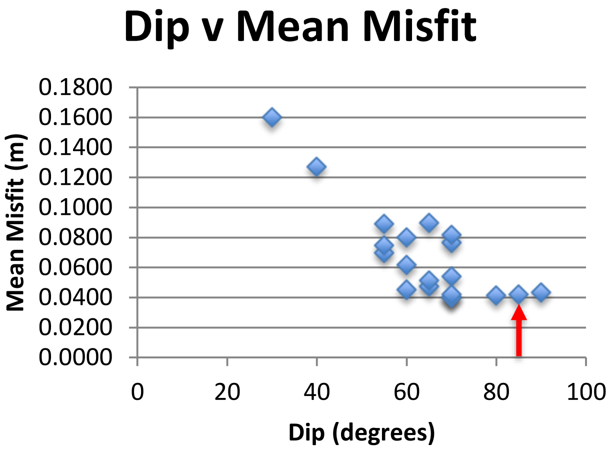

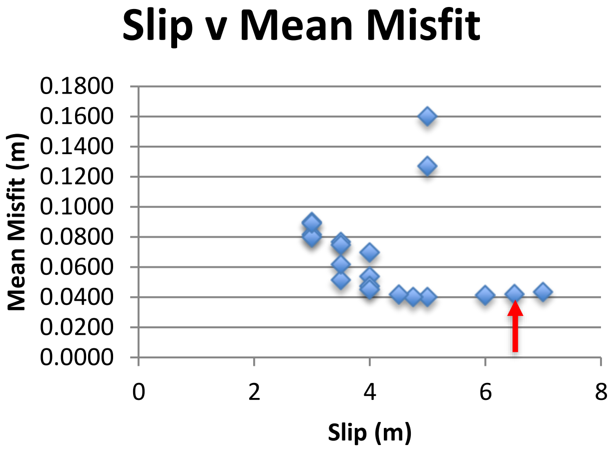

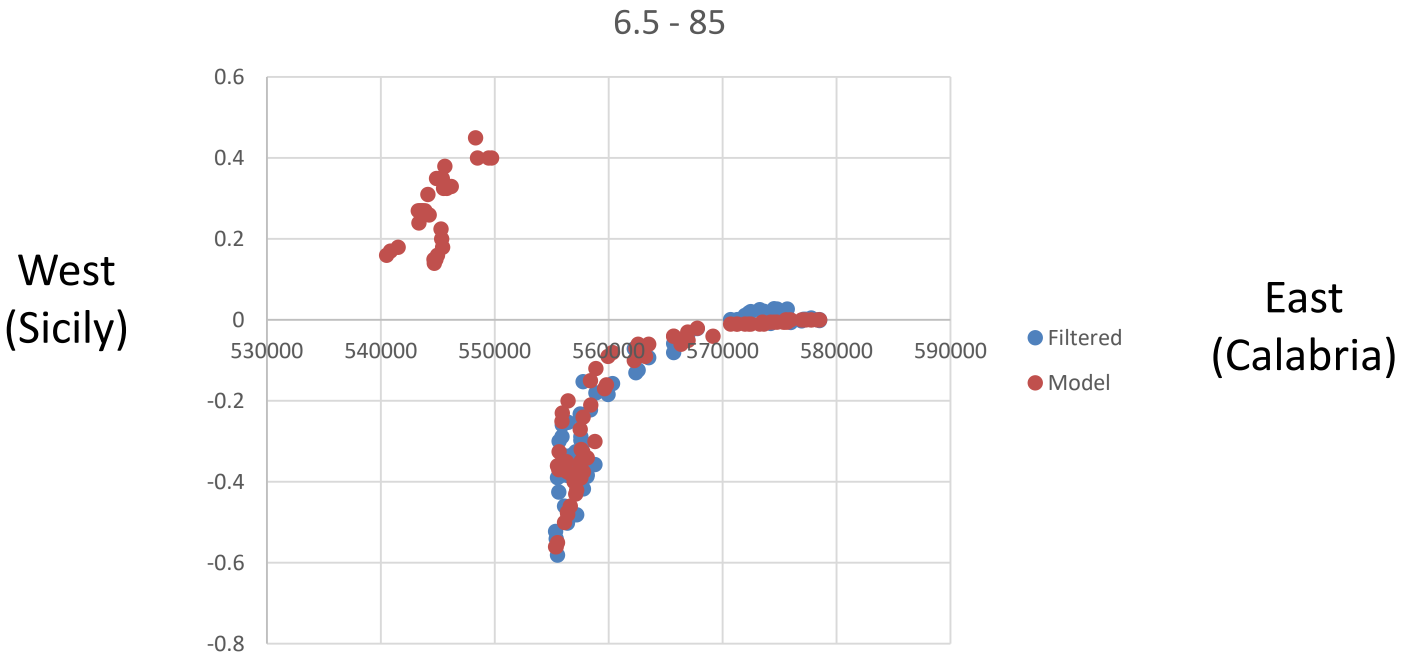

Slip = 6.5 m; Dip = 85 degrees

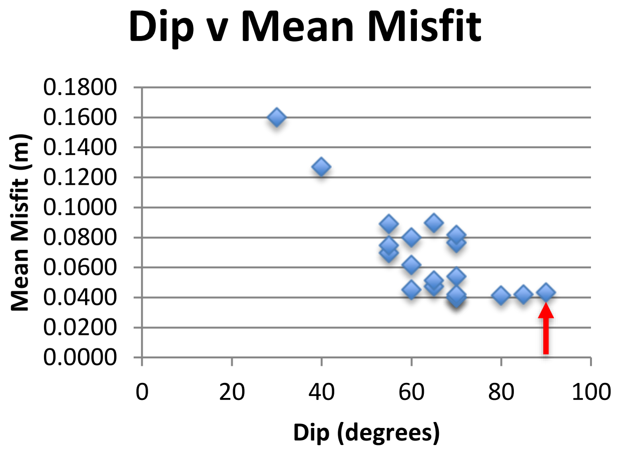

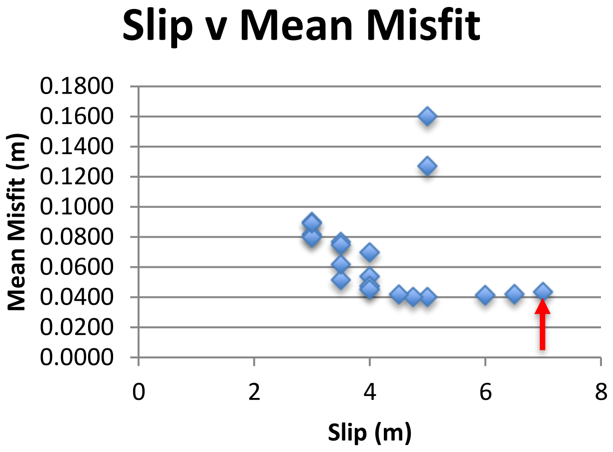

West
(Sicily)

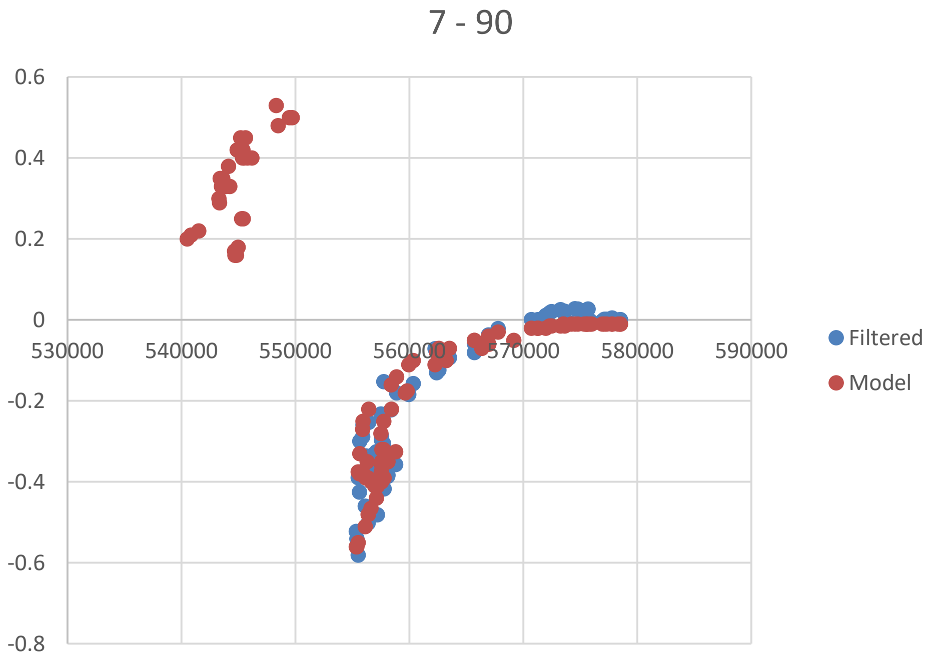

East
(Calabria)

Slip = 7 m; Dip = 90 degrees

North – South plots

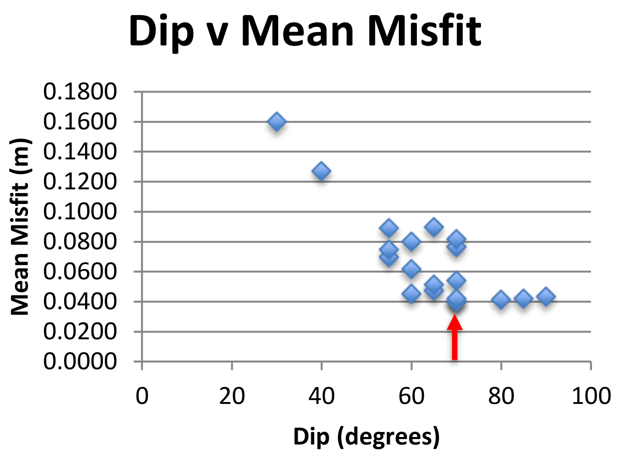

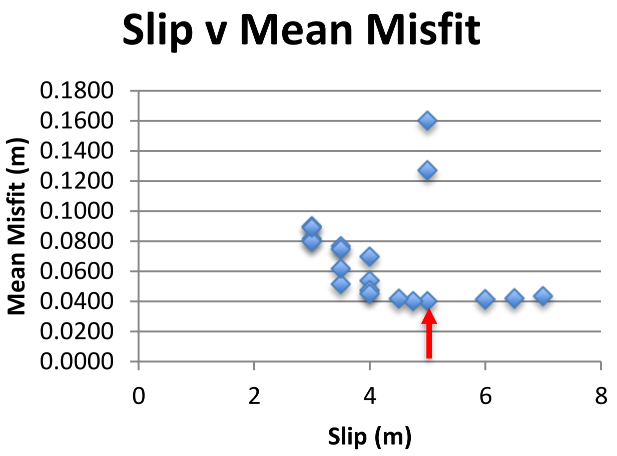

South
(Calabria)

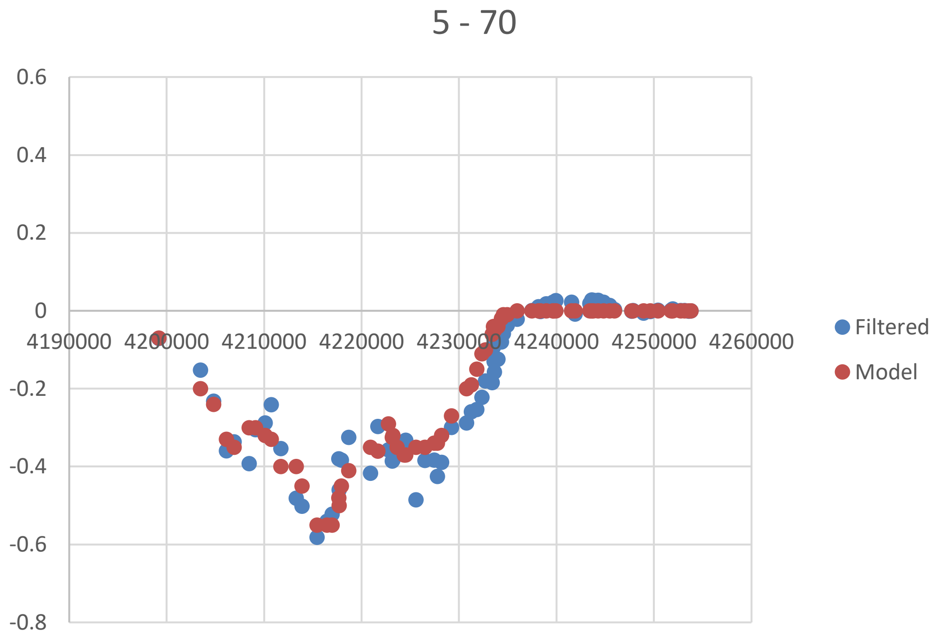

North
(Calabria)

Slip = 5 m; Dip = 70 degrees **Best fit model!!!**

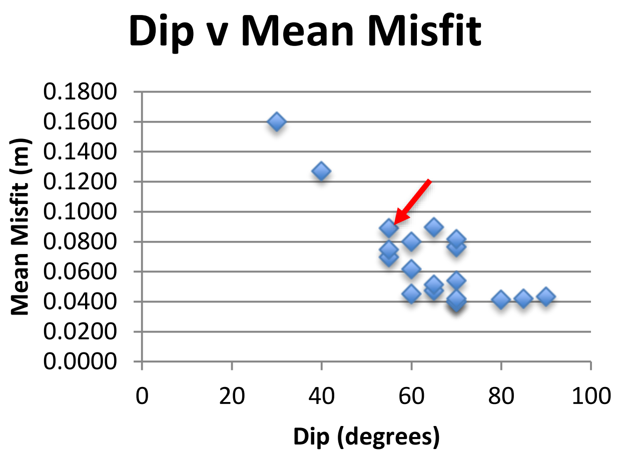

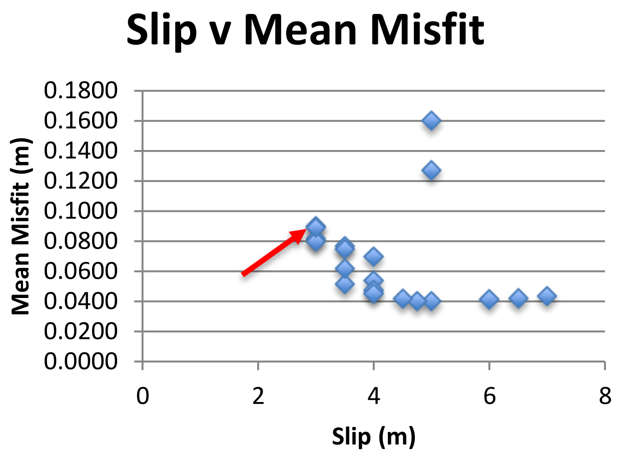

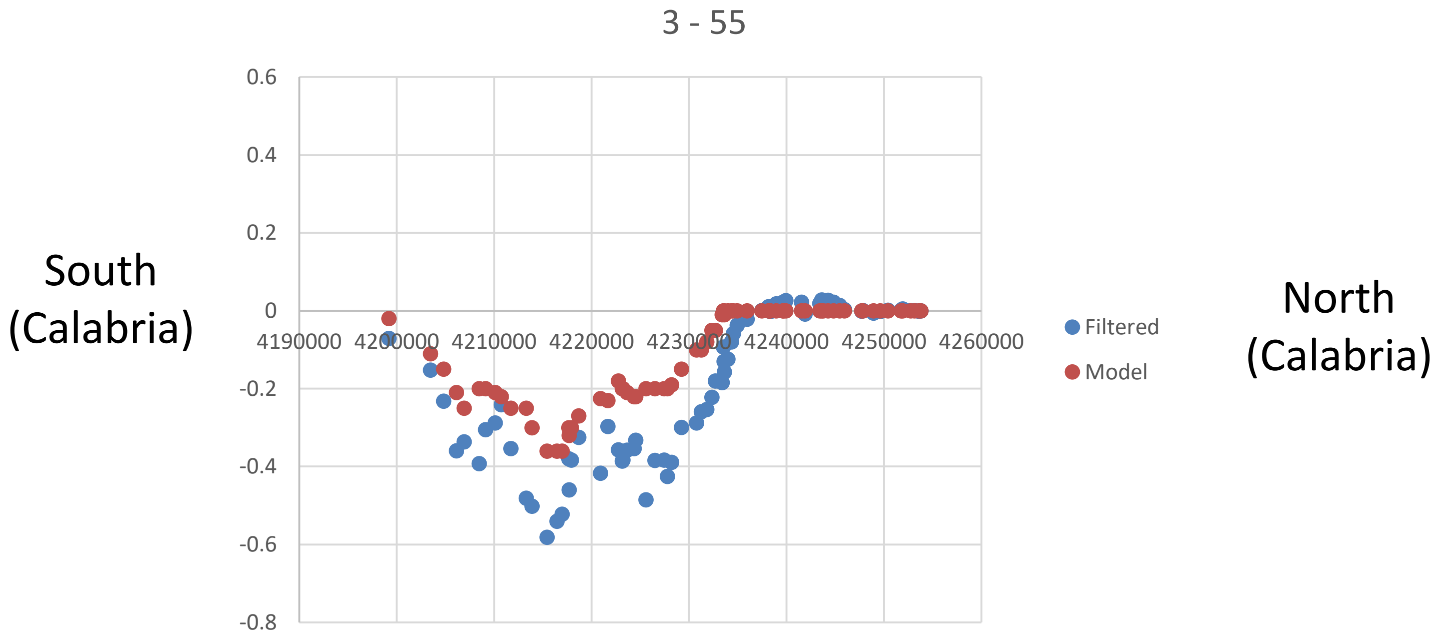

Slip = 3 m; Dip = 55 degrees

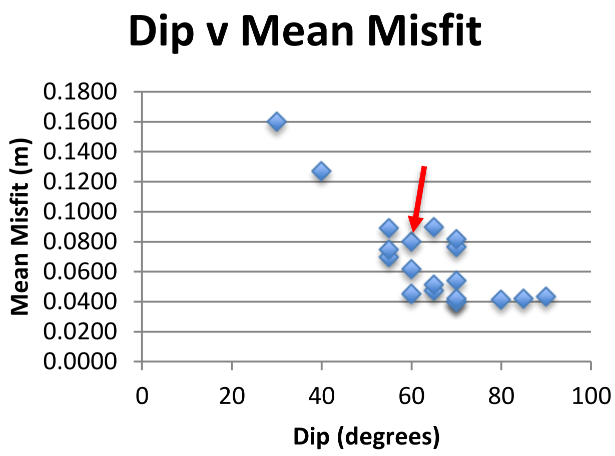

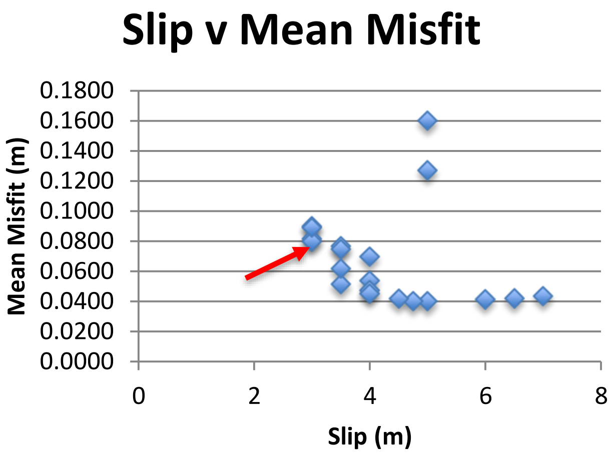

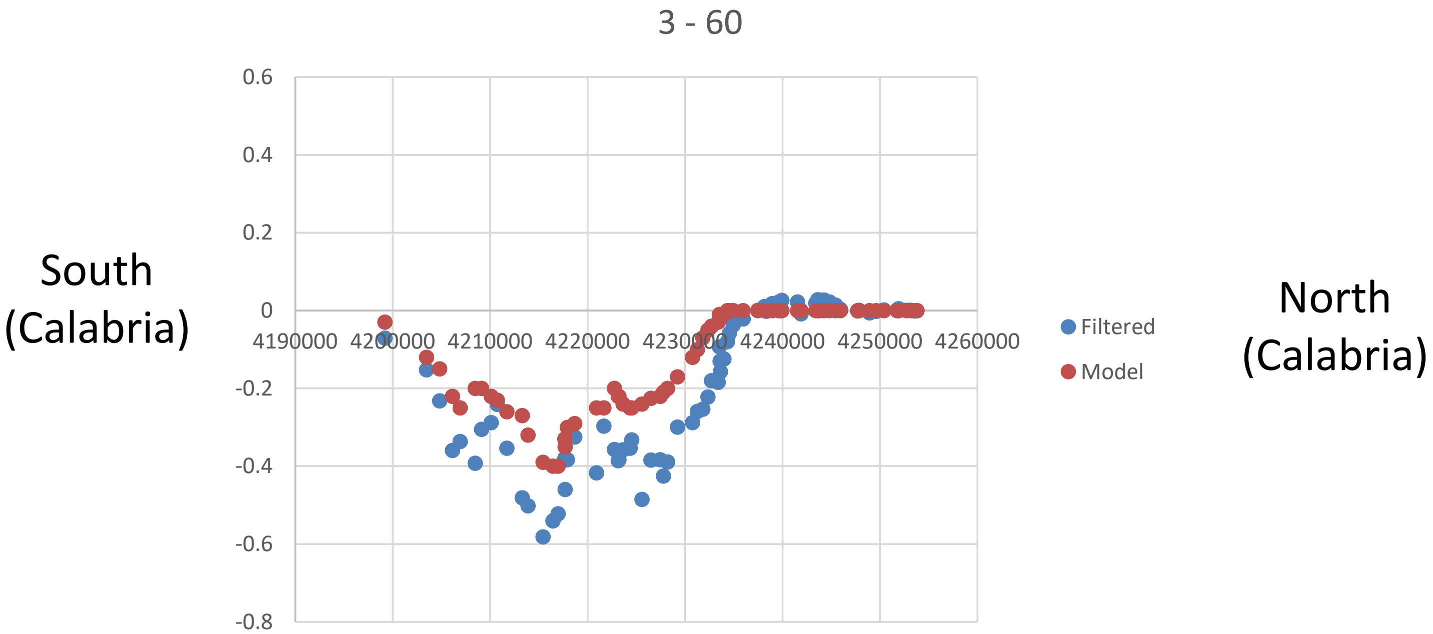

Slip = 3 m; Dip = 60 degrees

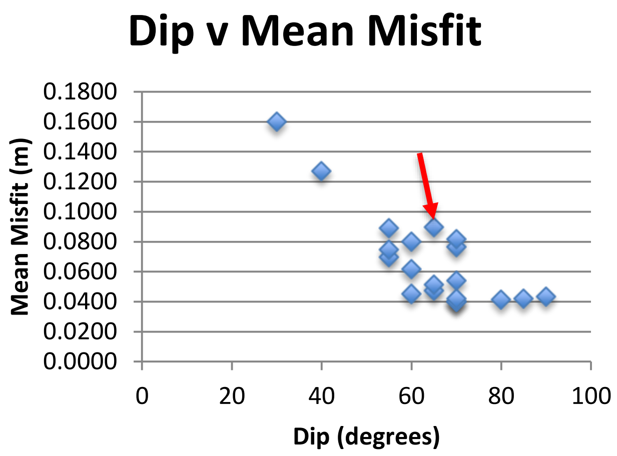

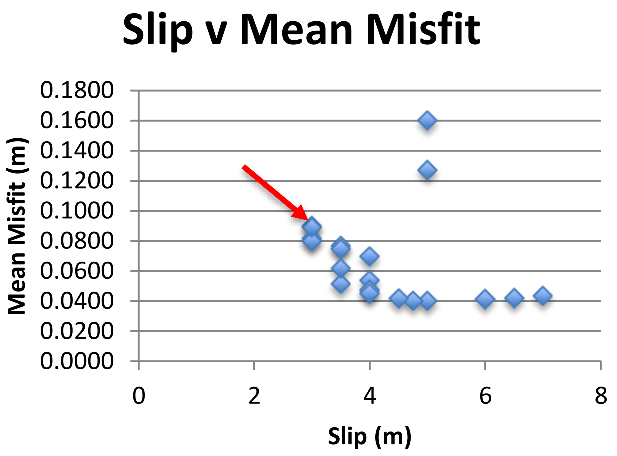

South (Calabria)

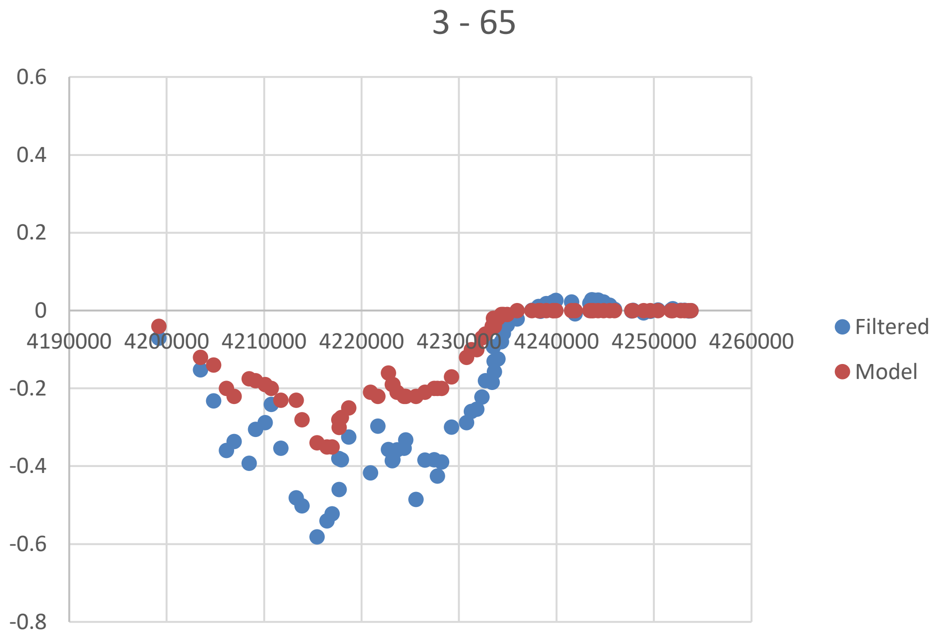

North (Calabria)

Slip = 3 m; Dip = 65 degrees

Dip v Mean Misfit

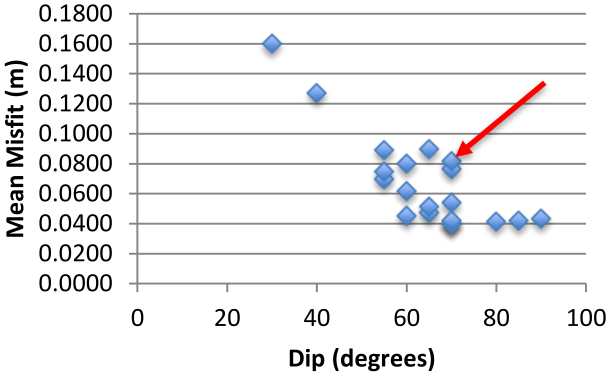

Slip v Mean Misfit

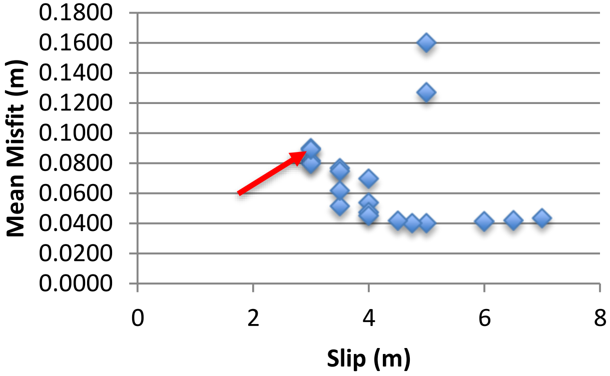

3 - 70

South (Calabria)

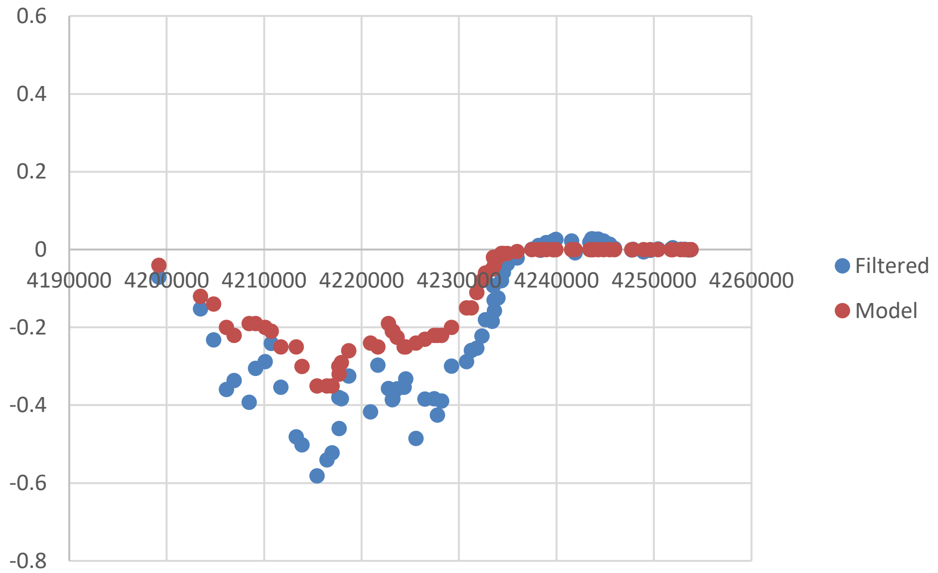

North (Calabria)

Slip = 3 m; Dip = 70 degrees

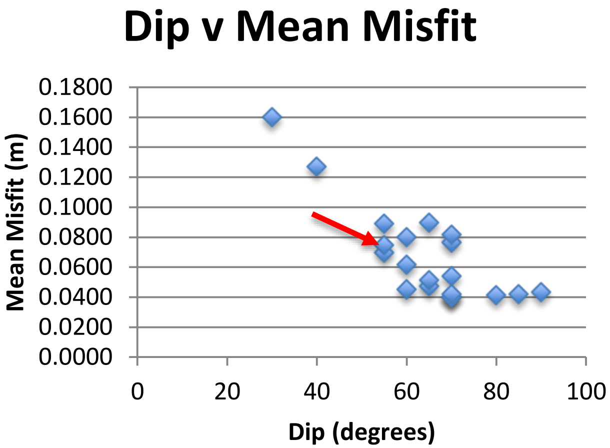

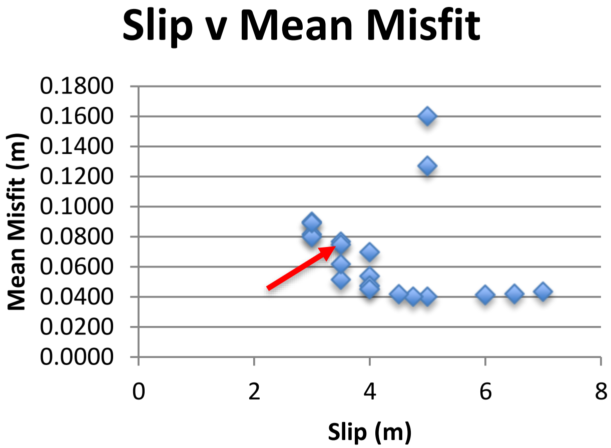

South (Calabria)

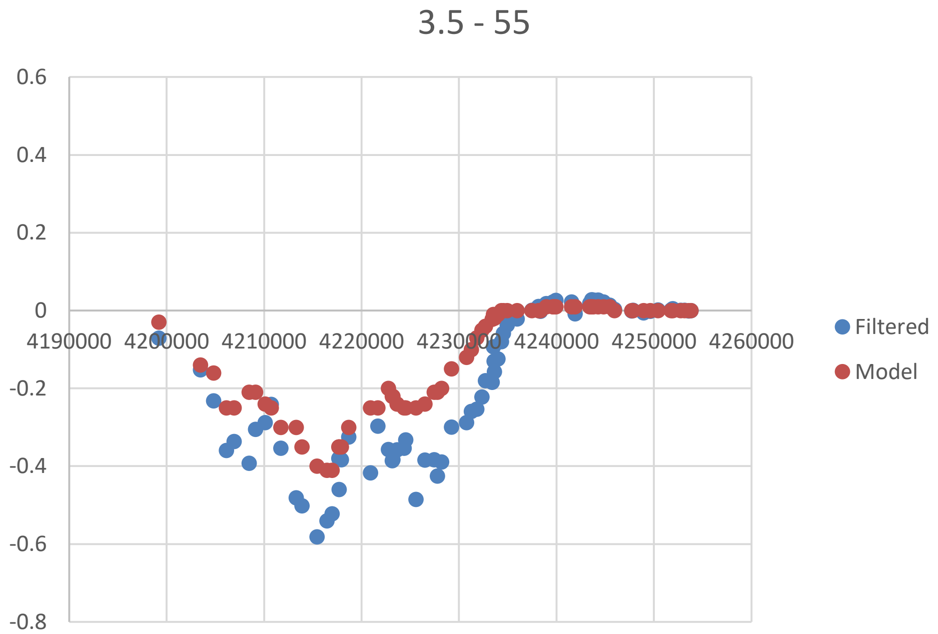

North (Calabria)

Slip = 3.5 m; Dip = 55 degrees

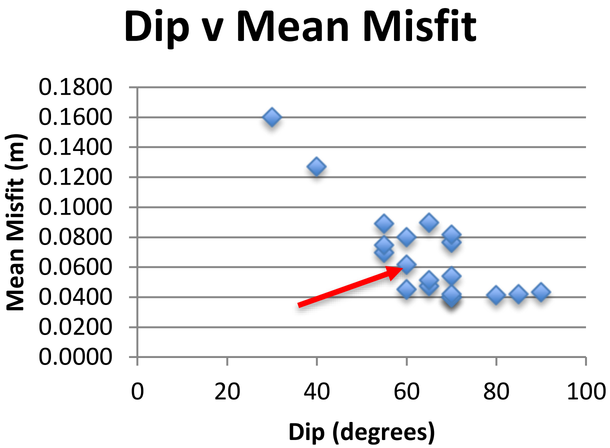

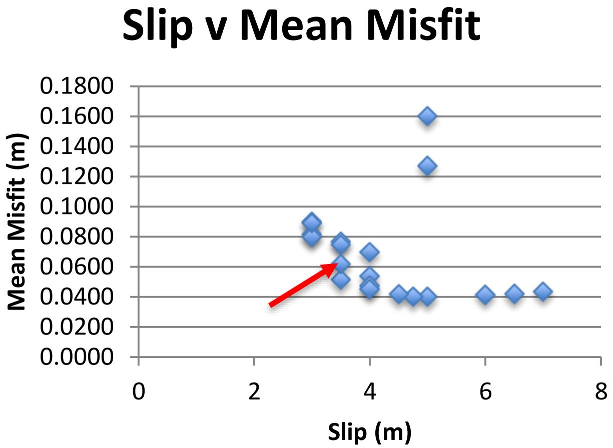

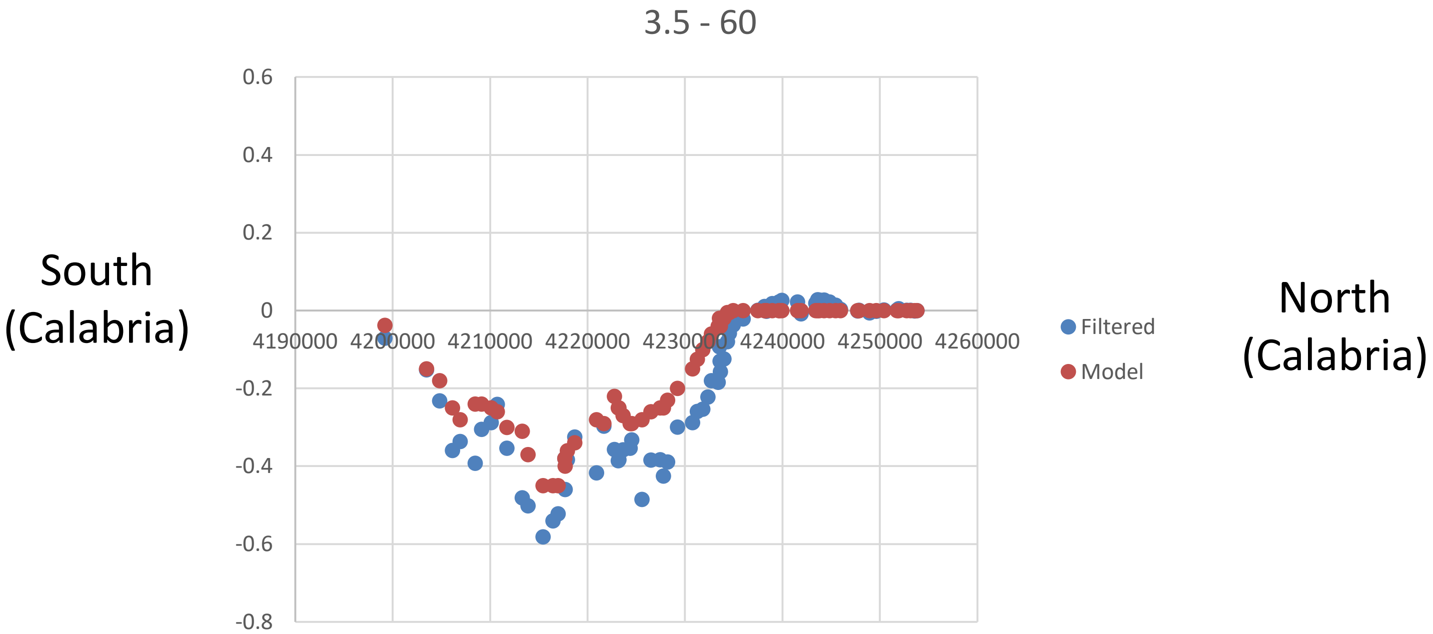

Slip = 3.5 m; Dip = 60 degrees

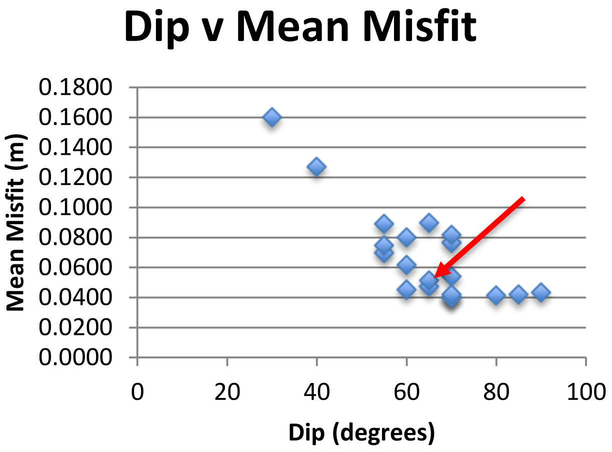

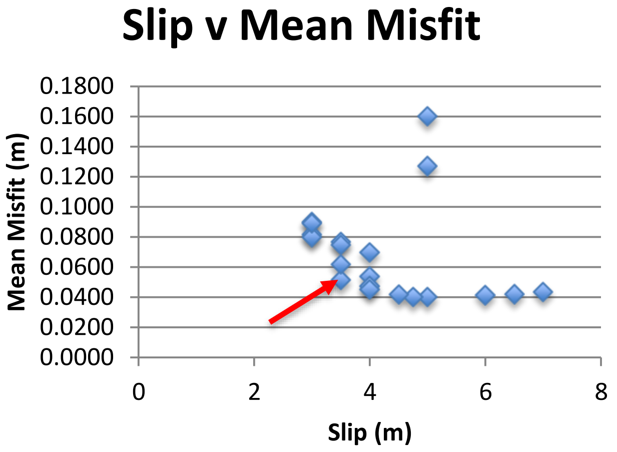

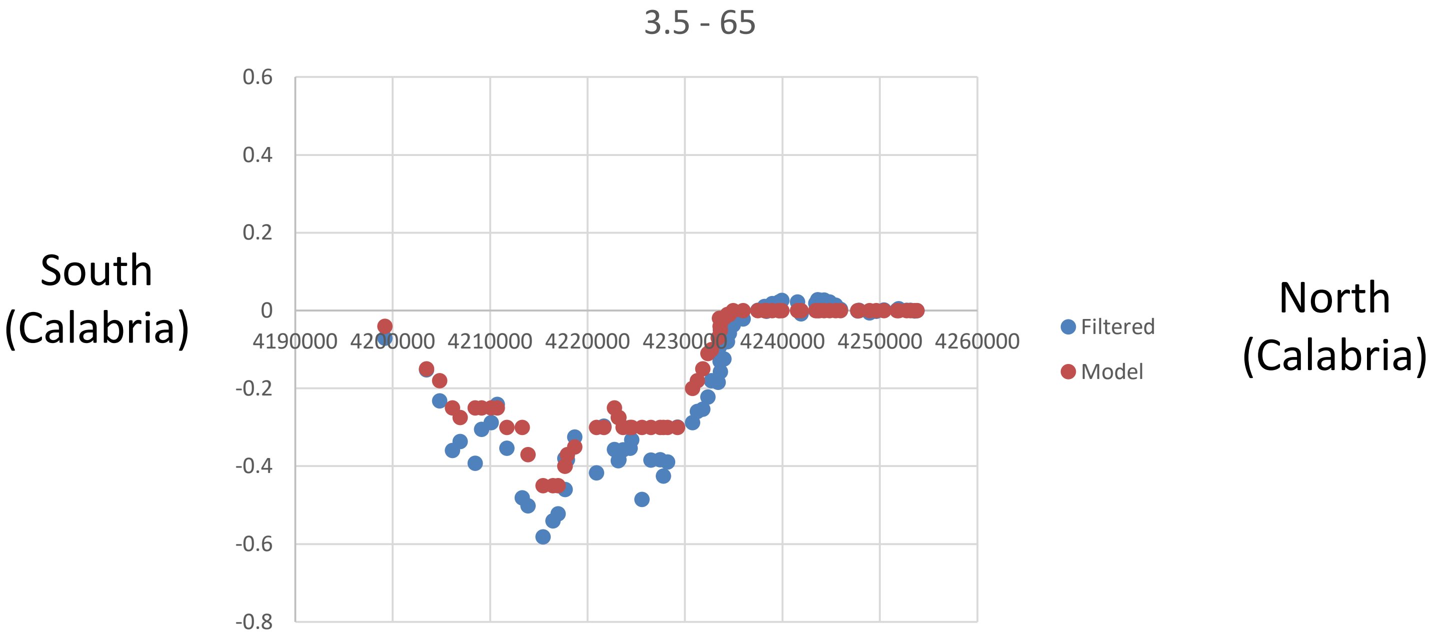

Slip = 3.5 m; Dip = 65 degrees

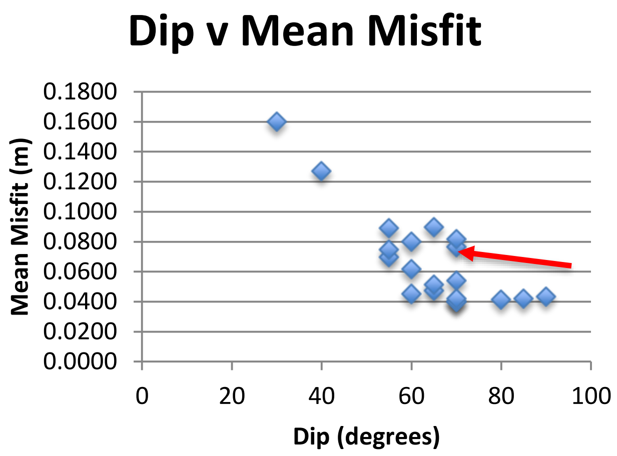

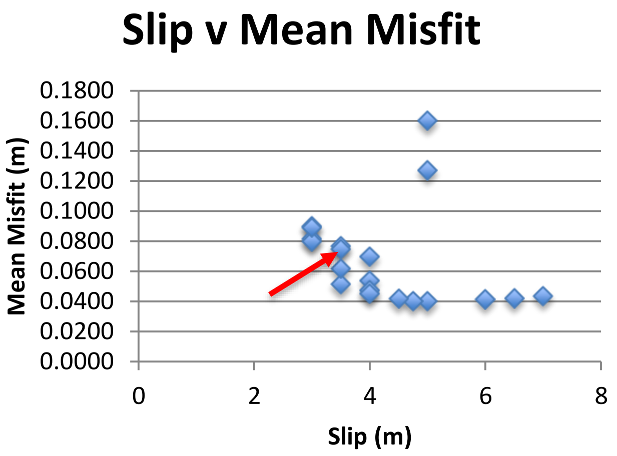

South (Calabria)

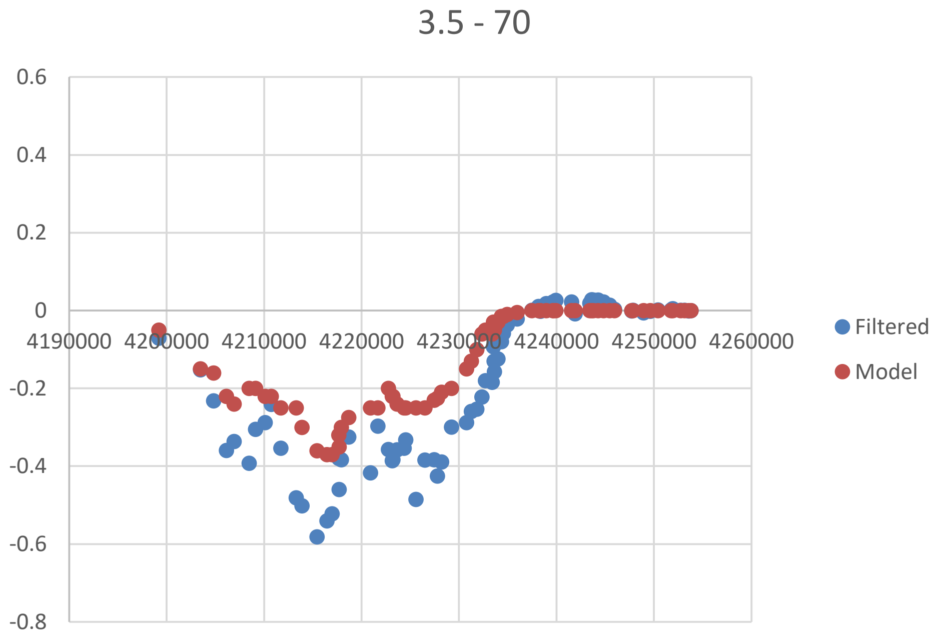

North (Calabria)

Slip = 3.5 m; Dip = 70 degrees

Dip v Mean Misfit

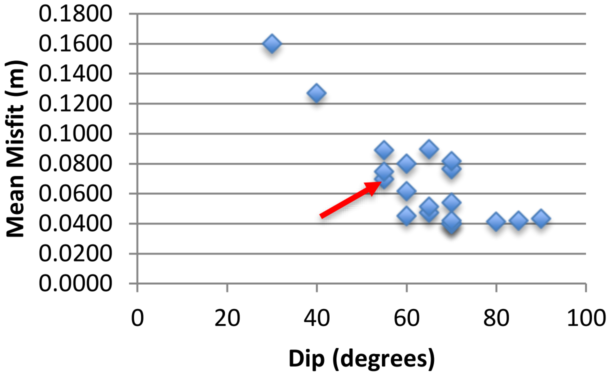

Slip v Mean Misfit

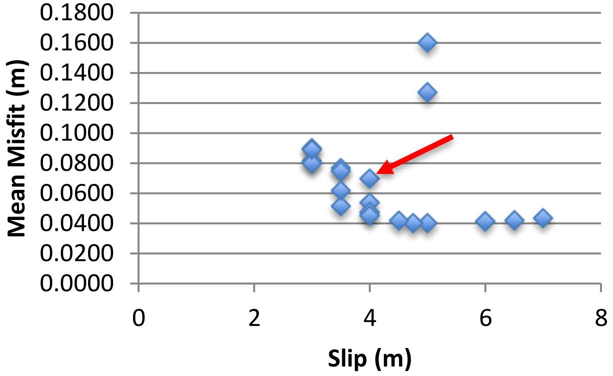

4 - 55

South (Calabria)

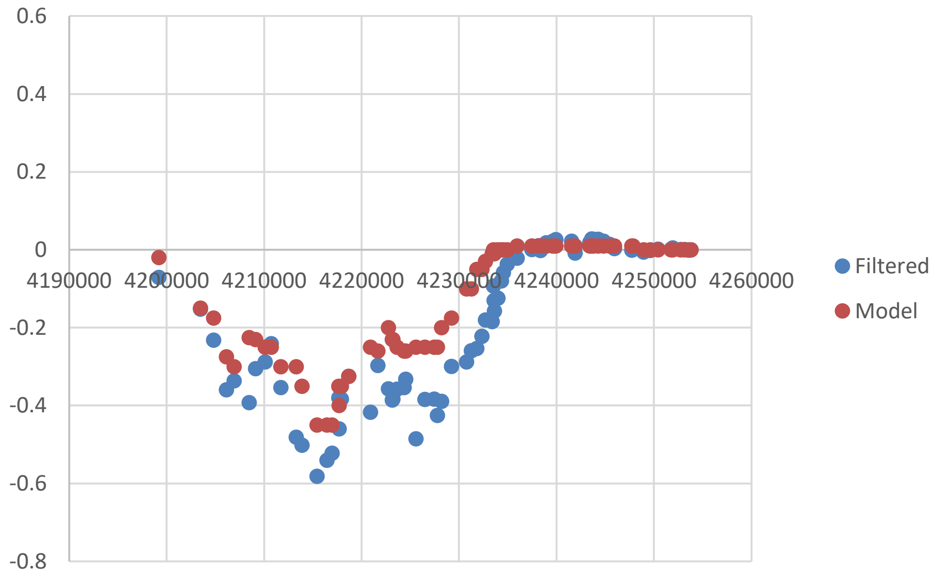

North (Calabria)

Slip = 4 m; Dip = 55 degrees

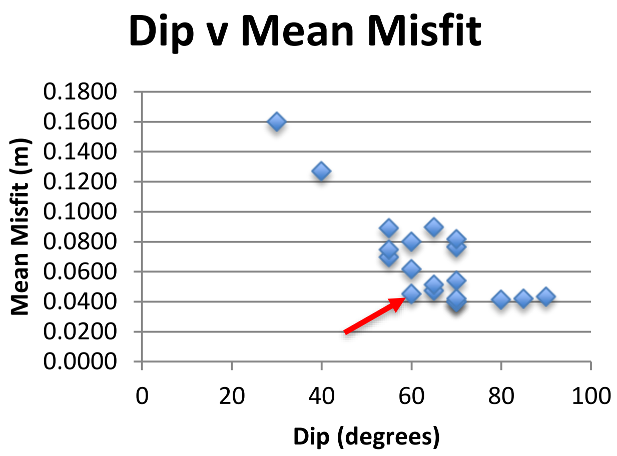

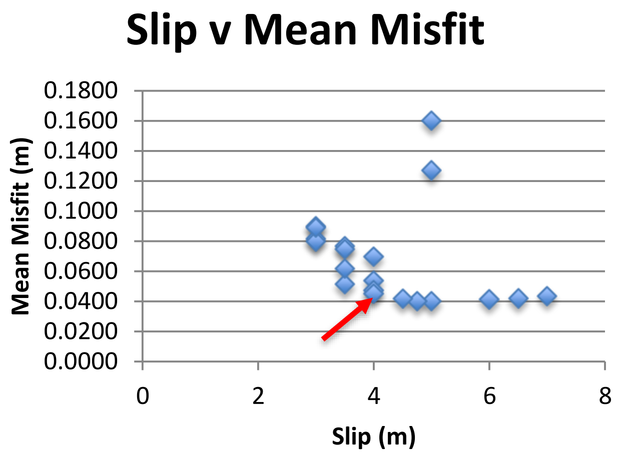

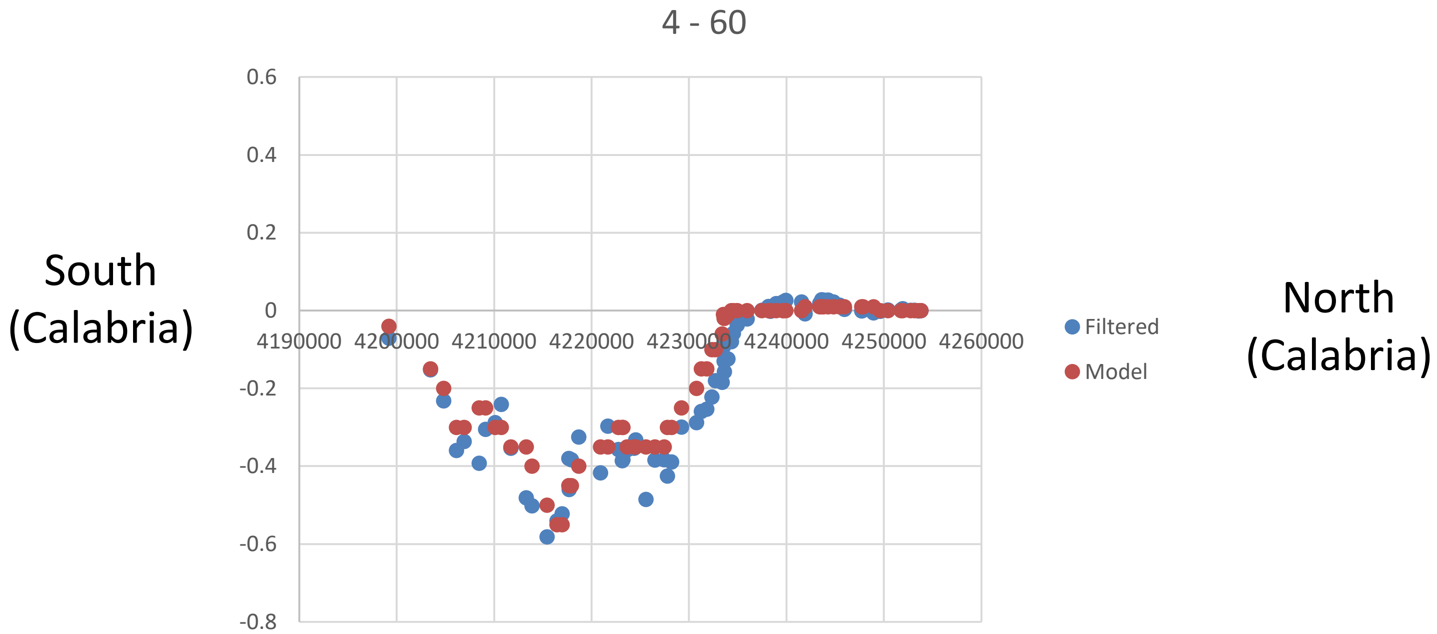

Slip = 4 m; Dip = 60 degrees

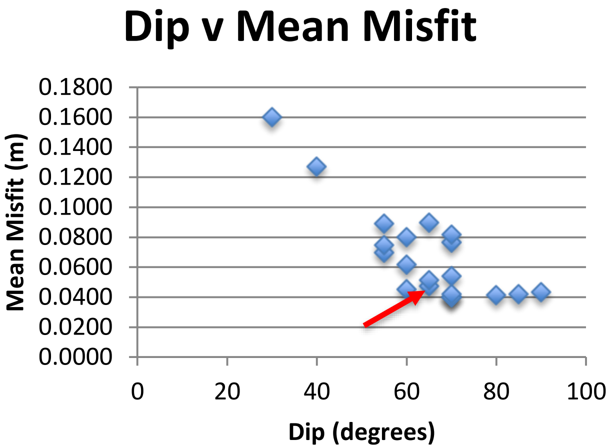

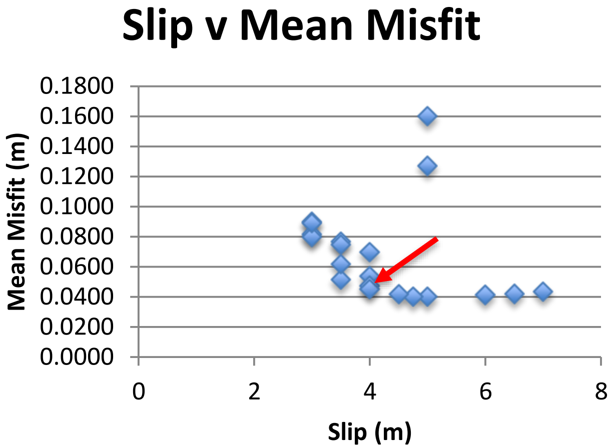

South (Calabria)

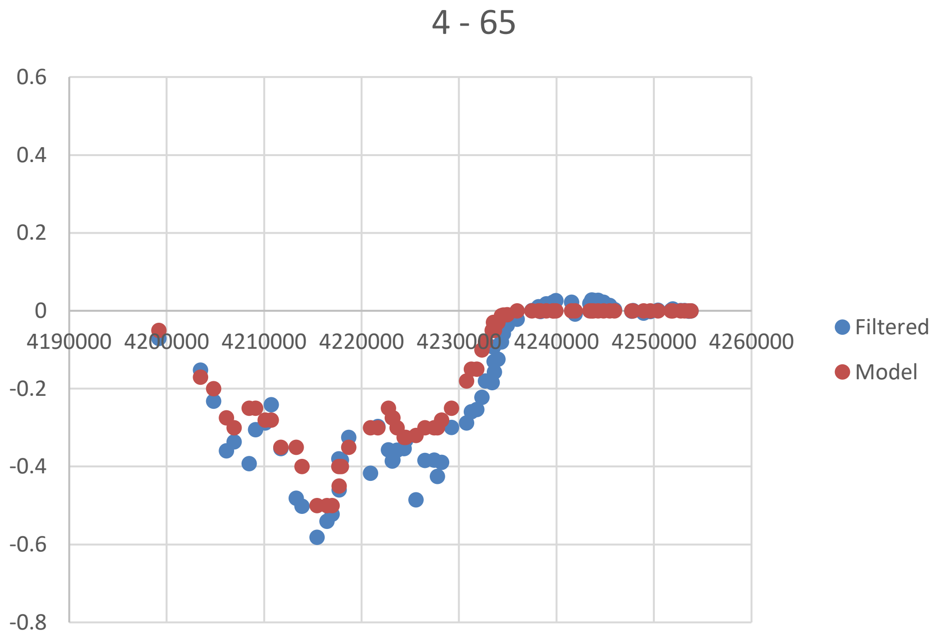

North (Calabria)

Slip = 4 m; Dip = 65 degrees

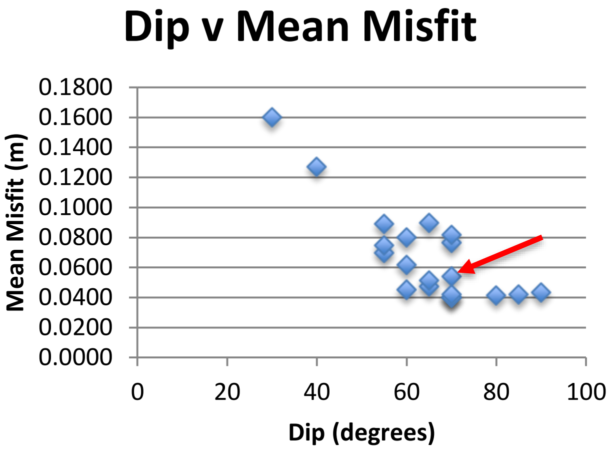

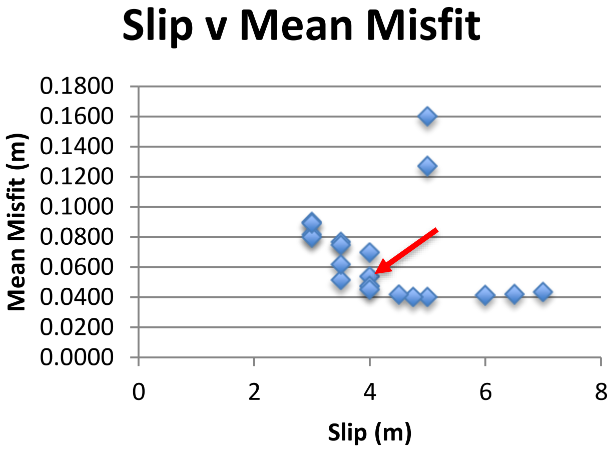

South
(Calabria)

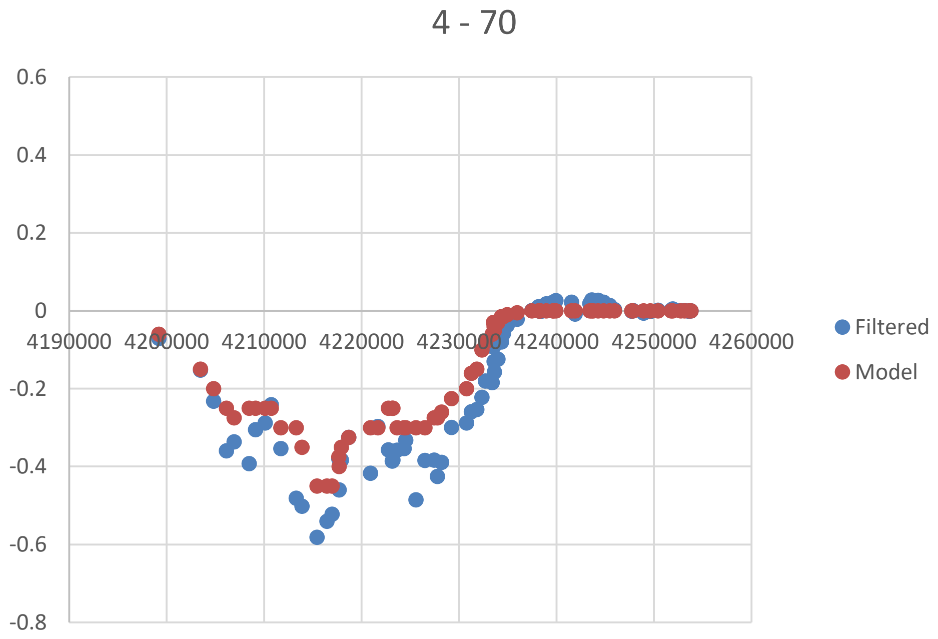

North
(Calabria)

Slip = 4 m; Dip = 70 degrees

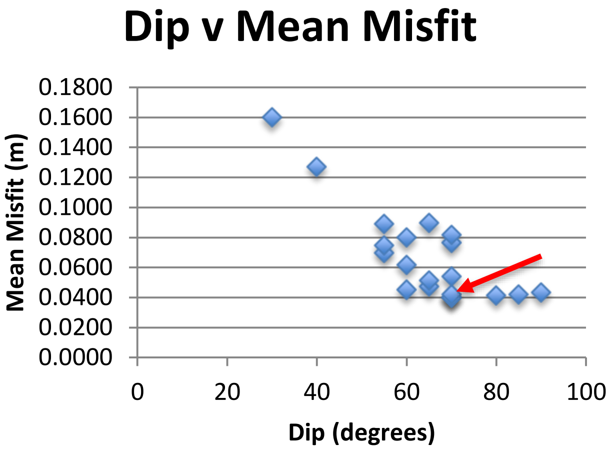

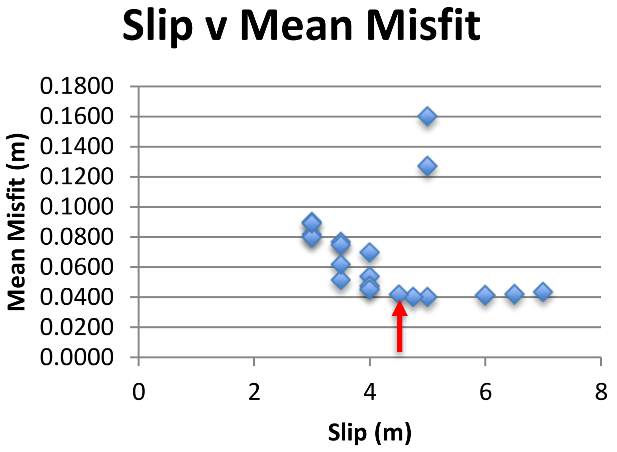

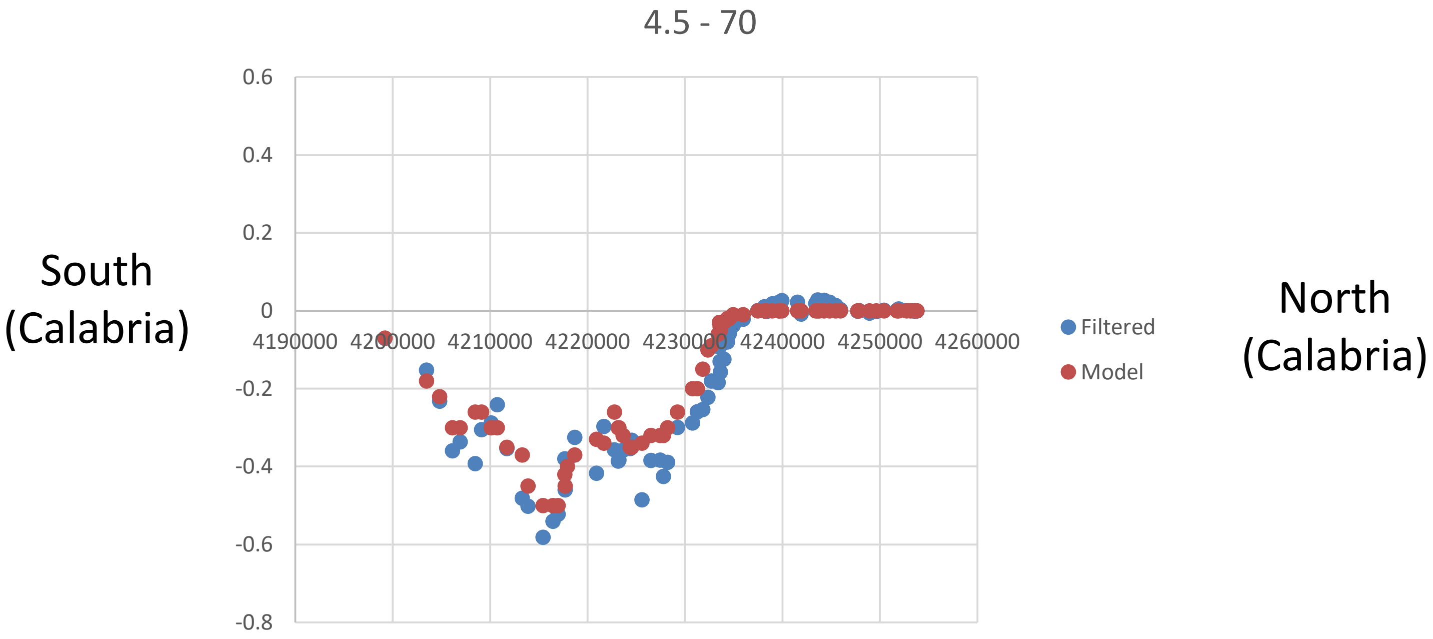

Slip = 4.5 m; Dip = 70 degrees

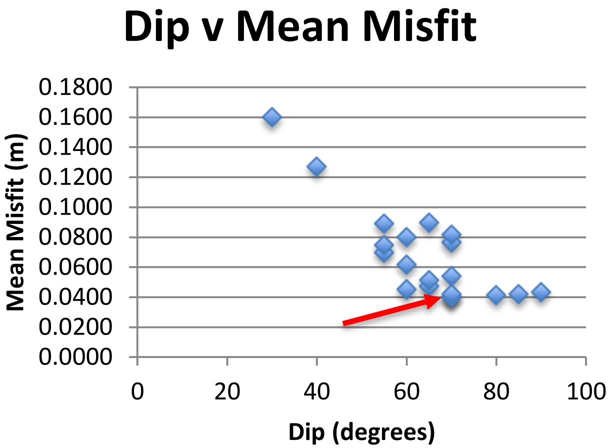

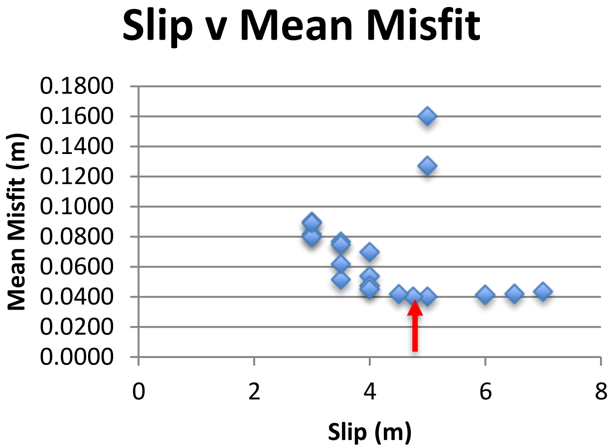

South
(Calabria)

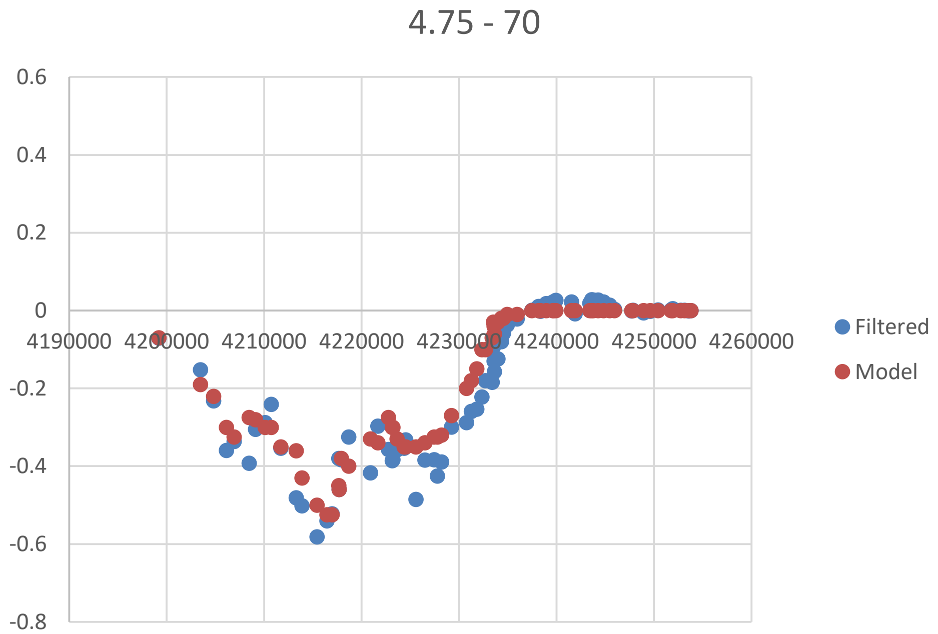

North
(Calabria)

Slip = 4.75 m; Dip = 70 degrees

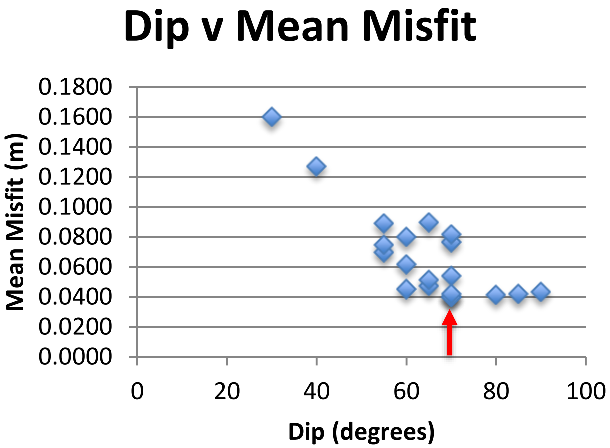

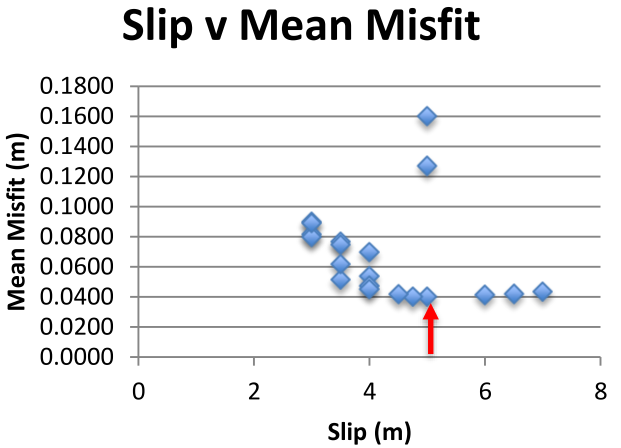

South (Calabria)

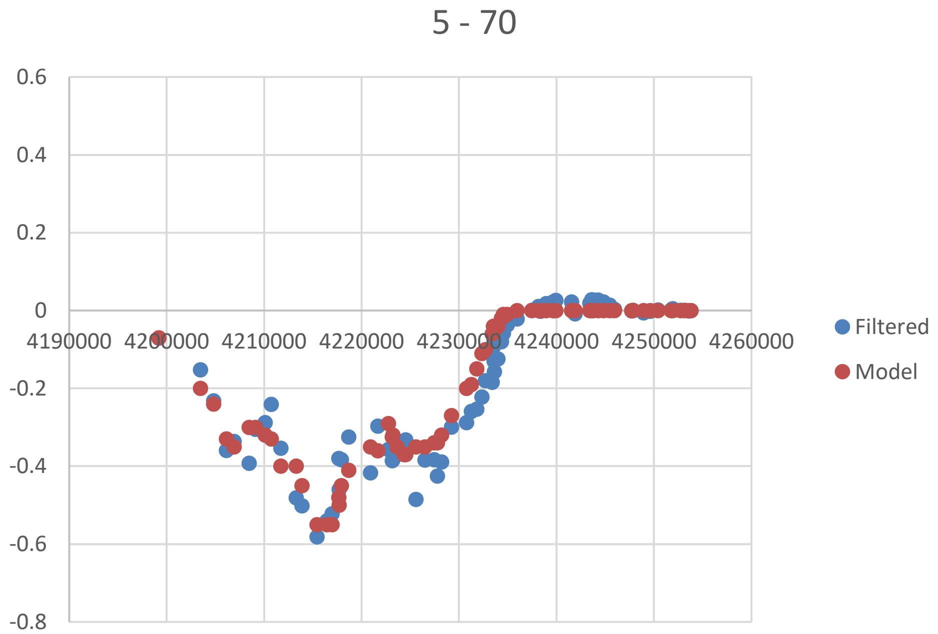

North (Calabria)

Slip = 5 m; Dip = 70 degrees

Best fit model!!!

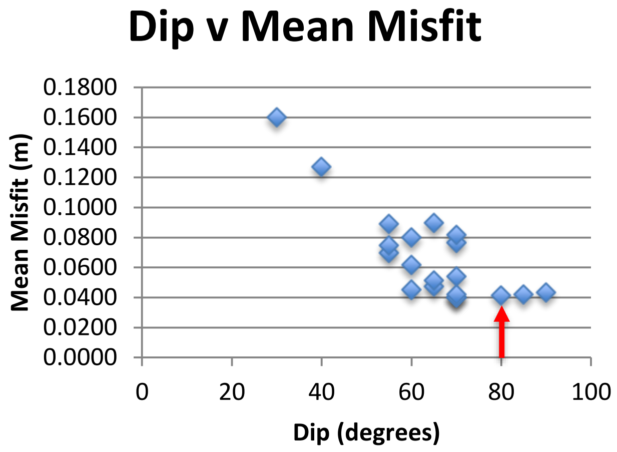

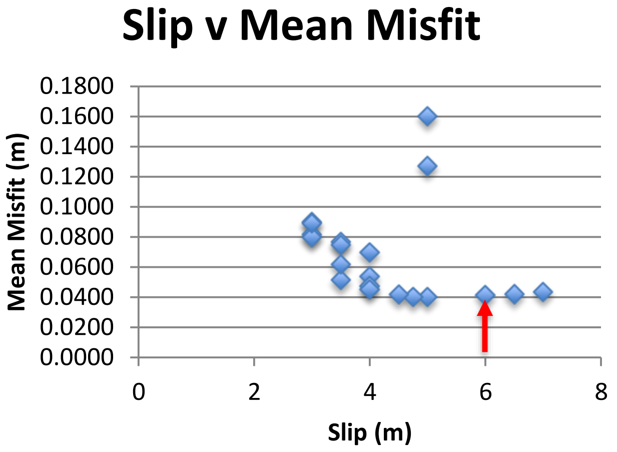

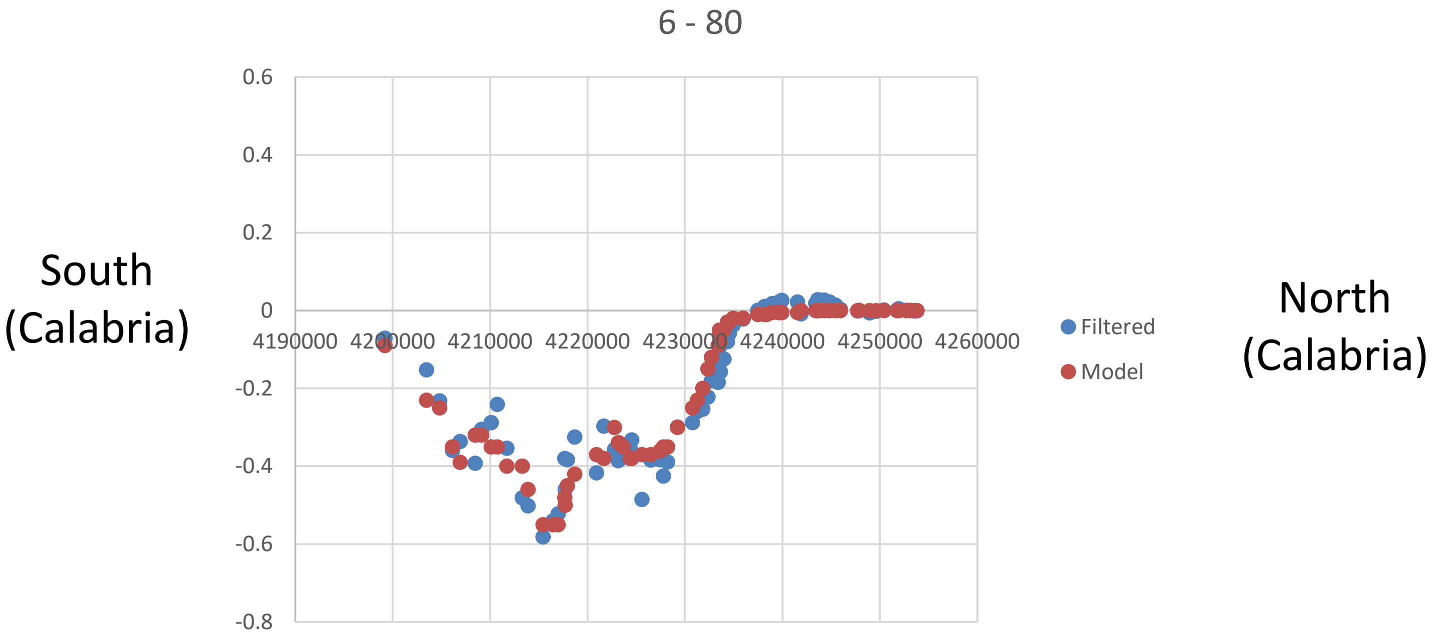

Slip = 6 m; Dip = 80 degrees

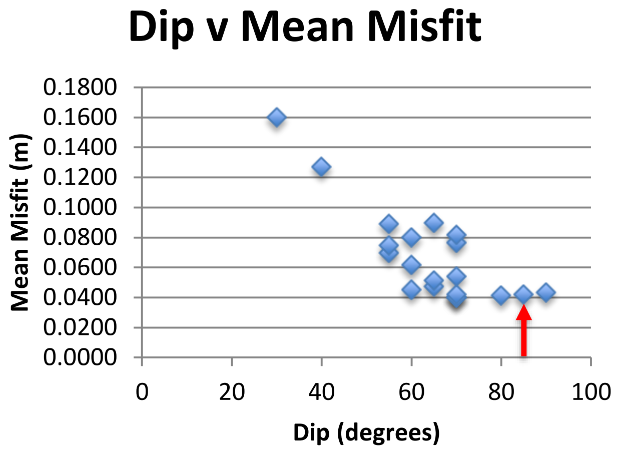

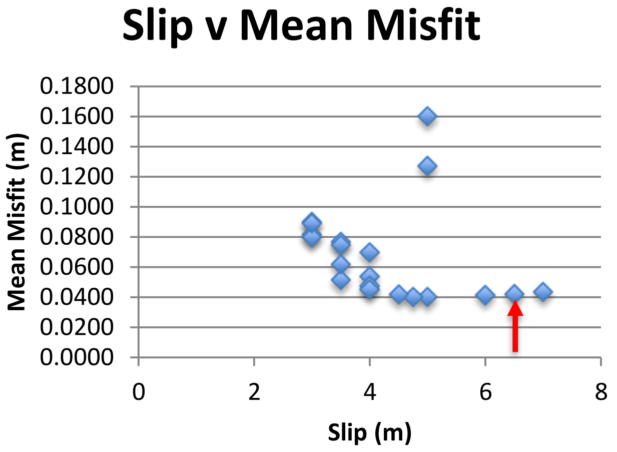

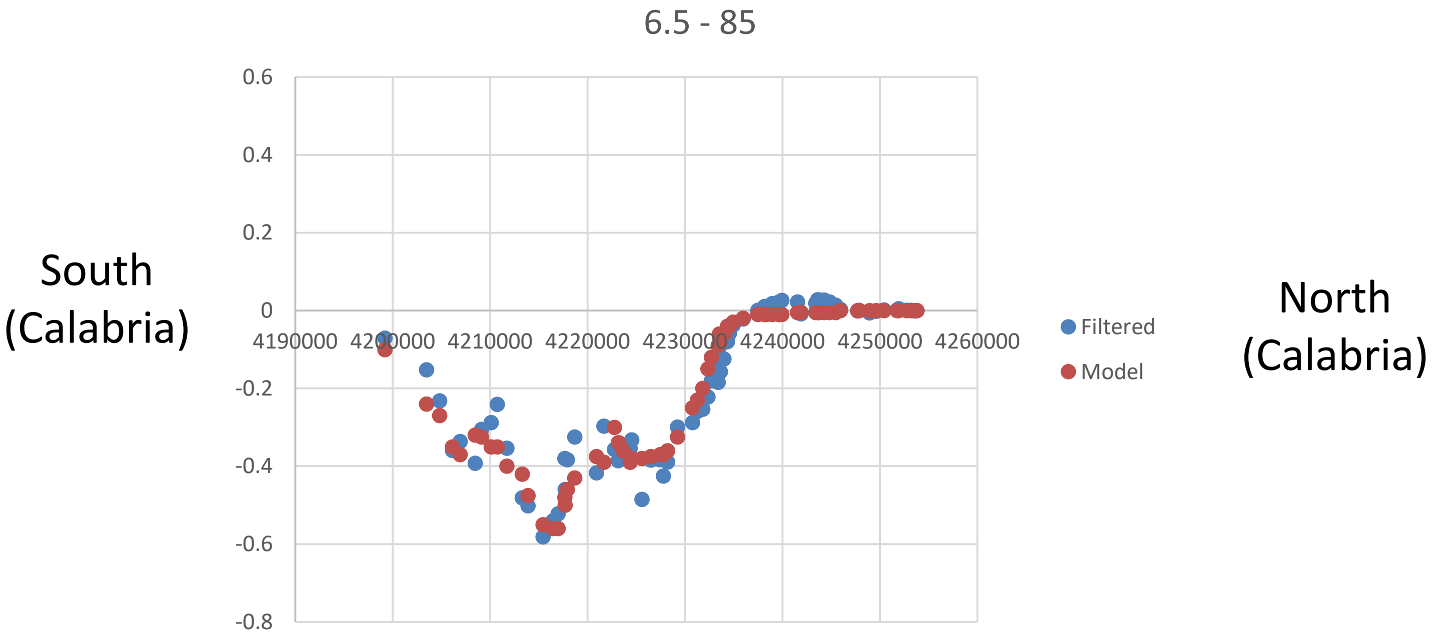

Slip = 6.5 m; Dip = 85 degrees

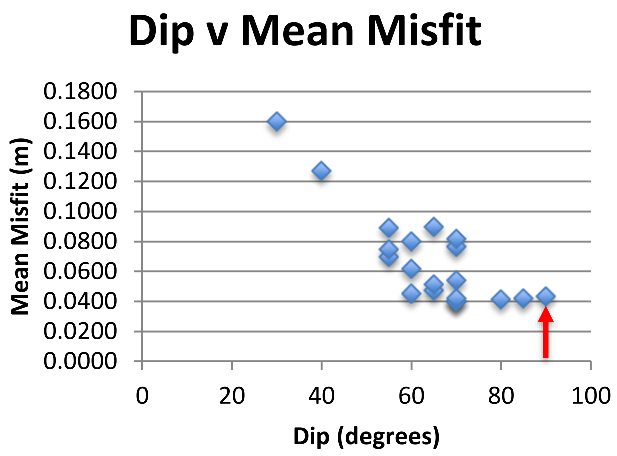

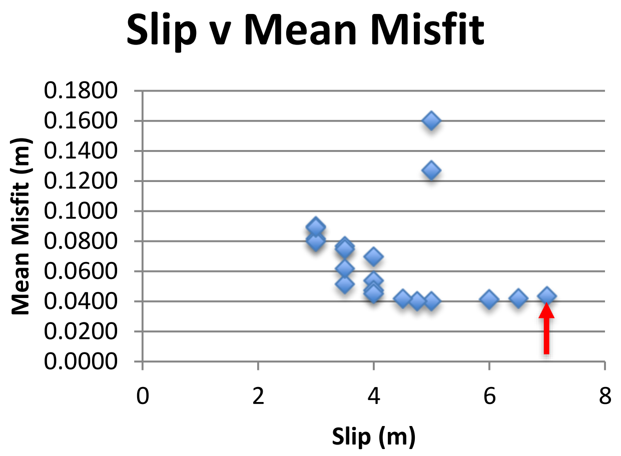

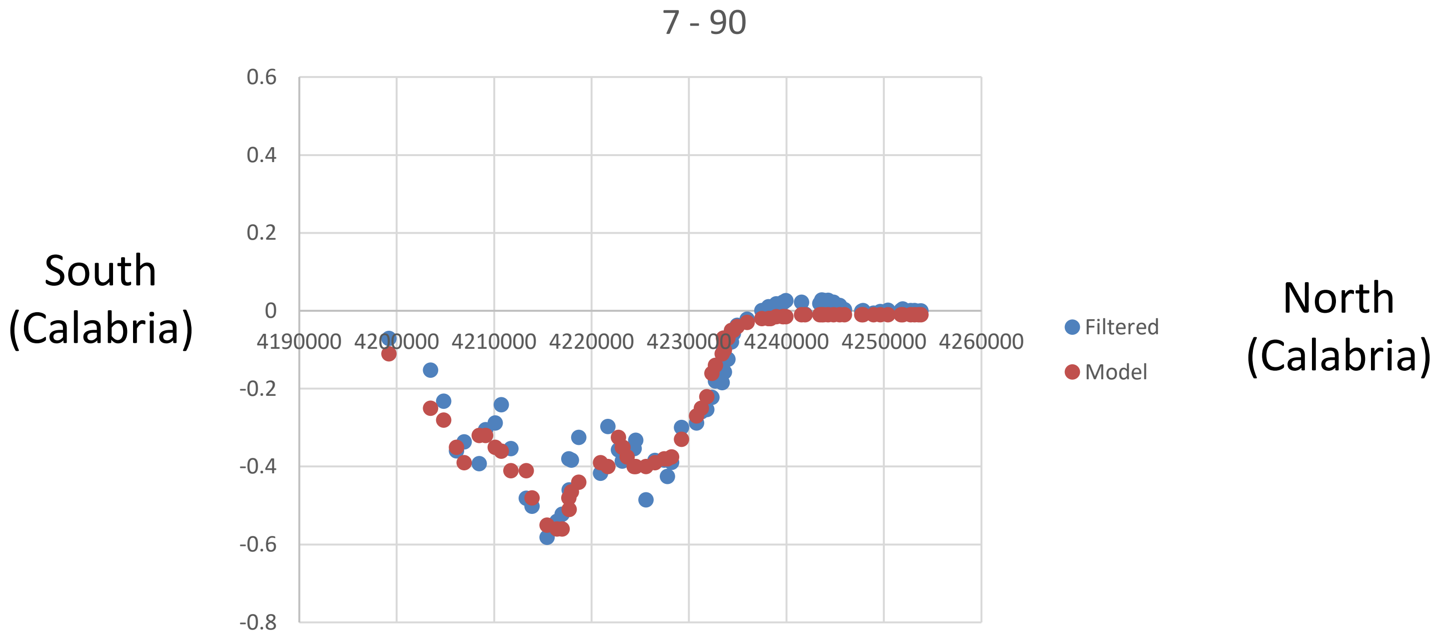

Slip = 7 m; Dip = 90 degrees

Low dipping angle (< 45 degrees)
East – West plots

Dip v Mean Misfit

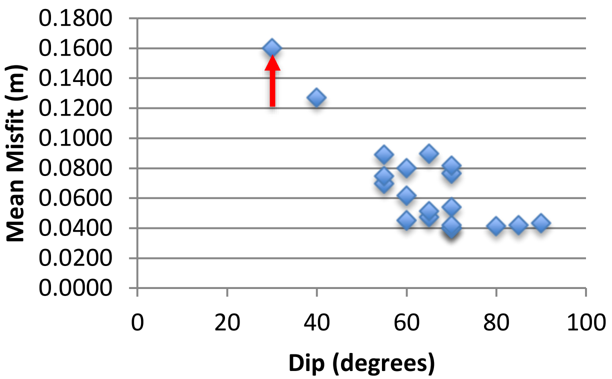

Slip v Mean Misfit

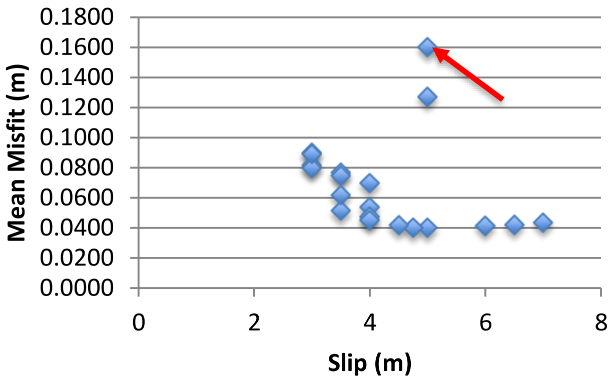

West (Sicily)

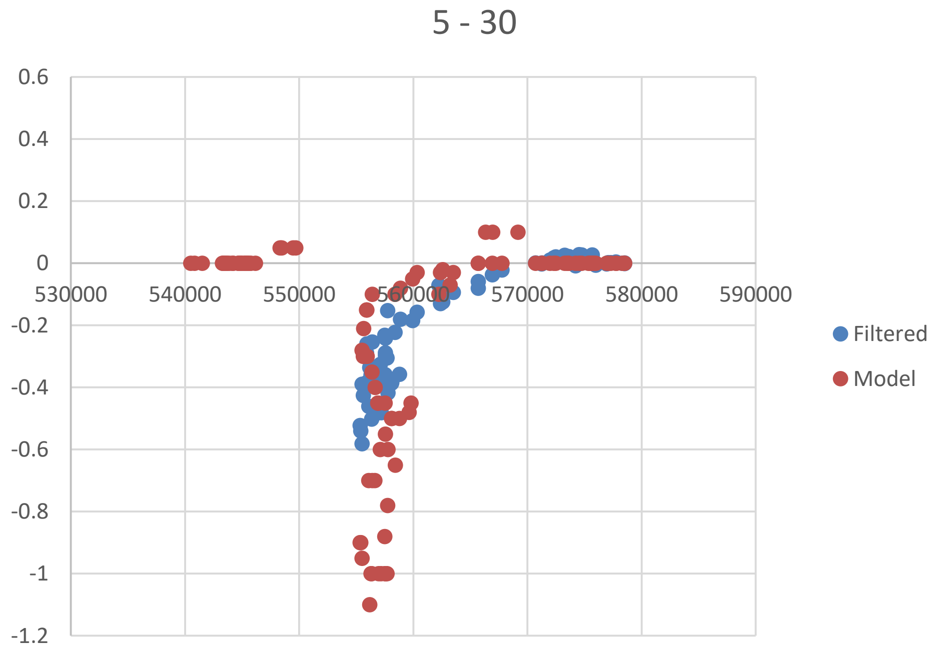

East (Calabria)

Slip = 5 m; Dip = 30 degrees

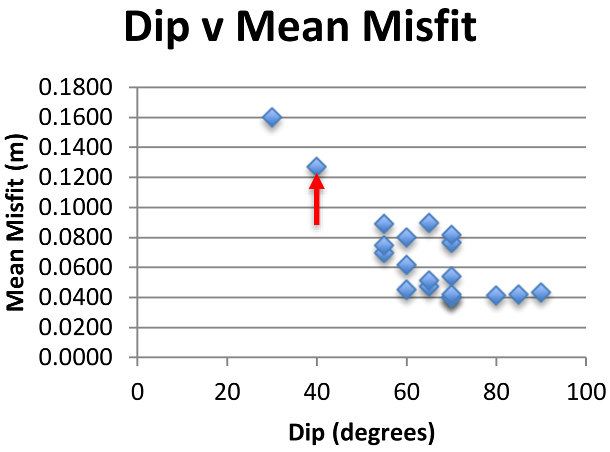

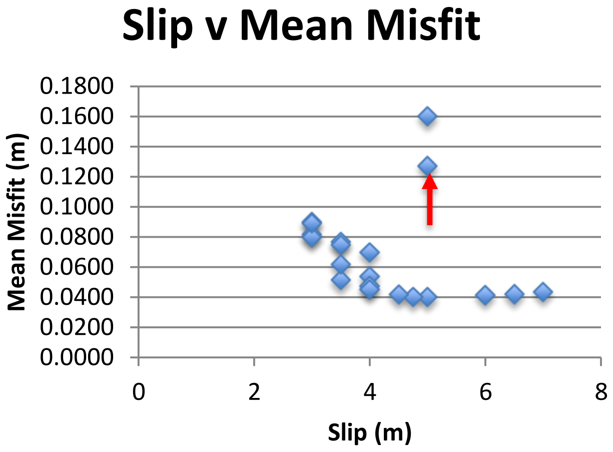

West
(Sicily)

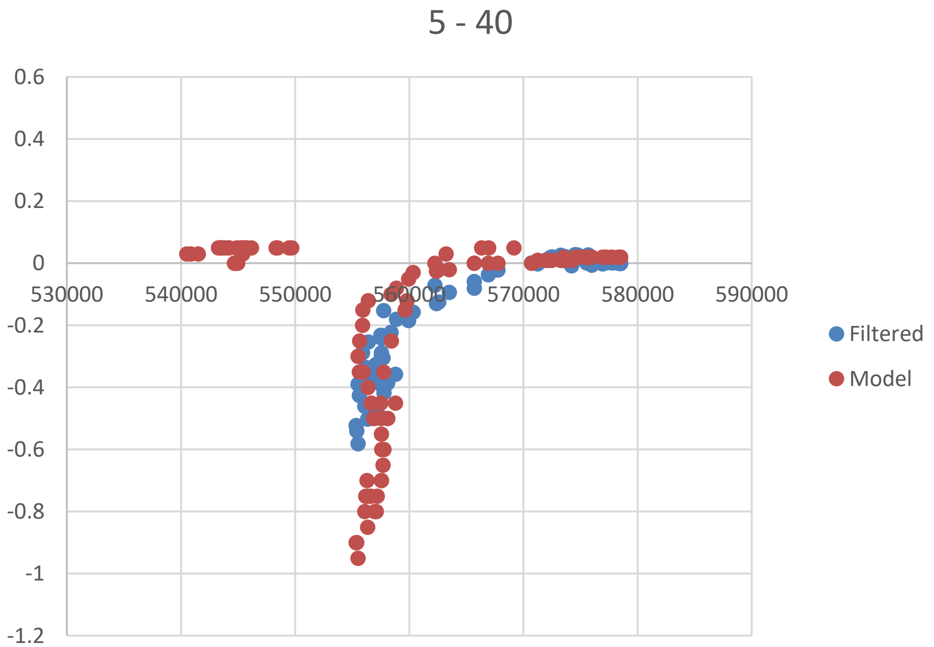

East
(Calabria)

Slip = 5 m; Dip = 40 degrees

Low dipping angle (< 45 degrees)
North – South plots

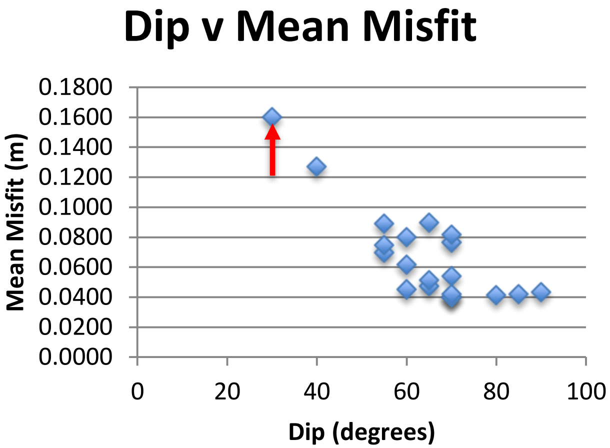

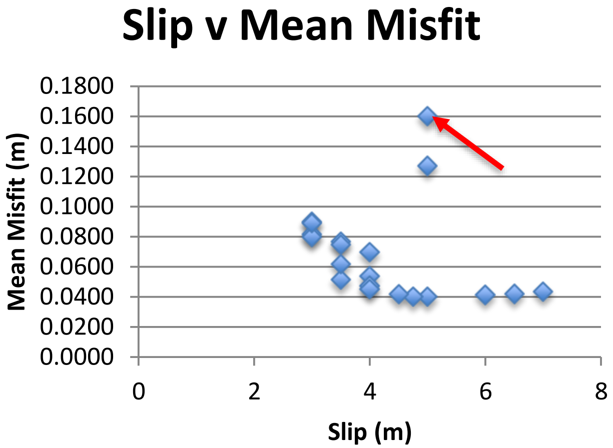

South
(Calabria)

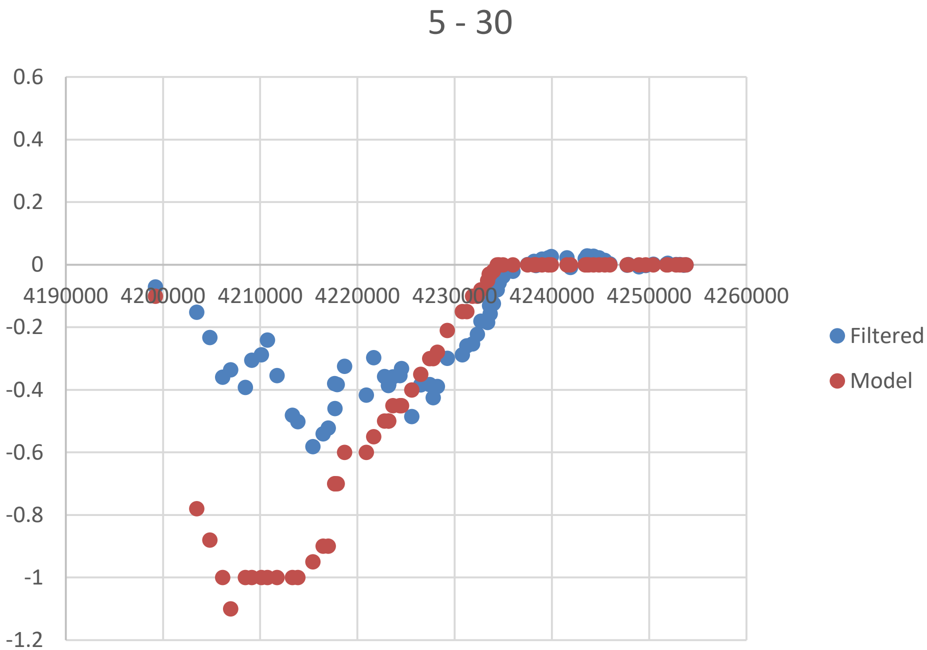

North
(Calabria)

Slip = 5 m; Dip = 30 degrees

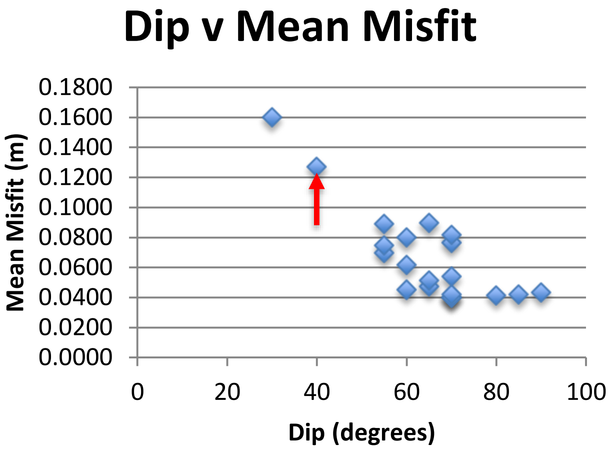

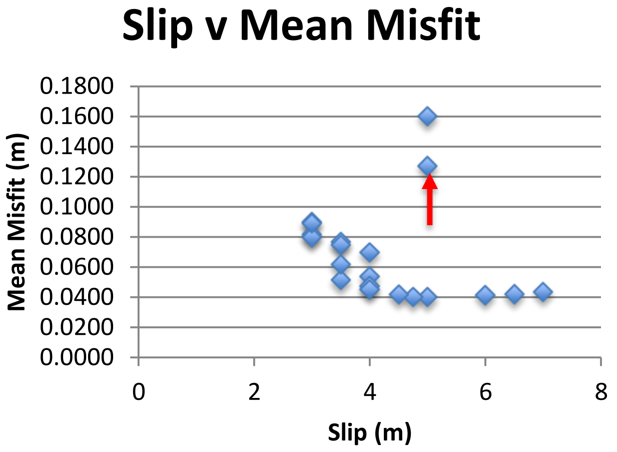

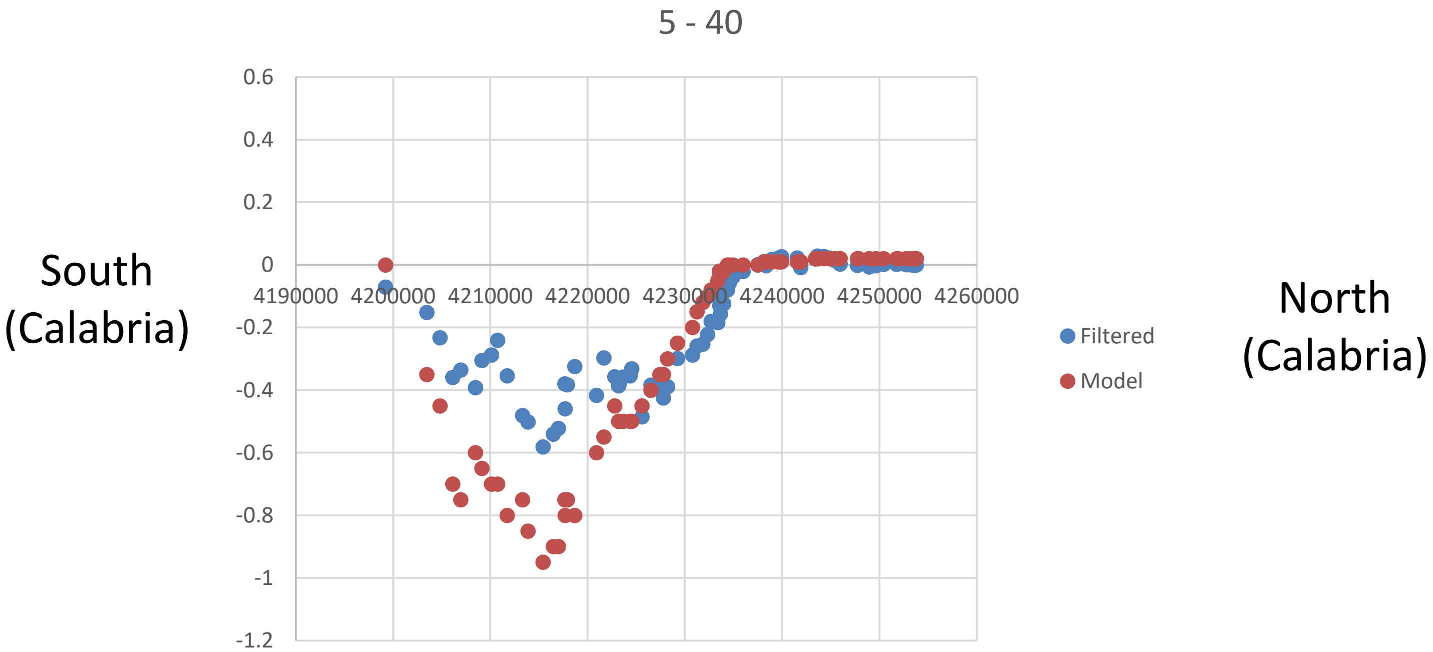

Slip = 5 m; Dip = 40 degrees

Slip on a mapped normal fault for the 28th December 1908 Messina earthquake (Mw 7.1) in Italy.

Meschis, M.[1*], Roberts, G. P.[1], Mildon, Z. K.[2], Robertson, J.[1], Michetti, A. M.[3], Faure Walker, J. P.[4]

Electronic Supplementary Material 3 (ESM3)

1- Department of Earth and Planetary Sciences, Birkbeck, University of London, UK
2- School of Geography, Earth and Environmental Sciences, University of Plymouth, UK
3- Università degli Studi dell'Insubria, Como, Italy
4- Institute for Risk and Disaster Reduction, UCL, London, UK
*corresponding author marco.meschis.14@ucl.ac.uk

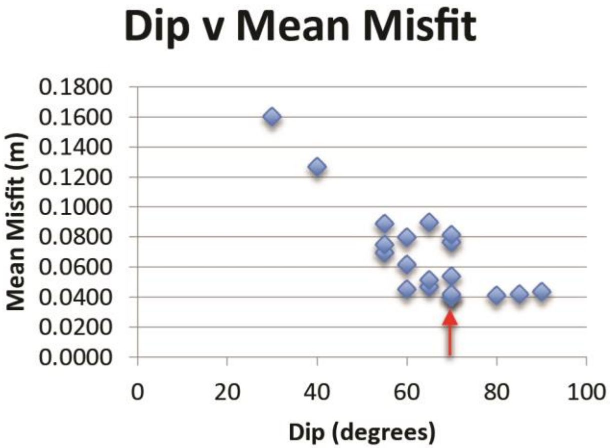

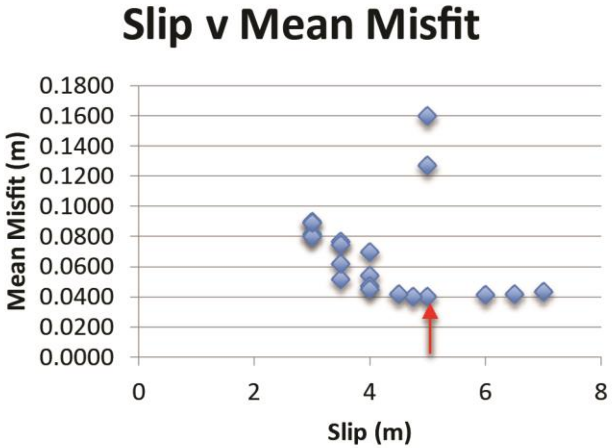

West (Sicily)

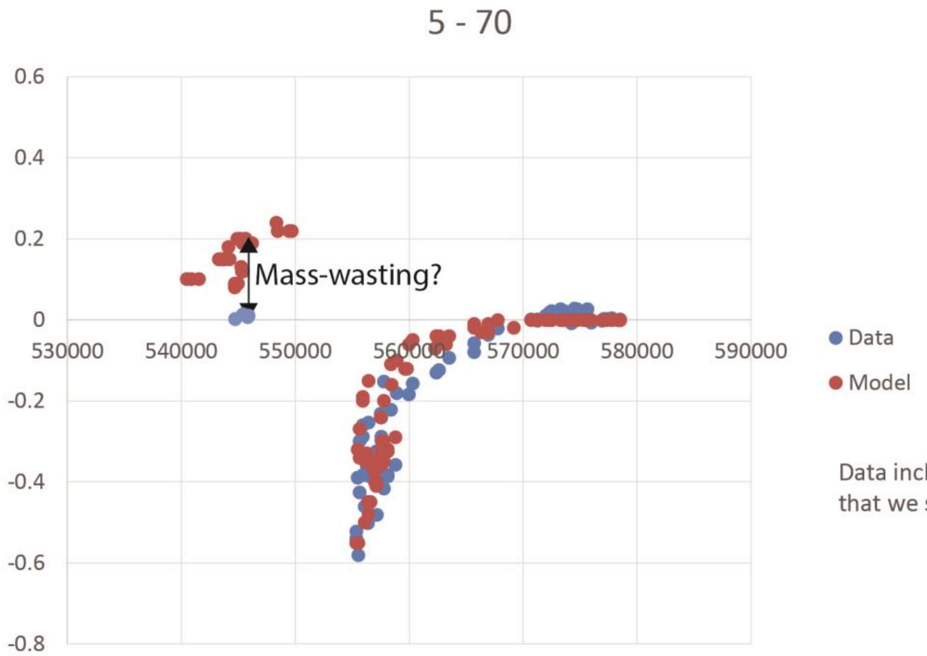

East (Calabria)

Data include uplifted benchmarks on the footwall that we suspect being affected by mass-wasting

Slip = 5 m; Dip = 70 degrees **Best fit model!!!**
